# Simulation of the Undiseased Human Cardiac Ventricular Action Potential: Model Formulation and Experimental Validation

**DOI:** 10.1371/journal.pcbi.1002061

**Published:** 2011-05-26

**Authors:** Thomas O'Hara, László Virág, András Varró, Yoram Rudy

**Affiliations:** 1 Cardiac Bioelectricity and Arrhythmia Center, Department of Biomedical Engineering, Washington University in St. Louis, St. Louis, Missouri, United States of America; 2 Department of Pharmacology and Pharmacotherapy, University of Szeged, Szeged, Hungary; 3 Division of Cardiovascular Pharmacology, Hungarian Academy of Sciences, Szeged, Hungary; University of California San Diego, United States of America

## Abstract

Cellular electrophysiology experiments, important for understanding cardiac arrhythmia mechanisms, are usually performed with channels expressed in non myocytes, or with non-human myocytes. Differences between cell types and species affect results. Thus, an accurate model for the undiseased human ventricular action potential (AP) which reproduces a broad range of physiological behaviors is needed. Such a model requires extensive experimental data, but essential elements have been unavailable. Here, we develop a human ventricular AP model using new undiseased human ventricular data: Ca^2+^ versus voltage dependent inactivation of L-type Ca^2+^ current (I_CaL_); kinetics for the transient outward, rapid delayed rectifier (I_Kr_), Na^+^/Ca^2+^ exchange (I_NaCa_), and inward rectifier currents; AP recordings at all physiological cycle lengths; and rate dependence and restitution of AP duration (APD) with and without a variety of specific channel blockers. Simulated APs reproduced the experimental AP morphology, APD rate dependence, and restitution. Using undiseased human mRNA and protein data, models for different transmural cell types were developed. Experiments for rate dependence of Ca^2+^ (including peak and decay) and intracellular sodium ([Na^+^]_i_) in undiseased human myocytes were quantitatively reproduced by the model. Early afterdepolarizations were induced by I_Kr_ block during slow pacing, and AP and Ca^2+^ alternans appeared at rates >200 bpm, as observed in the nonfailing human ventricle. Ca^2+^/calmodulin-dependent protein kinase II (CaMK) modulated rate dependence of Ca^2+^ cycling. I_NaCa_ linked Ca^2+^ alternation to AP alternans. CaMK suppression or SERCA upregulation eliminated alternans. Steady state APD rate dependence was caused primarily by changes in [Na^+^]_i_, via its modulation of the electrogenic Na^+^/K^+^ ATPase current. At fast pacing rates, late Na^+^ current and I_CaL_ were also contributors. APD shortening during restitution was primarily dependent on reduced late Na^+^ and I_CaL_ currents due to inactivation at short diastolic intervals, with additional contribution from elevated I_Kr_ due to incomplete deactivation.

## Author Summary

Understanding and preventing irregular heart rhythms that can lead to sudden death begins with basic research regarding single cell electrical behavior. Most of these studies are performed using non-human cells. However, differences between human and non-human cell properties affect experimental results and invoke different mechanisms of responses to heart rate changes and to drugs. Using essential and previously unavailable experimental data from human hearts, we developed and validated an accurate mathematical model of the human cardiac cell. We compared cellular behaviors and mechanisms to an extensive dataset including measurements from more than 100 undiseased human hearts. The model responds to pacing rate and premature beats as in experiments. At very fast pacing rates, beat to beat alternations in intracellular calcium concentration and electrophysiological behavior were observed as in human heart experiments. In presence of drug block, arrhythmic behavior was observed. The basis for these and other important rhythmic and irregular rhythm behaviors was investigated using the model.

## Introduction

The first step toward preventing sudden cardiac death is understanding the basic mechanisms of ventricular arrhythmias at the level of ion channel currents and the single myocyte action potential (AP), using both experiments[1] and theoretical models[2]. Obtaining ventricular myocytes from human hearts for the study of arrhythmia mechanisms is both rare and technically challenging. Consequently, these mechanisms are usually studied with human channels expressed in non myocytes, or with non human (rodent or other mammalian) myocytes. However, these approaches have limitations, because functionally important accessory subunits and anchoring proteins native to ventricular myocytes[3] are absent in expression systems, and even among mammalian ventricular myocytes, ion channel kinetics[4], [5], [6] and consequently arrhythmia mechanisms are strongly species dependent.

These issues limit the applicability of results from animal studies to human cardiac electrophysiology and clinical arrhythmia[7]. Measurements from undiseased human ventricular myocytes are a requisite for understanding human cell electrophysiology. Here, we present data from over 100 undiseased human hearts for steady state rate dependence, and restitution of the ventricular AP. Importantly, we also obtained essential new measurements for the L-type Ca^2+^ current, K^+^ currents, and Na^+^/Ca^2+^ exchange current from undiseased human ventricle. These previously unavailable data are critically important for correct formulation of mathematical models for simulation of electrophysiology and cellular arrhythmia mechanisms[8]. Using the new data together with previously published experiments, a detailed mathematical model of undiseased human ventricular myocyte electrophysiology and Ca^2+^ cycling was developed and thoroughly validated over the entire range of physiological frequencies. This model is referred to as the ORd (O'Hara-Rudy dynamic) model throughout the text. Model comparisons are conducted with the ten Tusscher-Panfilov (TP) model[9], and the Grandi-Bers (GB) model[10].

The ORd model was used to describe cellular electrophysiology mechanisms specific to human ventricular myocytes. Underlying mechanisms of AP duration (APD) rate dependence and APD restitution were investigated. The effects of Ca^2+^/calmodulin-dependent protein kinase II (CaMK) on known ionic current and Ca^2+^ cycling targets were incorporated and studied. Early afterdepolarizations (EADs) and alternans were reproduced by the model. These are important arrhythmogenic phenomena that must be reproduced in order to study the mechanisms of cardiac arrhythmias in human and simulate clinical interventions such as drugs.

## Results

Throughout, new undiseased human ventricle experimental data are represented by white circles or white squares for isolated myocyte or small tissue preparation measurements, respectively. Previously published nonfailing human ventricle experimental data are shown with black symbols. Other data classification schemes are provided case by case in figure legends.

### Formulation, Validation and Properties of Simulated Currents: L-type Ca^2+^ Current (I_CaL_)

**Figure 1:**
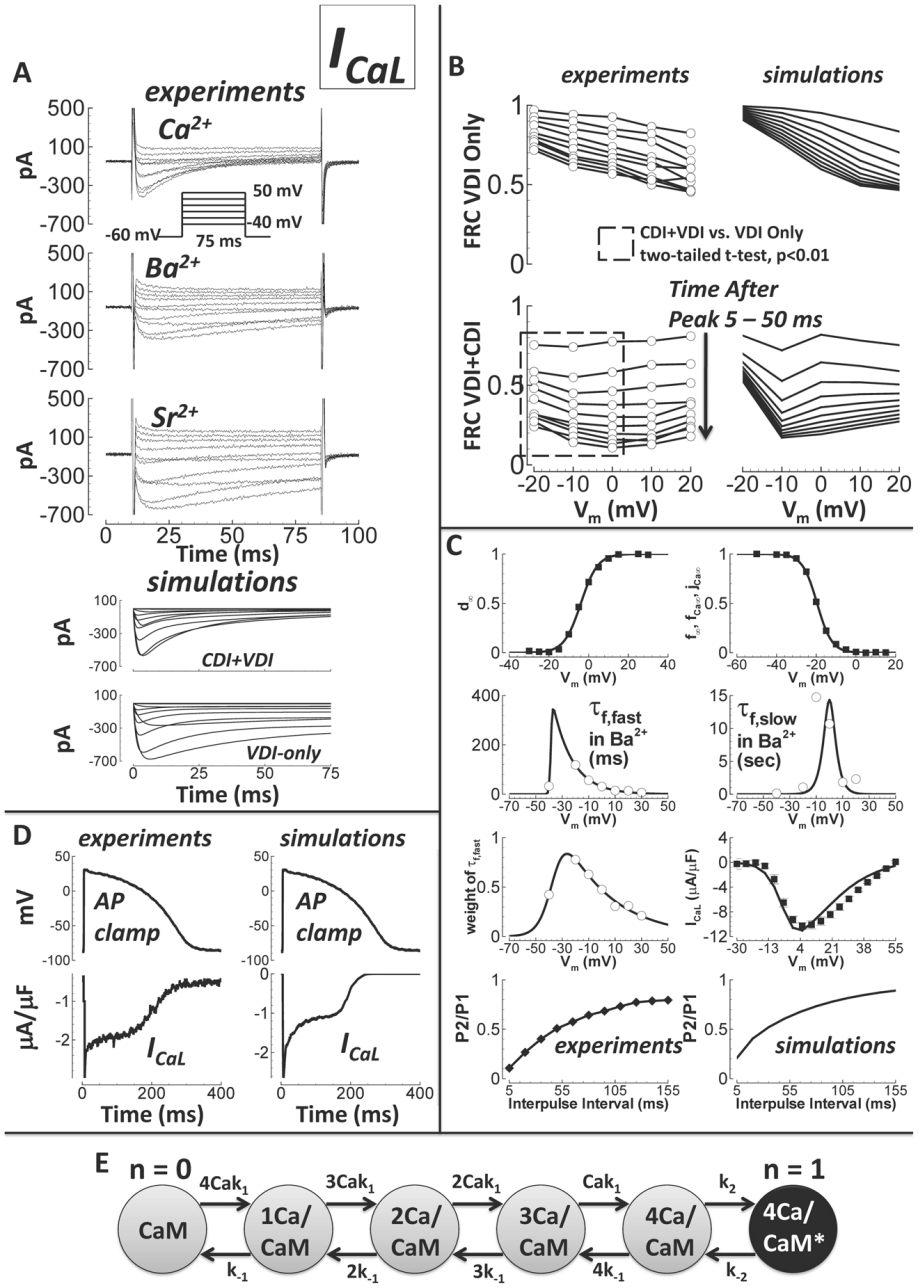
Undiseased human I_CaL_ experiments and model validation. A) Experiments: I_CaL_ traces for currents carried by Ca^2+^ (top), Ba^2+^ (middle), and Sr^2+^ (bottom). The voltage protocol is below the Ca^2+^ traces. Ca^2+^ current decay was visibly more rapid than decay for Ba^2+^ or Sr^2+^ currents. Simulations: I_CaL_ in response to the same voltage protocol with CDI (CDI+VDI, top), and without CDI (VDI-only, bottom). B) Experimental data are on the left (white circles, N = 5, from 3 hearts). Simulation results are on the right (solid lines). FRC is fractional remaining current. Times after peak current shown are from 5 to 55 ms, in 5 ms steps (indicated by arrow). Top left) Experiments showing the voltage and time dependence of FRC with Ba^2+^ as charge carrier (VDI only). Top right) Simulations of FRC, with n−gate = 0 (representing VDI only; see text and panel E). Bottom left) Experiments showing FRC with Ca^2+^ as charge carrier (CDI and VDI are concurrent). FRC for CDI+VDI was significantly smaller at more hyperpolarized potentials (V_m_ = −20 to 0 mV, dashed box) than FRC for VDI-alone. Bottom right) Simulations of FRC with free running n gate, allowing both CDI and VDI to occur. C) Data are from Magyar et al.[11] (black squares), Fulop et al.[12] (black diamonds), and previously unpublished (white circles, N = 5, from 3 hearts). Simulation results are solid lines. From left to right, top to bottom: steady state activation, steady state inactivation, fast time constant for VDI, slow time constant for VDI, relative weight of the fast component for VDI, I–V curve, experiments showing recovery from inactivation, and corresponding simulations. D) Human AP clamp waveform, used to elicit 1 µM nisoldipine sensitive current (I_CaL_, experiments, left) and comparison to simulations using the same AP clamp (right). E) Schematic diagram for the n gate, representing the fraction of L-type channels undergoing CDI. Calmodulin (CaM) is constitutively attached to the L-type channel. Ca^2+^ ions bind to CaM (on-rate k_1_ and off-rate k_-1_). With Ca^2+^ ions bound, the Ca^2+^/CaM/channel complex may activate CDI mode (asterisk and black color indicate CDI activation, on-rate k_2_ and off-rate k_-2_).

Data for I_CaL_ in the undiseased human ventricle are from Magyar et al.[11] and Fulop et al.[12] (both at 37°C, model validation in Figure 1C). Magyar et al. measured steady state activation, steady state inactivation, and the current voltage (I–V) curve. Fulop et al. measured recovery from inactivation. However, neither study separated Ca^2+^ dependent inactivation (CDI) from voltage dependent inactivation (VDI). In fact, no published data are available which separate CDI and VDI in the undiseased or nonfailing human ventricle. For this measurement, we made new recordings in undiseased human ventricular myocytes at 37°C (Figure 1, current traces and white circles).

Measurements were carried out with Ca^2+^ as charge carrier, allowing both CDI and VDI, or with Ba^2+^ as charge carrier, allowing only VDI. Results for Sr^2+^ were similar to those for Ba^2+^. From holding potential of −60 mV, 75 ms steps were to potentials ranging from −40 to +50 mV (10 mV increments, 3 second interpulse interval, Figure 1A). 75 ms was sufficient for comparison of CDI and VDI, since it is in the early phase of decay in which CDI effects are most prominent[13]. Simulated current traces for CDI+VDI and for VDI–alone were similar to the experiments.

Fractional remaining current (FRC, at time t and voltage V_m_, FRC(t,V_m_) = I(t,V_m_)/I_peak_(V_m_)) quantified the voltage and time dependence of inactivation for comparison between charge carriers. Figure 1B compares FRC for Ba^2+^ (experiments top left, simulations right), and Ca^2+^ (experiments bottom left, simulations right). With Ba^2+^ as the charge carrier, FRC monotonically decreased with increasing voltage at all times after peak current. This finding is consistent with dependence of inactivation on voltage alone. In contrast, for Ca^2+^ currents, FRC did not decrease monotonically with increasing voltage. Rather, Ca^2+^ current FRC curves appear to be effectively voltage independent. FRC for CDI+VDI was statistically different from FRC for VDI-alone at the more hyperpolarized potentials (−20 to 0 mV, unpaired two-tailed t-test, p<0.01). Ca^2+^ ions caused additional inactivation at these voltages, where VDI-alone was relatively weak. Since the only difference between Ca^2+^ and Ba^2+^ cases was the charge carrier, it follows that Ca^2+^ ions themselves were the source of the additional inactivation. This is evidence that currents carried by Ba^2+^ inactivate due to VDI only, while Ca^2+^ currents inactivate due to both VDI and CDI[14]. There is evidence that Ba^2+^ can cause ion dependent inactivation[15]. However, Ba^2+^-dependent inactivation was estimated to be 100-fold weaker than CDI[16], and its effects were not appreciable in FRC experiments.

To modulate VDI versus CDI in the model, the n gate was introduced, the value of which represents the fraction of channels operating in CDI mode. Under physiological conditions, I_CaL_ inactivation is caused by a combination of both CDI and VDI. That is, n is between 0 (all VDI) and 1 (all CDI). This model was based on experiments by Kim et al.[17], where CDI was observed to function as a faster VDI, activated by elevated Ca^2+^. Thus, both CDI and VDI are voltage dependent. The rate of decay in CDI mode is faster than that in VDI mode. The Mahajan et al.[18] and Decker et al.[19] I_CaL_ models work similarly.

The n gate is diagrammed in Figure 1E. Rates k_1_ and k_-1_ represent binding/unbinding of Ca^2+^ to channel bound calmodulin (CaM)[20]. There are four identical binding sites. Rates k_2_ and k_-2_ represent activation/deactivation of CDI mode (black circle, asterisk), which occurs when all Ca^2+^ binding sites are occupied. We considered that the four Ca^2+^ binding transitions are in rapid equilibrium and solved the reversible two state reaction of Ca^2+^/CaM binding and CDI mode activation to obtain the differential equation describing the n gate (Supplement Text S1, page 10).

In both CDI and VDI modes, there are two weighted time constants for inactivation (time constant weighting described in Methods). We determined time constants for CDI and n gate kinetics in an attempt to represent the shape and magnitude of the FRC measurements (i.e. CDI reduced FRC, particularly at negative potentials). Time constants for VDI gates were determined by inactivation of Ba^2+^ currents (Figure 1C). AP clamp simulations using the formulated I_CaL_ model were similar to AP clamp experiments, where I_CaL_ was defined as the 1 µM nisoldipine sensitive current (Figure 1D). Specifically, currents showed spike and dome morphology. In experiments, peak current was −3.0 µA/μF. It was −2.7 µA/µF in simulations. Fast inactivation was 2.5 fold faster when phosphorylated by CaMK, similar to the Decker et al. dog I_CaL_ model[19] and as measured experimentally[21].

### Transient Outward K^+^ Current (I_to_)

**Figure 2:**
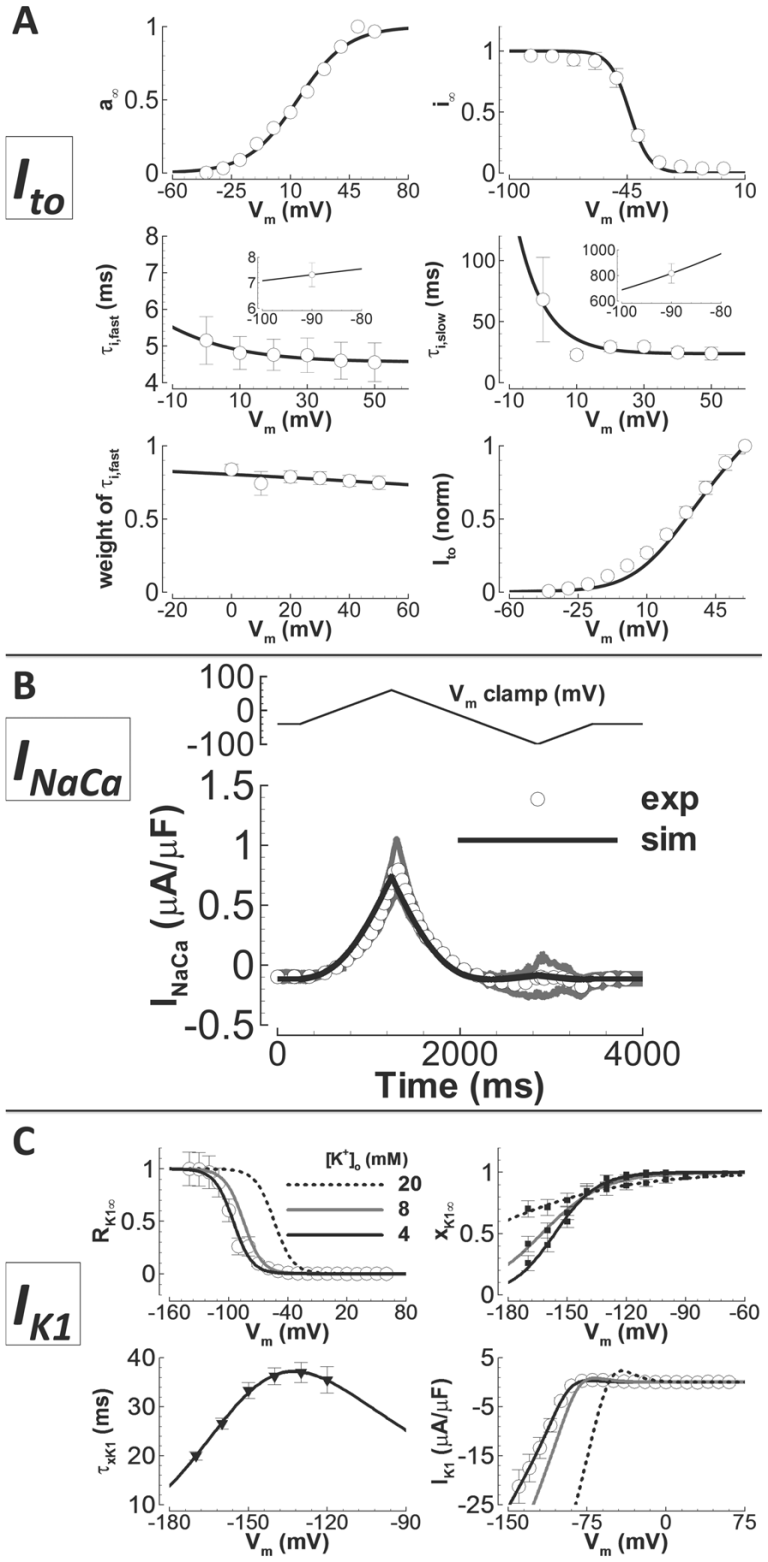
Undiseased human I_to_, I_NaCa_, and I_K1_ experiments and model validation. A) I_to_. Experimental data are white circles (N = 8 from 5 hearts for inactivation time constants, N = 10 from 5 hearts for recovery time constants, N = 9 from 6 hearts for steady state inactivation, and N = 23 from 8 hearts for the I–V curve). Simulation results are solid lines. From left to right, top to bottom: steady state activation, steady state inactivation, fast time constant for inactivation, slow time constant for inactivation (insets show fast and slow recovery from inactivation), relative weight of the fast component for inactivation and the I–V curve (normalized). B) I_NaCa_. Experimental data are digitally averaged time traces (N = 3 from 2 hearts, white circles, gray is standard error of the mean). Simulation results are the solid line. Top) Voltage clamp protocol. Bottom) I_NaCa_ in response to the clamp. C) I_K1_. Experimental data are previously unpublished (white circles, N = 21 from 12 hearts), from Bailly et al.[37] (black squares) and Konarzewska et al.[38](black triangles). Simulation results are solid lines (black, gray and dashed black for [K^+^]_o_ = 4, 8 and 20 mM). Top left) Voltage and [K^+^]_o_ dependence of steady state rectification. Top right) Voltage and [K^+^]_o_ dependence of steady state inactivation. Bottom left) Time constant for inactivation. Bottom right) I–V curve, and its [K^+^]_o_ dependence.

The model for I_to_ was formulated based on newly measured experimental data. The measurements were from isolated undiseased human ventricular myocytes at 37°C (Figure 2A, white circles), and were carried out with the addition of 1 µM nisoldipine to the standard bath solution (see Methods) to block I_CaL_. The holding potential was −90 mV. Currents were activated by a 300 ms step to various potentials. Inactivation time constants were determined from exponential fits to decay of these traces. To measure steady state inactivation, 500 ms steps from −90 mV to various potentials were followed by test pulses to 50 mV. Recovery from inactivation was determined at −90 mV, using P1/P2 pulses of 200 ms to 50 mV at varying interpulse intervals in a double pulse protocol.

The time constant for activation was determined by fitting time to peak from a digitized current trace (Amos et al.[22], their Figure 12C, in undiseased human ventricle at 37°C; τ_a_ = 2.645 ms at V_m_ = +40 mV). Greenstein et al.[23] showed time to peak for hKv4.3 expressed in mouse fibroblast cells. The model provides a qualitative match to these data (considering temperature and expression system differences). That is, the model activation time constant decreases from a peak value of 6.5 to 1.5 ms in near linear fashion with increasing voltage from −20 to 60 mV.

The inactivation gate has two time constants, each with voltage dependent weighting. Inactivation kinetics and the I–V curve are accurate to the experimental data. A small divergence between simulations and experiments was observed at hyperpolarized potentials along the I–V curve (simulated current was less than in experiments). This may be due to the fact that experimentally measured currents were small and difficult to measure at these potentials. In fact, current was not measureable in 21, 11, 5, and 1 out of 23 cells at V_m_ = −40, −30, −20, and −10 mV, respectively. Currents with zero values were not included in the experimental I–V averages. However, these currents were included in averages for obtaining steady state activation and steady state inactivation curves in the model. This prevented over representation of the window current (small, appearing late during phase-3 of the AP, shown later). The conductance of the I_to_ model was set so that phase-1 behavior of the simulated AP would be similar to undiseased human endocardium experiments (small in endocardium; maximum value ∼1 µA/µF). Measured endocardial APs showed rapid phase-1 repolarization, but did not show positive time derivatives during phase-1 (true notching was generally not observed). Thus, model I_to_ conductance was set to the maximum level which did not violate these observations (∼1 µA/µF peak current at 1 Hz pacing).

CaMK effects on I_to_ were incorporated based on measurements by Tessier et al.[24] and Wagner et al.[25]. As in Tessier et al., CaMK shifted the voltage dependence of steady state activation 10 mV in the depolarization direction, and the time constant for development of inactivation was increased (multiplicative factor fit to match the voltage dependent increase). Wagner et al. showed that the time constant for recovery from inactivation was affected by CaMK (∼2 fold faster).

### Na^+^/Ca^2+^ Exchange Current (I_NaCa_)

The I_NaCa_ model was formulated using measurements from undiseased human ventricular myocytes at 37°C (Figure 2B, white circles). The model was based on the framework established by Kang and Hilgemann[26], which allows for unlikely occurrence of inward Na^+^ leak, without Ca^2+^ exchange. The Hilgemann model shows Na^+^:Ca^2+^ exchange stoichiometry slightly greater than 3.0, as has been observed by others[27], [28]. Though the Hilgemann model is mechanistically novel in this way, it can still reproduce all Na^+^, Ca^2+^ and voltage dependent properties observed by Weber et al.[29] in the nonfailing human ventricle. Compare Hilgemann and Weber data to our simulated reproductions in Supplement Figures S1, S2 and S3 in Text S1. As in the Faber-Rudy[30] and Hund-Decker-Rudy models[19], [31], we included 20% of the exchanger in the Ca^2+^ diffusion subspace[32], [33]. The choice to include 20% in the subspace in human is validated based on its effect on the rate dependence of peak [Ca^2+^]_i_ (results in Supplement Figure S17 in Text S1). Values above or below 20% disrupt the demonstrated correspondence of peak [Ca^2+^]_i_ rate dependence with experiments (see section on Na^+^ and Ca^2+^ rate dependence).

### Inward Rectifier K^+^ Current (I_K1_)

The model for I_K1_ was constructed based primarily on new experimental data, measured at 37°C in undiseased isolated human ventricular myocytes as the 0.5 mM BaCl_2_ sensitive current (Figure 2C, white circles). Current was elicited with steps from −90 mV to various potentials for 300 ms. The current that remained at the end of the steps was recorded as I_K1_.

Two gates were used in the model: R_K1_, the instantaneous rectification gate, and x_K1_, the time dependent inactivation gate. Importantly, previous models[9], [10], [34], [35] have ignored both inactivation gating, and detailed [K^+^]_o_-dependence of I_K1_ (exception, I_K1_ equations by Fink et al.[36]). There are nonfailing human ventricular measurements which we utilized to include these effects[37], [38].

Steady state rectification was determined by dividing current by driving force, then normalizing. Rectification was shown to be [K^+^]_o_-dependent in the nonfailing human ventricle by Bailly et al.[37]. A linear shift in V_1/2_ for rectification toward more depolarized potentials with elevated [K^+^]_o_ was incorporated, as was shown experimentally (compare to Bailly et al., their Figure 4B). Bailly also showed the voltage and [K^+^]_o_-dependence of inactivation. We introduced the time dependent x_K1_ gate, based on these data. As was shown experimentally, both V_1/2_ and the slope factor for inactivation depend linearly on [K^+^]_o_. The time constant for inactivation was based on measurements in nonfailing human ventricular myocytes by Konarzewska et al.[38] (their Figure 1C). Conductance was observed to be in proportion to the square root of [K^+^]_o_ in the human ventricle[37]. When assembled, the I_K1_ model demonstrated correspondence with the measured amplitude and rectification profile, and with Bailly data for [K^+^]_o_-dependence. As in Jost et al.[39], I_K1_ was voltage dependent, but not pacing rate dependent (Supplement Figure S4 in Text S1).

### Rapid Delayed Rectifier K^+^ Current (I_Kr_)

**Figure 3:**
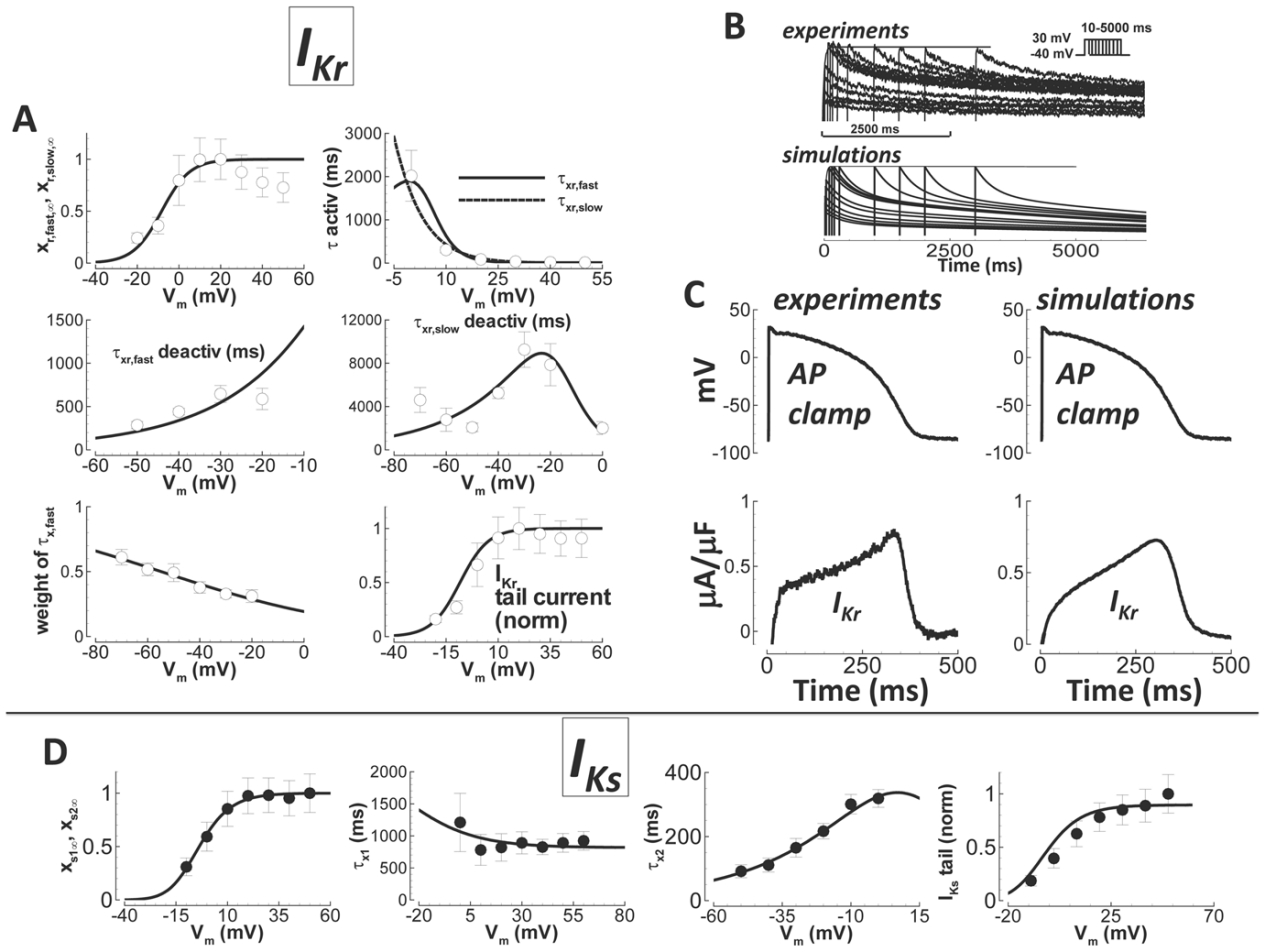
Undiseased human I_Kr_ and I_Ks_ experiments and model validation. A) I_Kr_. Experimental data are white circles (N = 10 from 7 hearts for steady state activation, N = 7 from 3 hearts for activation and from 2 hearts for deactivation time constants and weights, and N = 10 from 7 hearts for tail currents). Simulation results are lines. From left to right, top to bottom: steady state activation, time constant for activation (fast (solid) and slow (dashed) time constants converge), fast time constant for deactivation, slow time constant for deactivation, relative weight of the fast component for deactivation, and the I–V curve for normalized tail currents. B) Activation/deactivation profiles in response to the voltage steps shown (−40 mV holding potential to +30 mV steps of various duration, followed by a return to −40 mV, top right inset). Experiments are above. Simulations are below. Activation is rapid, occurring within tens of milliseconds. Deactivation is slow, occurring after several seconds. C) Human AP clamp waveform (top), used to elicit 1 µM E-4031 sensitive current (I_Kr_, bottom); experiments are on the left, and comparison to simulations using the same AP clamp is on the right. D) I_Ks_. Data are from Virág et al.[41] (black circles). Simulation results are solid lines. From left to right: steady state activation, time constant for activation (much slower than deactivation at depolarized potentials), time constant for deactivation (much faster than activation at hyperpolarized potentials), and the I–V curve, showing normalized tail currents.

The model for I_Kr_ was constructed using experimental data measured in isolated undiseased human ventricular myocytes at 37°C (Figure 3A, white circles). Measurements were carried out with/without addition of 1 µM E-4031 to the standard bath solution in order to obtain the difference current. Tail currents were elicited by stepping from −40 mV to various potentials for 1000 ms, and then stepping back down to −40 mV. The deactivation time constant was determined by fitting the tail current decay. The time constant for activation was found by stepping from −40 mV to various potentials for various durations preceding a step back to −40 mV. The rate with which the envelope of tail currents developed at different voltages was measured with an exponential fit to obtain the time constant for activation. Since this process was well fit by a single exponential, we made the fast and slow time constants in the model converge on the activation limb, at depolarized potentials. The steady state activation curve was determined from the I–V curve, after dividing by the driving force, assuming maximal activation at the time of peak tail current. Slow deactivation of I_Kr_ (experiments and simulations, Figure 3B), suggests its participation in AP shortening during steady state pacing at fast rate and at short diastolic intervals during restitution; this hypothesis will be explored in a later section. The fast inactivation (rectification, instantaneous in the model) R_Kr_ gate was determined so that current profile matched experiments using a human AP voltage clamp (Figure 3C). Important features of the experimental AP clamp trace that the model reproduced include 1) the early recovery phase, where approximately half maximal current appeared by the beginning of the AP plateau, followed by 2) quasi-linear current increase until peak current was reached during late phase-3 of the AP.

Since enzymes used to disaggregate myocytes can significantly degrade I_Kr_ [40], conductance was scaled to provide correct APD90 in control and with I_Kr_ block, measured in small tissue preparations. Indeed, APD90 was a function of I_Kr_ conductance (parameter sensitivity, Supplement Figure S15 in Text S1). As in undiseased human ventricle experiments[39], I_Kr_ was voltage dependent, but not pacing rate dependent (Supplement Figure S5 in Text S1).

### Slow Delayed Rectifier K^+^ Current (I_Ks_)

Data from Virág et al.[41], measured in isolated undiseased human ventricular myocytes at 37°C, were used to construct the model for I_Ks_ (Figure 3D). The model has two gates: x_s1_ and x_s2_. The x_s1_ gate is responsible for activation. Deactivation was controlled by x_s2_. Activation/deactivation separation was based on the fact that activation was much slower than deactivation. Setting τ_x1_>>τ_x2_ at hyperpolarized potentials, where deactivation dominated, and τ_x2_<<τ_x1_ at depolarized potentials, where activation dominated, allowed for separation of these processes as two gates. As in the case of I_Kr_, it is understood that I_Ks_ is damaged (reduced) by enzymatic disaggregation of myocytes[42]. Therefore, we used I_Ks_ specific drug block (1 µM HMR-1556) effects on APD90, measured in small tissue preparations, to determine the correct conductance. Ca^2+^ dependence of I_Ks_ was incorporated based on measurements by Tohse et al.[43]. The effect of this dependence was negligible under physiological Ca^2+^ concentration conditions.

### Fast Na^+^ Current

**Figure 4:**
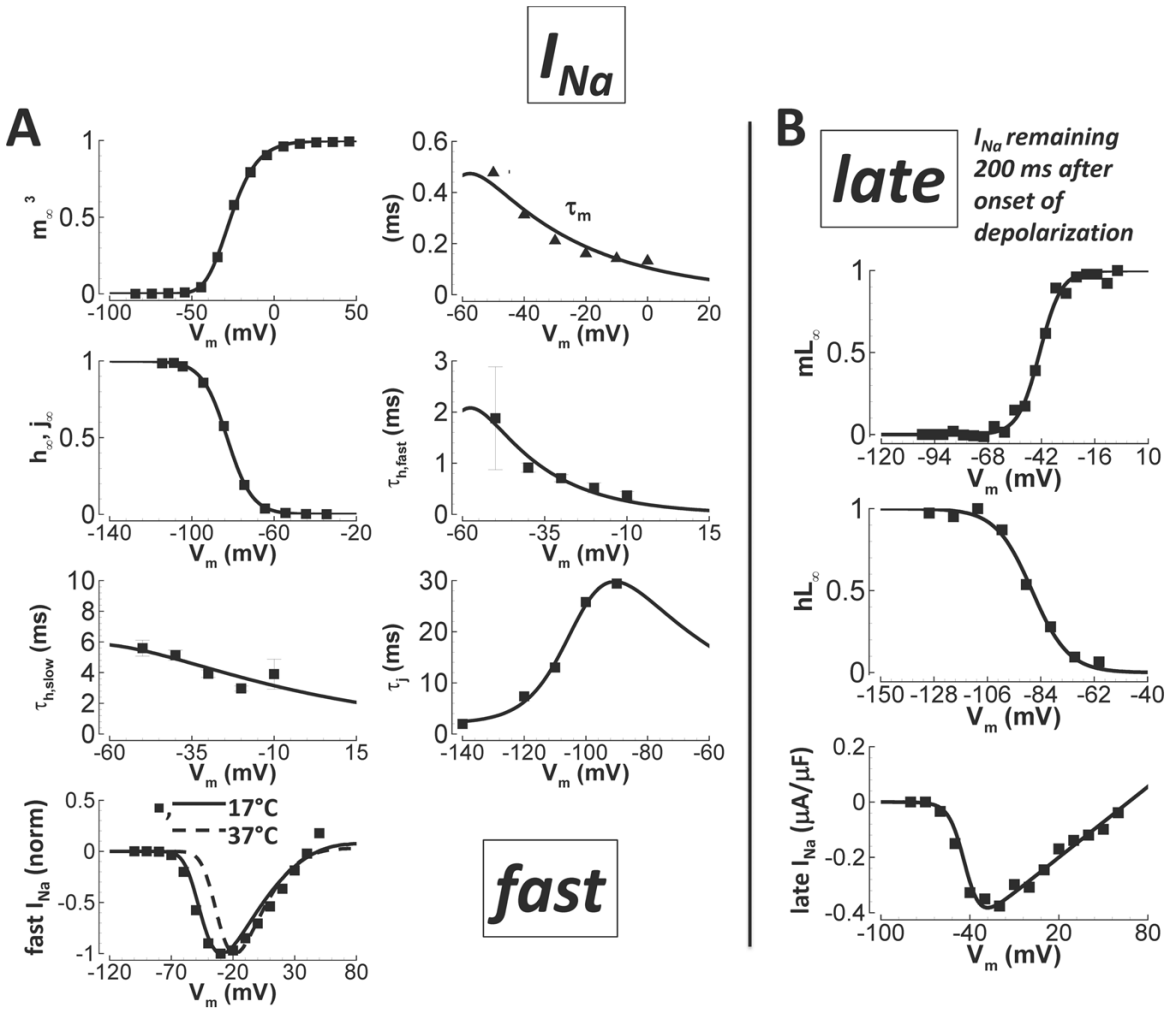
Nonfailing human fast I_Na_ and late I_Na_ experiments and model validation. A) Fast I_Na_. Experiments are from Sakakibara et al.[44] (black squares) and Nagatomo et al.[45] (black triangles). Simulation results are solid lines. From left to right, top to bottom: steady state activation, time to peak (experiment) and activation time constant (simulation), steady state inactivation, fast time constant for development of inactivation, slow time constant for development of inactivation, time constant for recovery from inactivation, and the I–V curve (solid line simulation and data at 17°C, dashed line simulation at 37°C). In other panels, simulations and data were adjusted to 37°C. Time to peak was fit at 33°C. B) Late I_Na_. Experiments are from Maltsev et al.[51] (black squares). Simulation results are solid lines. Top) Steady state activation. Middle) Steady state inactivation. Bottom) I–V curve.

Fast I_Na_ was formulated using nonfailing human ventricular data from Sakakibara et al.[44] (Figure 4A). Since Sakakibara experiments were performed at 17°C, a temperature adjustment was used to obtain the final model equations, representing behavior at 37°C. The effect of temperature on steady state gating was shown by Nagatomo et al.[45]. For activation, V_1/2_ shift with temperature change from 23 to 33°C was +4.3 mV. For inactivation, the shift was +4.7 mV. We shifted V_1/2_ by twice these amounts, assuming linearity (adjust to 37°C from data taken at 17°C, a change of 20°C; Nagatomo showed a change of 10°C)_._ Time constants were adjusted to 37°C using Q_10_. We set Q_10_ = 2 since Q_10_ was given as “about two” by Nagatomo.

Hanck and Sheets[46] documented a shift in V_1/2_ with the passage of time after patch clamp commencement. For activation, the shift was −0.47 mV/min. It was −0.41 mV/min for inactivation. Sakakibara reported the time elapsed between patching and measurement for steady state activation and inactivation as between 10 and 20 min, ∼15 min for both. Thus, we reversed the time dependent shifts in V_1/2_.

CaMK effects on I_Na_ were based on available data[47]. We took into account the measured −6.2 mV V_1/2_ shift in steady state inactivation, the roughly 3-fold slowing of current decay, and the 1.46-fold slowing of recovery from inactivation.

The non-temperature adjusted model I–V curve matches Sakakibara data at 17°C. We determined appropriate channel conductance at 37°C based on conduction velocity, and maximum dV_m_/dt. Conduction velocity in a one dimensional fiber simulation was 45 cm/s during 1 Hz pacing, consistent with available (dog) experiments[48]. It was 70 cm/s when stimulated from quiescence, consistent with *in vivo* measurements in nonfailing human hearts[49]. Maximum dV_m_/dt was 254 mV/ms in single cells at 1 Hz pacing, consistent with measurements from nonfailing human ventricular myocytes at 37°C (234±28 mV/ms)[50].

### Late Na^+^ Current

Data used in the formulation of late I_Na_ were from Maltsev et al.[51], measured in the nonfailing human ventricle (Figure 4B), functionally defined in experiments and simulations as the Na^+^ current remaining after 200 ms from the onset of depolarization. Steady state activation was derived from the I–V curve (current divided by driving force, then normalized). The time constant for activation of late I_Na_ was identical to that for fast I_Na_. It is not possible to measure the time to peak for late I_Na_ because of the interfering effects of the much larger I_Na_. However, in the model scheme, the measurement is irrelevant for the same reason.

The h_L_ gate is responsible for both development of and recovery from inactivation. The time constant for development was adjusted using Q_10_ = 2.2, as measured by Maltsev et al.[52] (hNav1.5 channels expressed heterologously). The time constant was voltage independent[51]. Maltsev et al.[51] reported a maximum late I_Na_ of −0.356 pA/pF in nonfailing human ventricular myocytes (average current between 200 and 220 ms during step to −30 mV from −120 mV, their Table 2, donor heart average). We scaled the Maltsev I–V curve to the donor value and used it to determine the model conductance.

We do not consider fast and late Na^+^ currents to be separate channels. Rather, they have long been understood to represent different gating modes (experiments[52], and simulations by our group[53]), separated functionally in time. In experiments, and in simulated reproductions of experiments, late I_Na_ was functionally defined as the I_Na_ current persisting 200 ms after onset of depolarization. CaMK dependence was implemented (−6.2 mV V_1/2_ shift in steady state inactivation, and 3-fold slowing of inactivation time constant, as measured[47]).

### Na^+^/K^+^ ATPase Current (I_NaK_)

The model for I_NaK_ was reformulated based on the work of Smith and Crampin[54]. The Smith and Crampin model includes more detail than standard formulations employed by other ventricular AP models[9], [10], [34], [35]. Importantly, the Smith and Crampin framework includes [K^+^]_i_ dependence and inputs for ATP and pH sensitivity. Here, we set ATP and pH values to normal physiological levels (pH was dynamic when stated). Dynamically changing [K^+^]_i_ is a known and meaningful pump regulator that is a functioning part of this model. High [K^+^]_i_ (combined with low ATP) can make the pump reverse, bringing Na^+^ in, as has been observed in isolated hearts[55].

The Smith and Crampin model (schematized in Supplement Figure S6 in Text S1) was adjusted to reproduce the basic findings of Nakao and Gadsby[56], demonstrating [Na^+^]_o_ dependence, [Na^+^]_i_ dependence with high and low [Na^+^]_o_, and [K^+^]_o_ dependence with high and low [Na^+^]_o_ (Supplement Figure S7 in Text S1). To determine human ventricle appropriate conductance for I_NaK_, we used [Na^+^]_i_-frequency data presented by Pieske et al.[57] as a target (nonfailing human left ventricular myocytes at 37°C).

The I_NaK_ formulation is based on known biophysical properties[54]; its behavior reproduces available experimental observations[56] (Supplement Figure S7 in Text S1). However, no direct measurement of I_NaK_ has been made in the nonfailing or undiseased human ventricle. To endow human ventricle specificity to I_NaK_, our strategy was indirect; reproducing the rate dependence of intracellular Na^+^ concentration, [Na^+^]_i_, measured in the nonfailing human ventricle was the target. This choice assumes that the major role for I_NaK_ is maintenance of physiological [Na^+^]_i_. In the model, [Na^+^]_i_ and its relative changes with pacing rate are controlled by I_NaK_ conductance (∼0.5 mM change per 20% change in I_NaK_ conductance, Supplement Figure S18 in Text S1). In the absence of direct human ventricle I_NaK_ measurements, validation of the I_NaK_ formulation employs this relationship.

### Human AP Characteristics and APD

**Figure 5:**
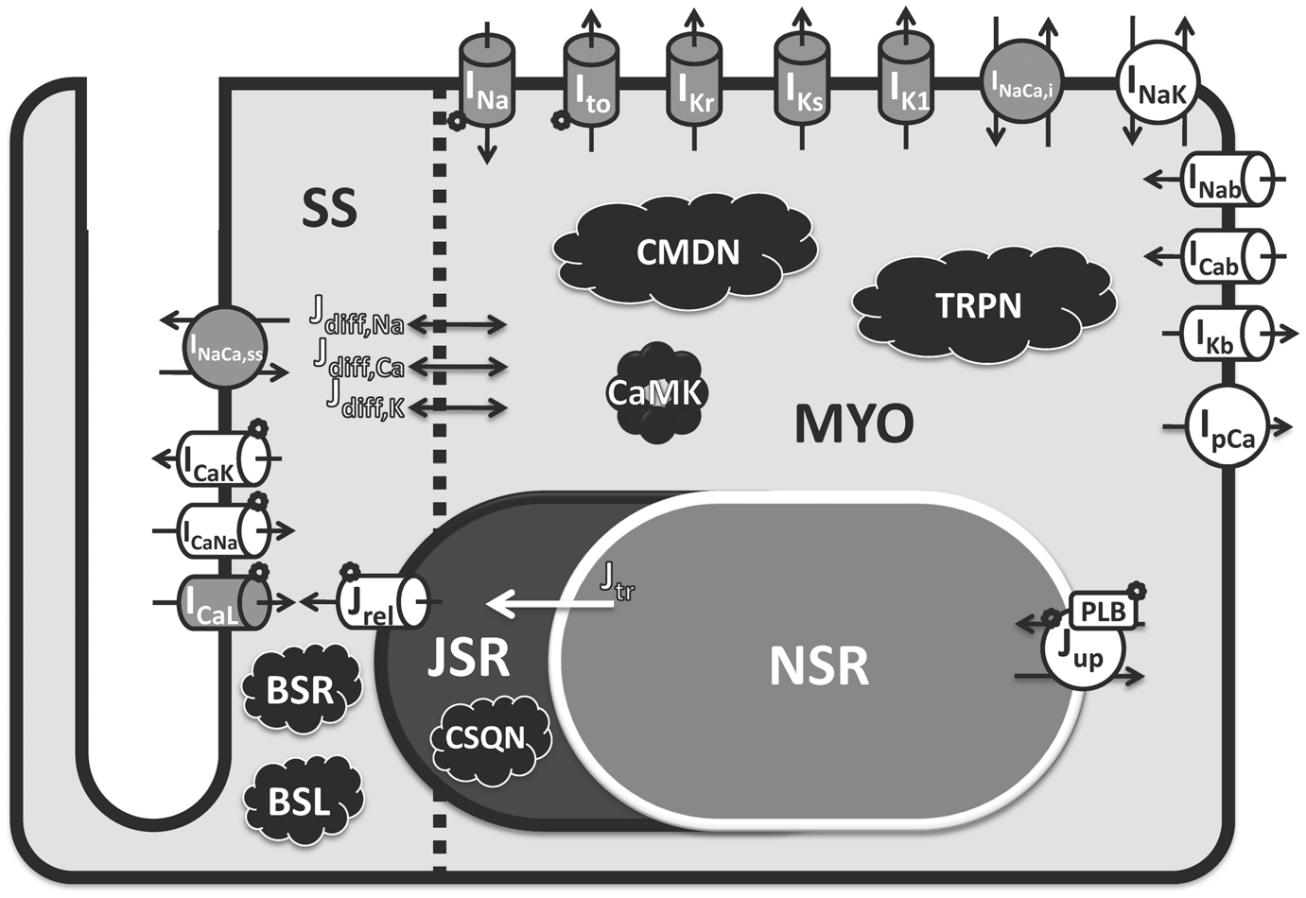
Schematic diagram of human ventricular myocyte model. Formulations for all currents and fluxes were based either directly (gray) or indirectly (white) on undiseased or nonfailing human experimental data. Model includes four compartments: 1) bulk myoplasm (myo), 2) junctional sarcoplasmic reticulum (JSR), 3) network sarcoplasmic reticulum (NSR), and 4) subspace (SS), representing the space near the T-tubules. Currents into the myoplasm: Na^+^ current (I_Na_; representing both fast and late components), transient outward K^+^ current (I_to_), rapid delayed rectifier K^+^ current (I_Kr_), slow delayed rectifier K^+^ current (I_Ks_), inward rectifier K^+^ current (I_K1_), 80% of Na^+^/Ca^2+^ exchange current (I_NaCa,i_), Na^+^/K^+^ pump current (I_NaK_), background currents (I_Nab_, I_Cab_, and I_Kb_), and sarcolemmal Ca^2+^ pump current (I_pCa_). Currents into subspace: L-type Ca^2+^ current (I_CaL_, with Na^+^ and K^+^ components I_CaNa_, I_CaK_), and 20% of Na^+^/Ca^2+^ exchange current (I_NaCa,ss_). Ionic fluxes: Ca^2+^ through ryanodine receptor (J_rel_), NSR to JSR Ca^2+^ translocation (J_tr_), Ca^2+^ uptake into NSR via SERCA2a/PLB (J_up_; PLB - phospholamban), diffusion fluxes from subspace to myoplasm (J_diff,Na_, J_diff,Ca_, and J_diff,K_). Ca^2+^ Buffers: calmodulin (CMDN), troponin (TRPN), calsequestrin (CSQN), anionic SR binding sites for Ca^2+^ (BSR), anionic sarcolemmal binding sites for Ca^2+^ (BSL). Ca^2+^/calmodulin-dependent protein kinase II (CaMK) and its targets are labeled.

Figure 5 shows a schematic diagram of the human ventricular AP model. The scheme was largely unchanged from the recent dog ventricular model by Decker et al.[19]. However, additional targets for CaMK were included, as described above, based on new findings. Currents were reformulated based on new undiseased or published nonfailing human experiments. These are colored gray in Figure 5. Currents and fluxes colored white in the figure were based on human specific measurements of rate dependence of intracellular Na^+^ and Ca^2+^ concentrations ([Na^+^]_i_ and [Ca^2+^]_i_, respectively), which these currents/fluxes affect. Equations for currents and fluxes were not adopted from other human or animal models without substantive modification; all equations were reformulated with the exceptions of Ca^2+^ buffers, CaMK kinetics, and background currents, for which we used Decker et al.[19] formulations and adjusted conductances. Model equations for all major currents were completely reformulated (i.e. fast I_Na_, late I_Na_, I_to_, I_CaL_, I_Kr_, I_Ks_, I_K1_, I_NaCa_, and I_NaK_). Relevant details precede equations in Supplement Text S1.

**Figure 6:**
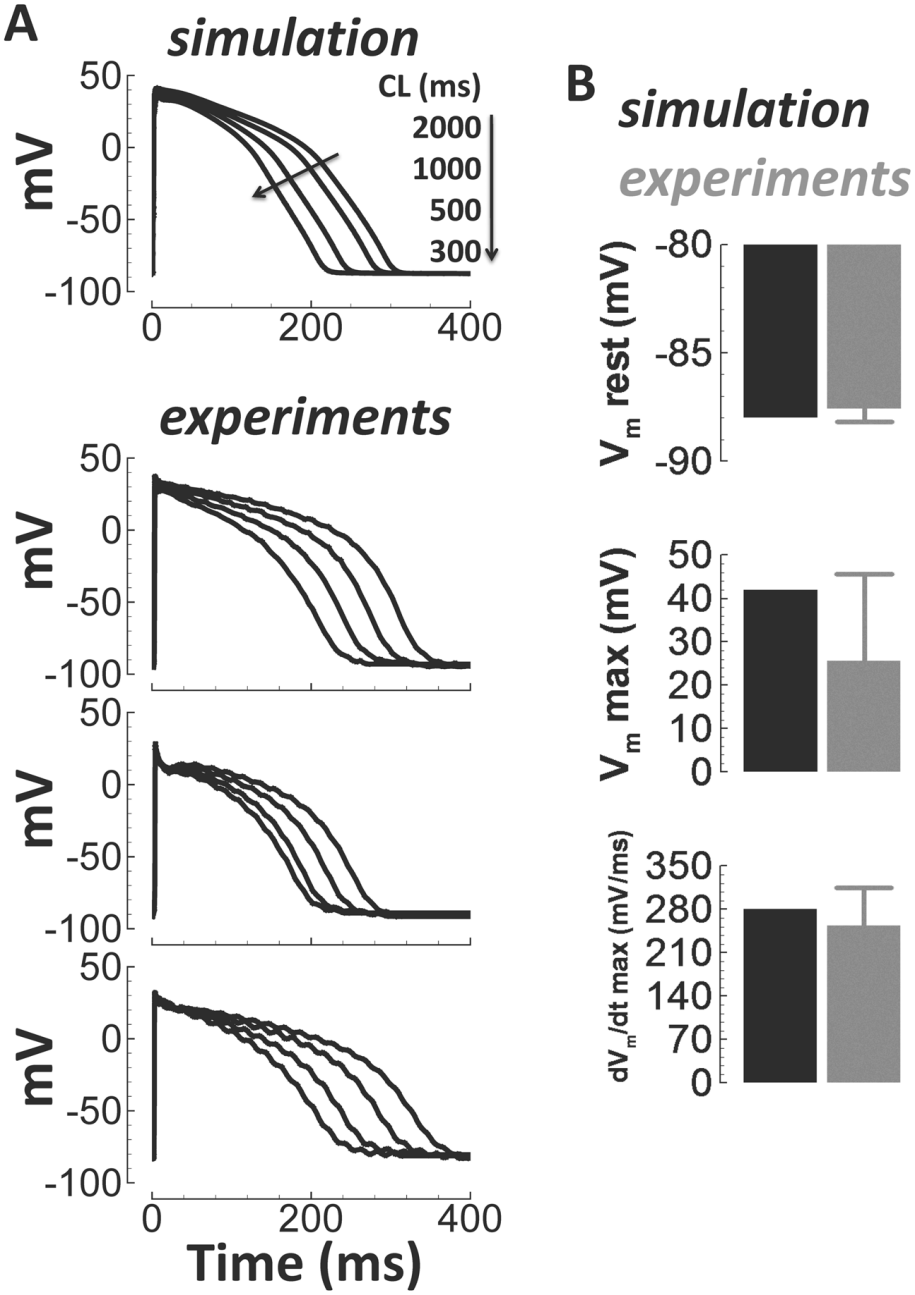
Undiseased human endocardial AP traces from experiments (small tissue preparations) and model simulations. Simulated APs for a range of pacing frequencies (top) and corresponding examples of experimentally recorded APs at 37°C (below). Arrows indicate cycle length (CL) changes. B) Comparison of simulation (black) and experimentally measured (gray, small tissue preparations) basic AP parameters for a single paced beat from quiescence (37°C, N = 32 from 32 hearts). Shown, from top to bottom, are the resting membrane potential (V_m_ rest), maximum upstroke potential (V_m_ max), and maximum upstroke velocity (dV_m_/dt max).

Microelectrode AP recordings from undiseased human ventricular endocardium at 37°C were used to validate basic human model AP characteristics. Figure 6A shows simulated APs and experimentally measured example APs for comparison during steady state pacing at the cycle lengths (CLs) indicated. We also compared simulated values for resting voltage, maximum voltage, and the maximum upstroke velocity, dV_m_/dt, with experiments (Figure 6B). These comparisons were made for a single beat, stimulated from the quiescent state.

**Figure 7:**
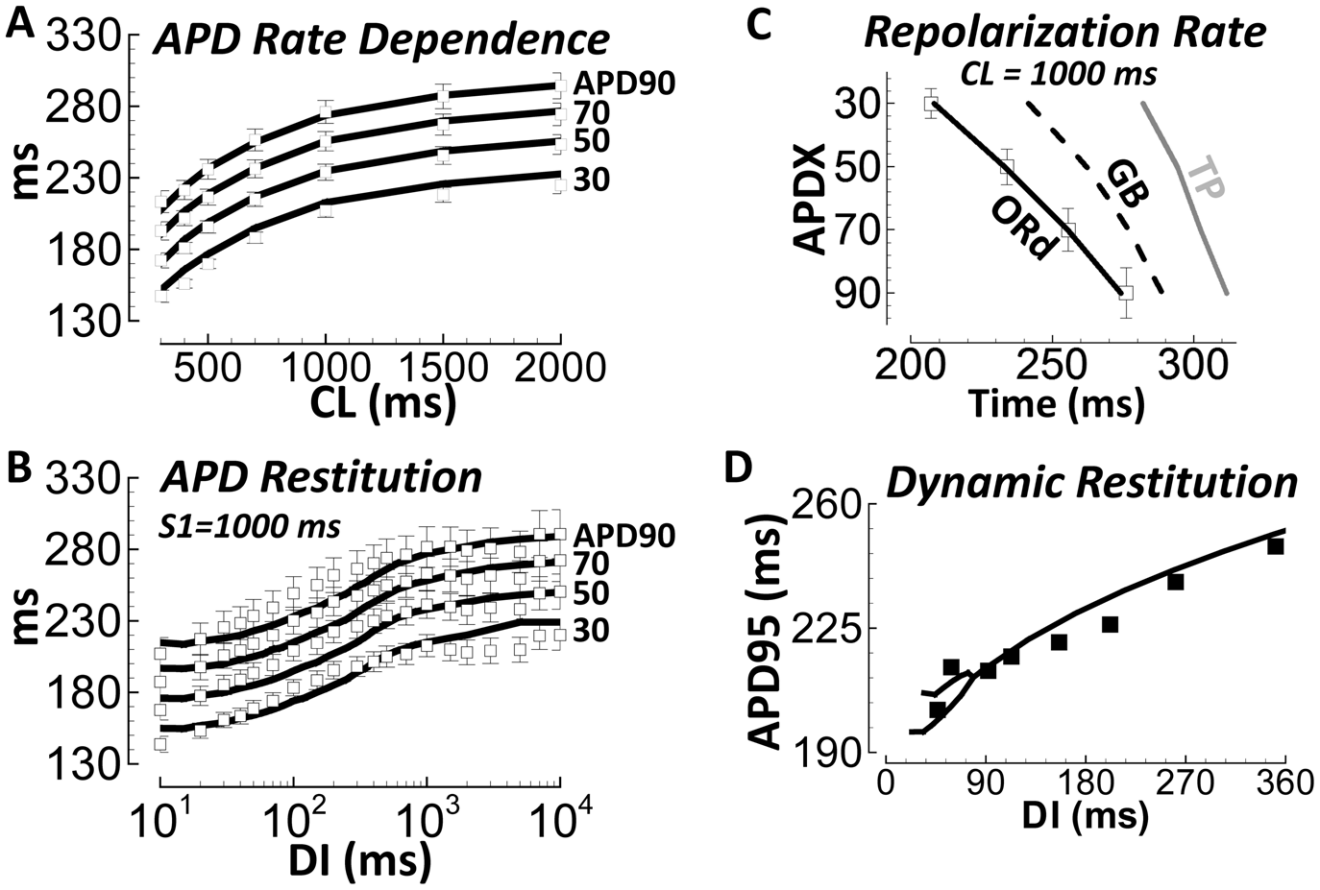
Undiseased human endocardial AP response to pacing protocols from experiments (small tissue preparations) and model simulations. A) Steady state APD rate dependence. B) S1S2 APD restitution (DI – diastolic interval). APD30–90 are labeled at right. Solid lines are simulation results; white squares are experiments at 37°C (N = 140 hearts in panel A, N = 50 hearts in panel B). C) Repolarization rate at CL = 1000 ms. Trajectory of APD30 to APD90 is accurate in the ORd model (white squares are experimental data); less so in other models. D) Dynamic restitution protocol (see Methods). Experiments are from Koller et al.[58], measured in nonfailing human hearts with monophasic AP electrodes (black squares). Simulated results are the black line. At very short diastolic intervals (DI<90 ms), the model shows APD bifurcation (alternans).

For steady state rate dependence, we compared APD30–90 after pacing at different CLs (Figure 7A). For restitution, we compared APD30–90 after steady state S1 pacing at CL = 1000 ms, followed by a single S2 extrasystolic stimulus delivered at various diastolic intervals (DIs, measured relative to APD90, Figure 7B). Model AP repolarization from 30 to 90% quantitatively reproduced this extensive dataset (simulation results were within experimental error bars). Generally, electrotonic effects of tissue coupling were minor (see Discussion and Supplement Figure S8 in Text S1).

The rate of repolarization in the model was gradual, as in experiments (APD30–90 were well separated in time, Figure 7C). Other models repolarized more rapidly and late compared to these experiments (simulations were all endocardial cell types).

Koller et al.[58] measured dynamic restitution in the nonfailing human ventricle with monophasic AP electrodes. Following the Koller protocol (explained in Methods), the human model matched Koller results (Figure 7D). Simulations predict a bifurcation (alternans) at shortest DIs (<90 ms), which is also observed in the experiments.

**Figure 8:**
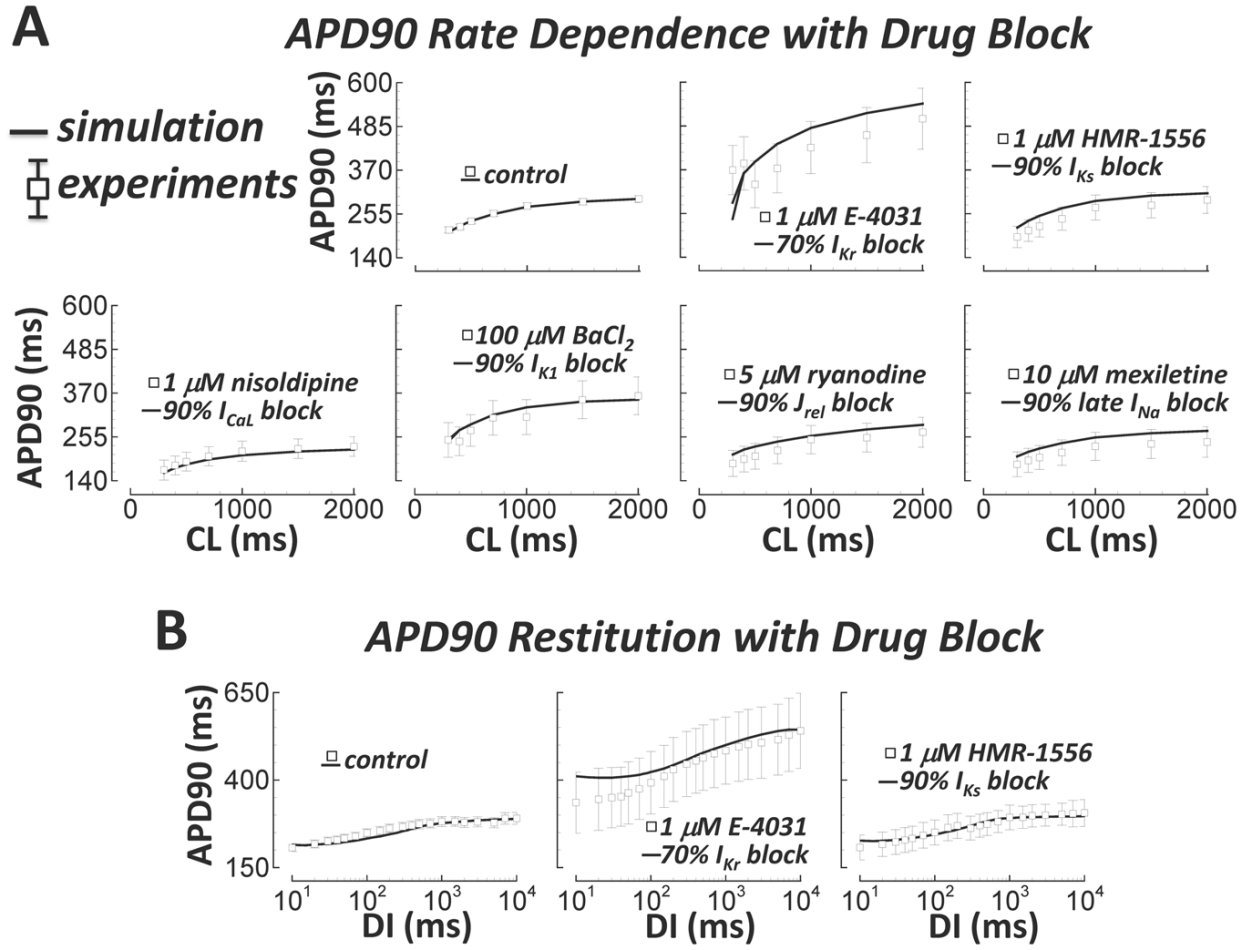
Pacing protocols with block of various currents. Experimental data (small tissue preparations) are white squares. A) Steady state APD90 rate dependence. From left to right, top to bottom: N = 140, 5, 5, 5, 5, 4, and 4 hearts. Shown are control, I_Kr_, I_Ks_, I_CaL_, I_K1_, RyR, and late I_Na_ block. B) APD90 restitution (S1 = 1000 ms). From left to right: N = 50, 3, and 4 hearts. Shown are control, I_Kr_, and I_Ks_ block.

Steady state rate dependence and restitution of the undiseased human ventricular APD were also measured in the presence of channel-specific blockers (Figure 8, white squares, see Methods for further details). In Figure 8, drugs and applied doses are provided for each experiment. Simulated block was based on experimental dose-response measurements (E-4031[59], HMR-1556[60], nisoldipine[61], BaCl_2_ [62], ryanodine[63], and mexiletine[64], for block of I_Kr_, I_Ks_, I_CaL_, I_K1_, J_rel_, and late I_Na_, respectively). Simulations matched these experiments; that is, simulation results were within experimental error bars.

**Figure 9:**
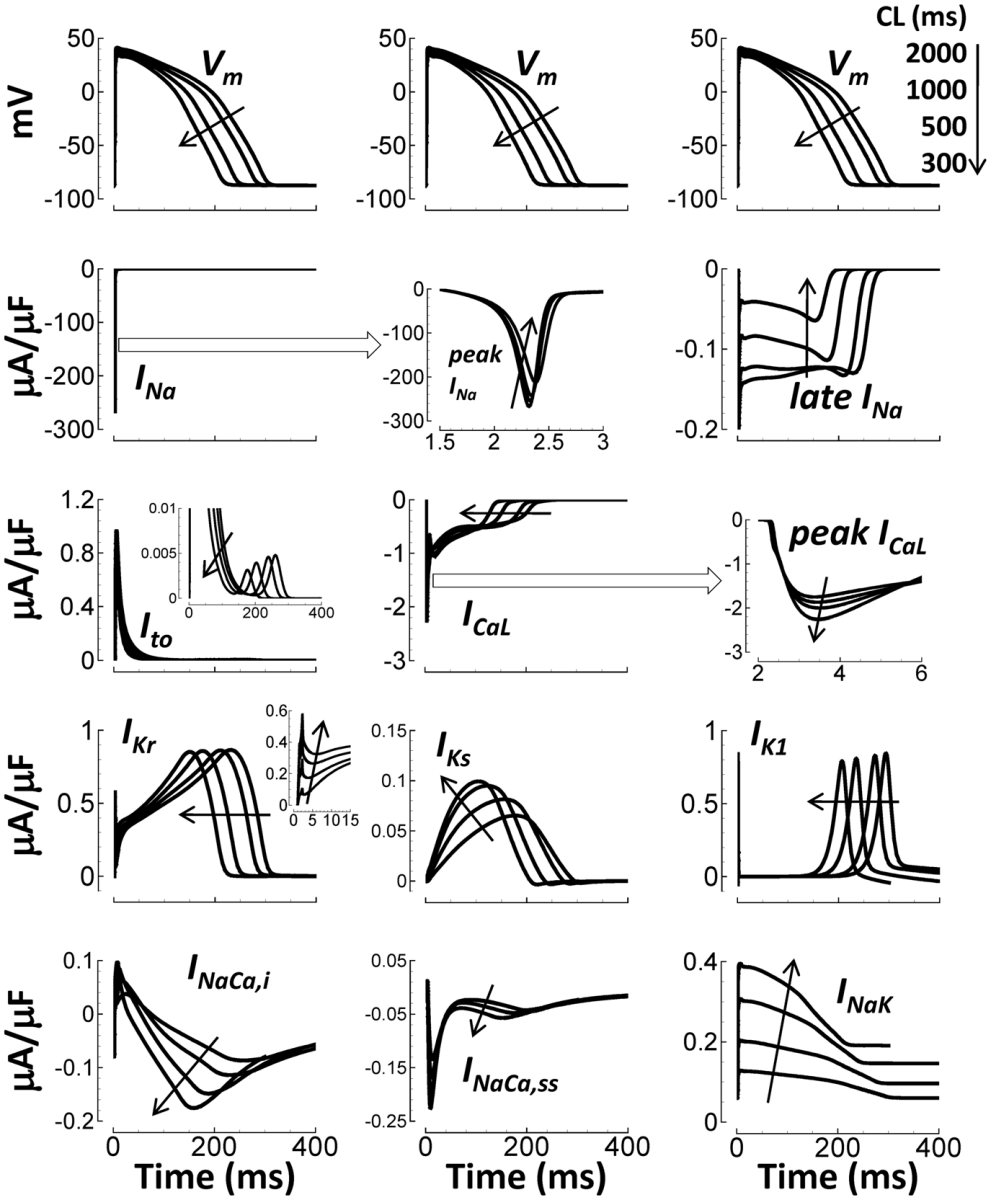
Rate dependence of currents at steady state. Black arrows indicate CL decrease (rate increase). Top Row) Simulated APs, repeated in each column for timing purposes. Lower Rows (left to right, top to bottom): I_Na_, peak I_Na_ detailed time course, late I_Na_, I_to_, I_CaL_, I_CaL_ increasing peaks with increasing pacing rate, I_Kr_, I_Ks_, I_K1_, I_NaCa,i_, I_NaCa,ss_, and I_NaK_. Insets show greater detail of late small I_to_ window current, and early I_Kr_ spiking at fast rates.

As pacing CL was decreased from 2000 to 300 ms, currents in the human ventricular AP model changed accordingly (Figure 9). Due to increased refractoriness at faster rates, maximum fast I_Na_, late I_Na_, and I_to_ were reduced. By contrast, peak I_CaL_ increased, due in part to CaMK-phosphorylation induced facilitation[65]. I_Kr_ and I_K1_ were largely rate independent. Mild I_Ks_ accumulation[66] caused rate dependent increase in current. I_NaK_ became larger due to intracellular Na^+^ accumulation at fast pacing rates (details below). I_NaCa,i_, and I_NaCa,ss_ became more inward, in order to remove increasing Ca^2+^.

### Transmural Heterogeneity

**Figure 10:**
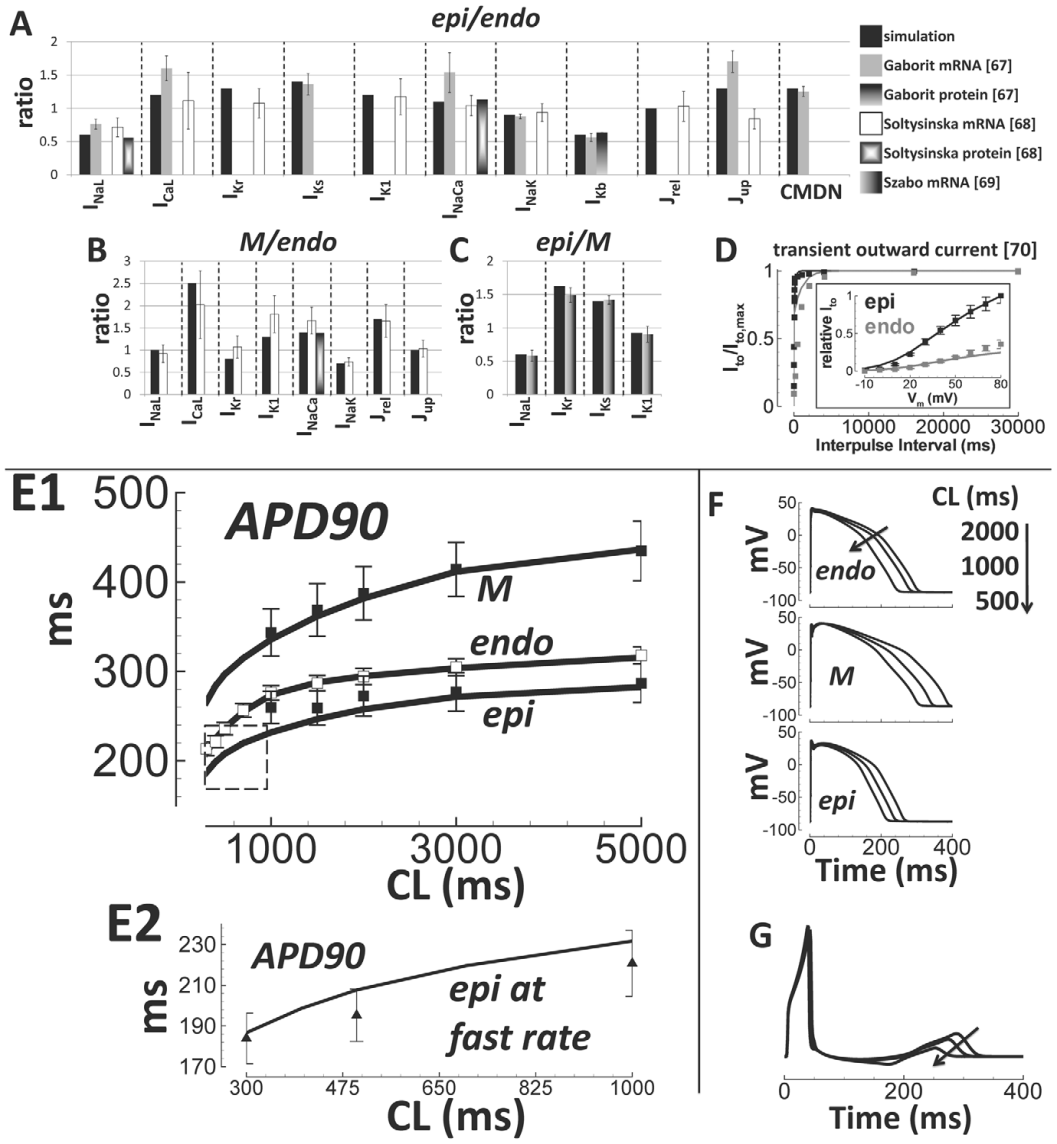
Transmural heterogeneity and validation of transmural cell type models. A–C) Expression ratio in the model (black bars) compared to experimental data from undiseased human hearts (grayscale bars, labeled). D) Transmural heterogeneity of I_to_; simulations are lines, experiments are squares. Results for endo are gray; those for epi are black. E1) Rate dependence of APD90 in endo, M, and epi cell types. Epi and M data were obtained by scaling endo data (white squares) by epi/endo and M/endo APD90 ratios from Drouin et al.[50] (black squares). Simulations are black lines. E2) Same format as panel E1, showing epi APD90 at faster pacing rates. Data are from Glukhov et al.[71], (epi/endo scaling, black triangles). F) Top to bottom: Rate-dependence of endo, M, and epi APs. G) Pseudo-ECG, using a simulated transmural wedge. CL changes are indicated by arrows.

Changes in mRNA and protein expression across the transmural wall using undiseased human ventricles were measured[67], [68], [69]. Functional data for transmural changes in I_to_ were measured in nonfailing human ventricular myocytes[70]. These results were compiled to create a complete dataset for transmural differences between endocardial (endo), mid-myocardial (M), and epicardial (epi) cell types. We considered transmural differences in Nav1.5, Cav1.2, HERG1, KvLQT1, Kir2.1, NCX1, Na/K ATPase, Kv1.5, RyR2, SERCA2, and CALM3 to be represented in the model by late I_Na_, I_CaL_, I_Kr_, I_Ks_, I_K1_, I_NaCa_, I_NaK_, I_Kb_, J_rel_, J_up_, and CMDN, respectively. Whenever an expression ratio was not available, we chose unity. Using this analysis, models for M and epi cells were derived from the thoroughly validated endo model (Figure 10A–10D; equations on page 19 in Supplement Text S1).

In Figure 10E1, our experimental measurements for endo APD90 were scaled by M/endo and epi/endo APD90 ratios measured by Drouin et al.[50] and compared to simulations. Drouin experiments did not show results for CL<1000 ms. Epi simulations seem to deviate from Drouin experiments at faster pacing rates. However, epi simulations were consistent with nonfailing human epi experimental measurements at fast pacing rates (CL <1000 ms) recorded using optical mapping by Glukhov et al.[71] (panel E2). The rate dependence of simulated AP morphology in the different cell types (Figure 10F) was similar to Drouin recordings[50]. Relative shape and duration of simulated transmural APs were also consistent with those recorded by Glukhov et al.[71] from the heart of a 20 year old healthy human male (Supplement Figure S9 in Text S1). The transmural repolarization gradient direction was such that the pseudo-ECG T-wave was upright and rate dependent[72] as expected (Figure 10G).

### Early Afterdepolarization (EAD)

**Figure 11:**
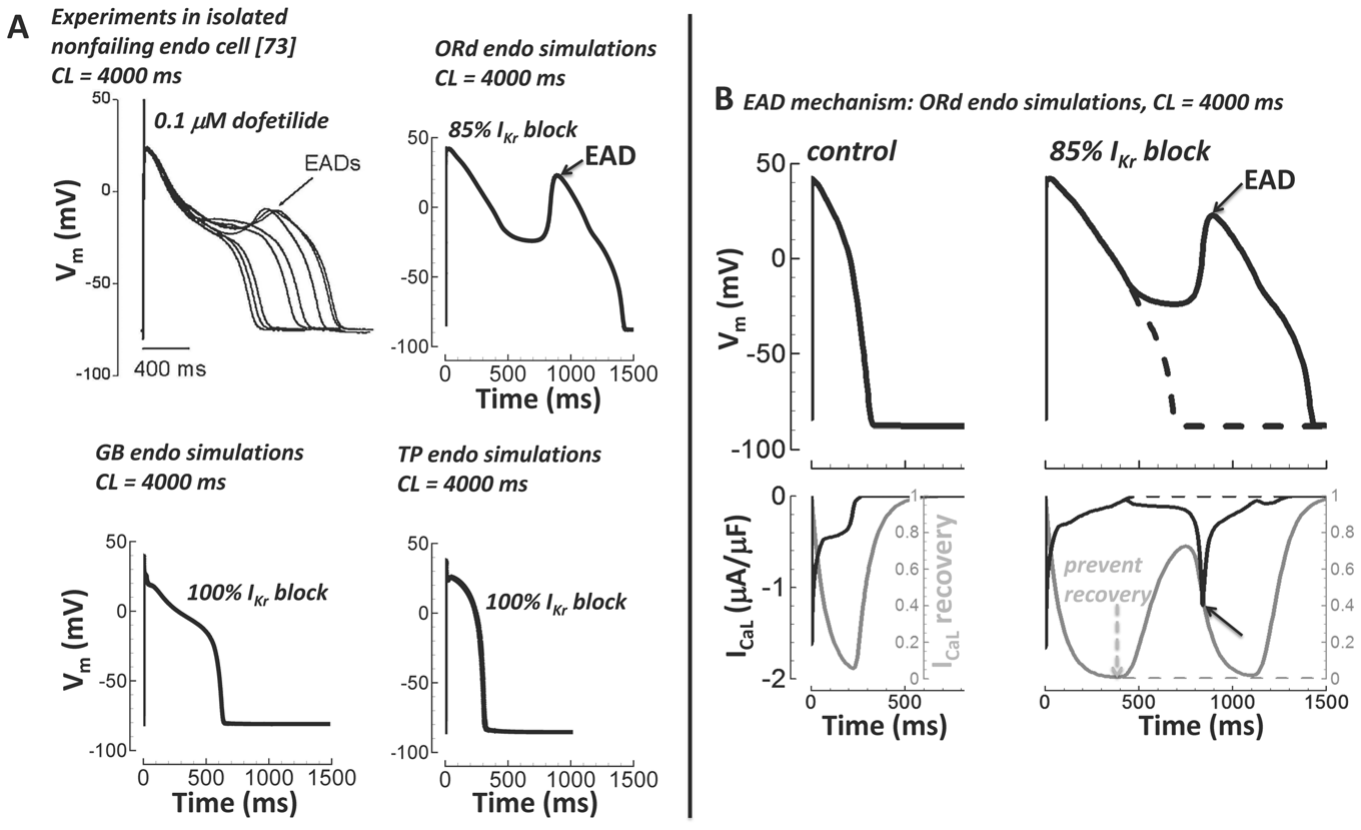
Early afterdepolarizations (EADs). A) Top left) Experiments in isolated nonfailing human endo myocytes from Guo et al.[73] showed EADs with slow pacing (CL = 4000 ms) in the presence of I_Kr_ block (0.1 µM dofetilide, ∼85% I_Kr_ block[74], reproduce with permission). Top right) Following the experimental protocol of Guo et al. (CL = 4000 ms, 85% I_Kr_ block) the ORd model accurately showed a single large EAD. Bottom) GB (left) and TP (right) models failed to generate EADs (CL = 4000 ms, even with 100% I_Kr_ block). B) EAD mechanism. APs are on top. I_CaL_ (black) and I_CaL_ recovery gate (gray) are below. Slow pacing alone (CL = 4000 ms) did not cause an EAD (left). Slow pacing plus I_Kr_ block (85%) caused an EAD (solid lines, right). The EAD was depolarized by I_CaL_ reactivation during the slowly repolarizing AP plateau (solid lines, solid arrows). When I_CaL_ recovery was prevented, the EAD was eliminated (dashed lines and dashed arrow).

Experiments from Guo et al.[73] in isolated nonfailing human ventricular endo myocytes showed EADs when paced very slowly (CL = 4000 ms) in the presence of the I_Kr_ blocker dofetilide (0.1 µM dose, ∼85% I_Kr_ block[74]). In Figure 11A, we display Guo experimental results and simulation results of the same protocol using the ORd model, and the GB and TP models (all for endo cells at steady state). As in the experiment, the ORd model produced an EAD when paced at slow rate (CL = 4000 ms) with block of I_Kr_ (85%). Experiments and simulations both show a single, large EAD deflection. The GB and TP models failed to produce an EAD following the same protocol (CL = 4000 ms), even with complete block of I_Kr_ (100%).

EADs in the ORd model were caused by I_Kr_ block induced prolongation of the time at plateau voltages, allowing I_CaL_ reactivation. When I_CaL_ recovery was prevented, the EAD was eliminated (inactivation gate clamping protocol, Figure 11B). This mechanism is the same as shown previously in other species[75].

### Na^+^ and Ca^2+^ Rate Dependence

**Figure 12:**
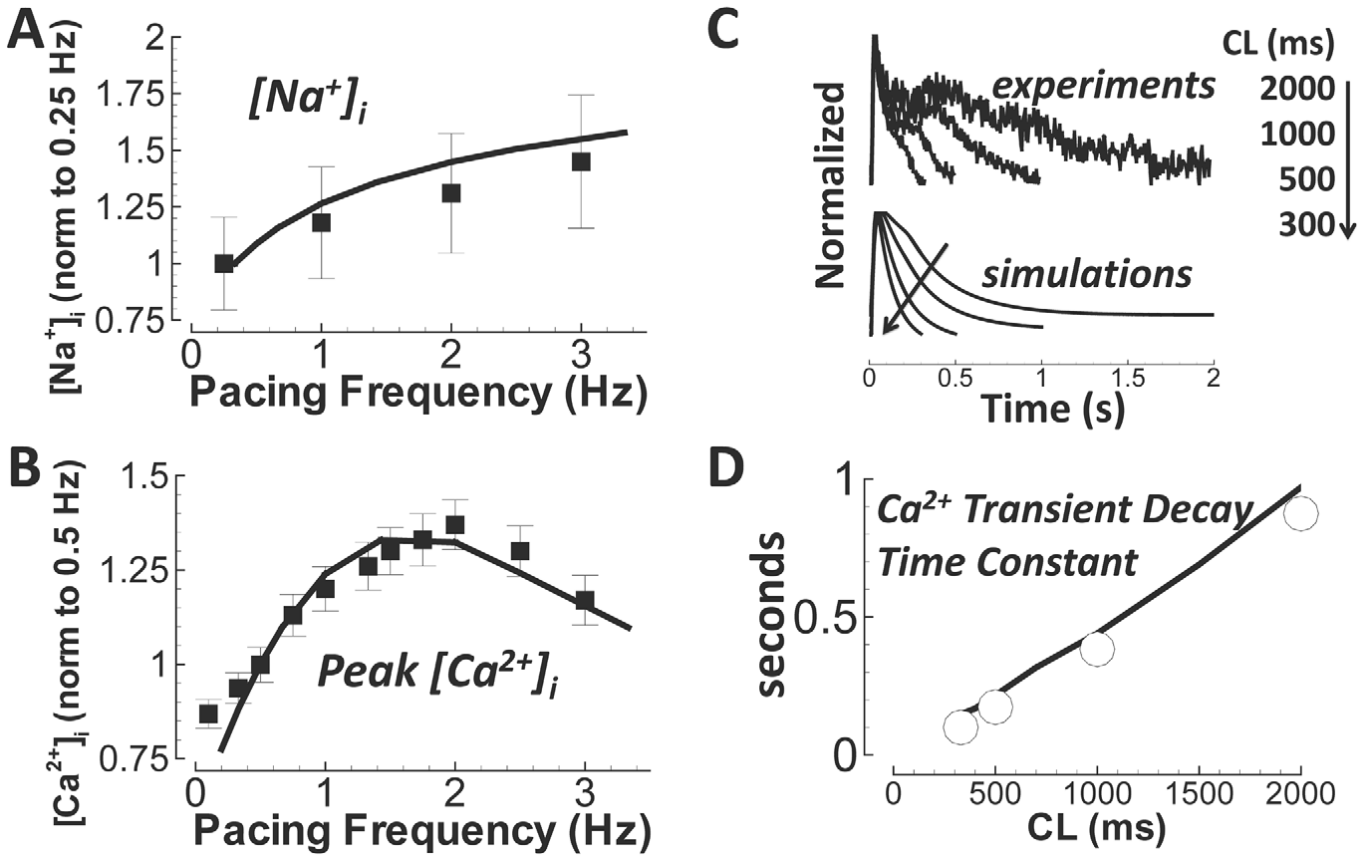
Rate dependence of intracellular ion concentrations. Simulation results are solid lines. A) [Na^+^]_i_ versus pacing frequency (normalized to 0.25 Hz). Experiments are from nonfailing myocytes (Pieske et al.[57], black squares). B) Peak Ca^2+^ transient (normalized to 0.5 Hz). Experiments are from nonfailing myocytes (Schmidt et al.[76], black squares). C) Ca^2+^ transients from experiments (Fura-2-AM) and simulations. Results are normalized to illustrate the time course of decay. The arrow indicates pacing CL changes. D) Frequency dependent acceleration of relaxation. Undiseased human experimental data are white circles. Simulations are the black line.

**Figure 13:**
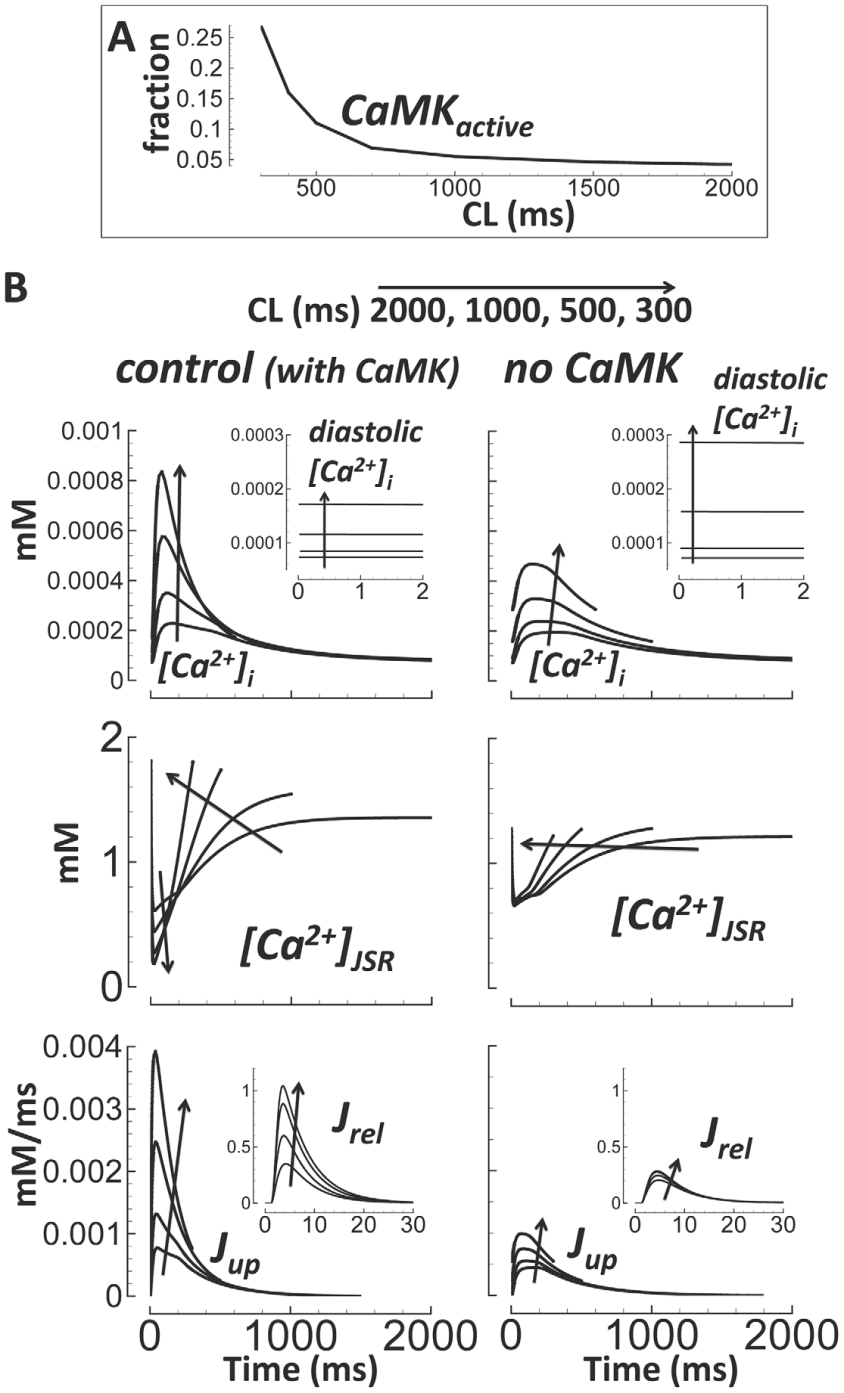
CaMK and Ca^2+^ cycling. A) Rate dependence of CaMK active fraction. B) Ca^2+^ cycling under control conditions (left) and without CaMK (right). CL changes are indicated by arrows. Top) [Ca^2+^]_i_ and diastolic values (inset). Middle) [Ca^2+^]_JSR_. Bottom) J_up_ and J_rel_ (inset, expanded time scale).

Using data from nonfailing human ventricle, we validated rate dependent changes in concentrations of intracellular Na^+^ and Ca^2+^. For [Na^+^]_i_ changes with pacing rate, we used data from Pieske et al.[57], measured in the nonfailing human ventricle, normalized to 0.25 Hz pacing rate (Figure 12A). Reproduction of this curve implied that I_NaK_ magnitude was accurate (I_NaK_ conductance controls intracellular Na^+^, thus rate dependence of relative accumulation, Supplement Figure S18 in Text S1). For Ca^2+^, we used data from Schmidt et al.[76], normalized to the value at 0.5 Hz pacing rate. A personal correspondence with senior author J. Gwathmey revealed that pacing in the experiments was for about 100 beats (long enough to reach apparent steady state). Following this protocol, we showed the reduction in peak Ca^2+^ observed at the fastest pacing rates (Figure 12B). However, at true steady state, peak Ca^2+^ increased monotonically with pacing rate (shown in Figure 13).

Using Fura-2-AM fluorescence data measured in an undiseased isolated human ventricular myocyte at 37°C, we determined that the ORd model showed accurate intracellular Ca^2+^ decay (Figure 12C and 12D). Time constant fits were a single exponential decay from time of peak Ca^2+^. The decrease in decay time constant observed with increase in pacing rate is a measure of frequency dependent acceleration of relaxation, an importa

nt validation of Ca^2+^ cycling.

### Ca^2+^ Cycling and CaMK

As pacing rate increased, so did the CaMK active fraction (CaMK_active_, Figure 13A, validated previously[31], [77]). CaMK was important for controlling rate dependence of Ca^2+^ cycling in the model. In the absence of CaMK: Ca^2+^ transient amplitude was reduced, diastolic Ca^2+^ was elevated, JSR Ca^2+^ content and evacuation were rate independent, and Ca^2+^ reuptake (J_up_) and release (J_rel_) were severely blunted (Figure 13B).

### Alternans

**Figure 14:**
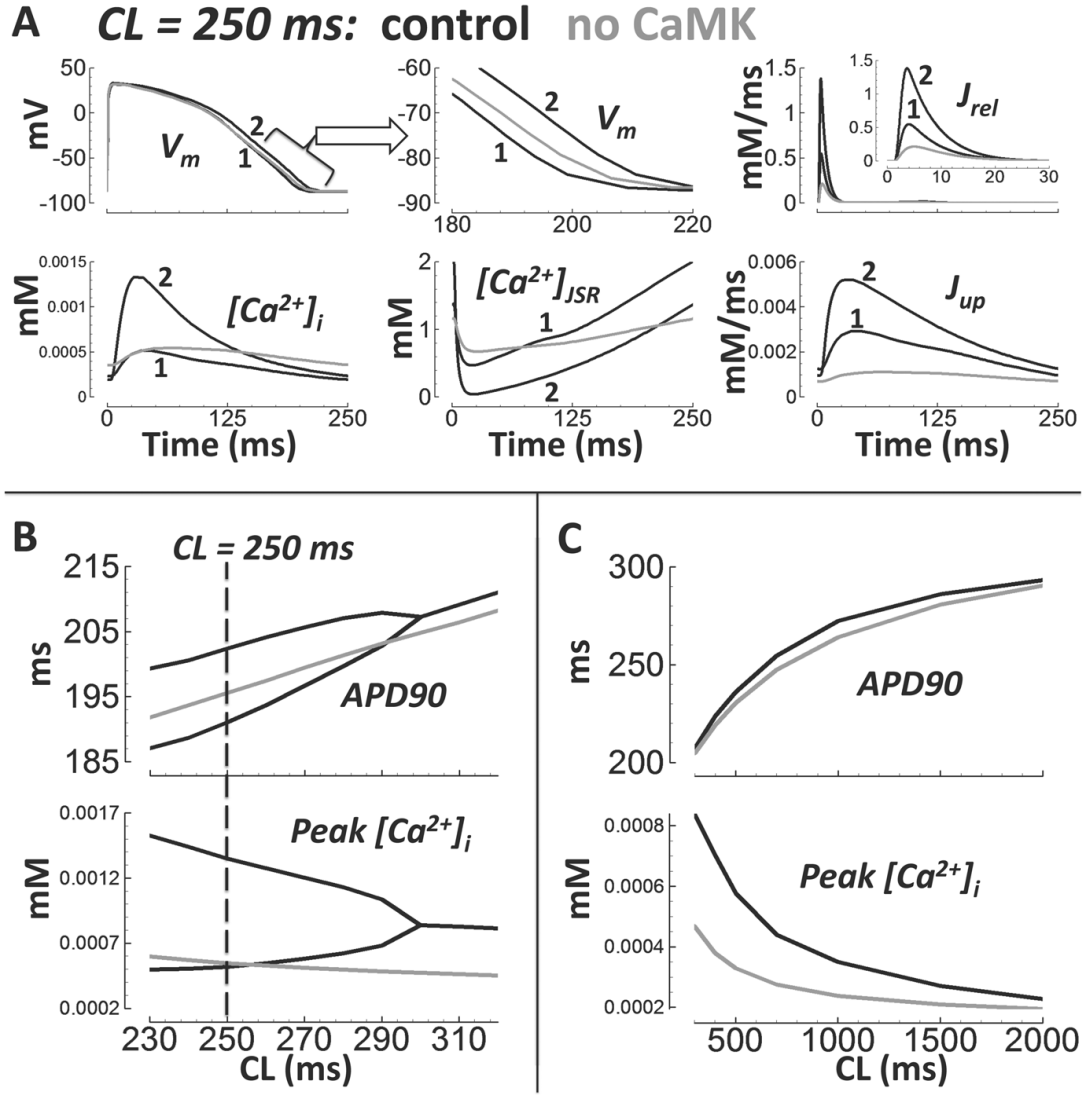
AP and Ca^2+^ alternans at fast pacing. Black lines are control. Gray lines are without CaMK. The two consecutive beats are labeled 1 and 2. A) Pacing at CL = 250 ms. From left to right, top to bottom: AP, expanded time scale showing AP repolarization, J_rel_ (inset is expanded time scale), [Ca^2+^]_i_, [Ca^2+^]_JSR_, and J_up_. B) Rate dependence of APD (top) and peak [Ca^2+^]_i_ (bottom) at fast rates (alternans bifurcations disappear without CaMK). C) Same as panel B, but at slower rates (no bifurcations).

Koller et al.[58] showed that in the nonfailing human ventricle (*in vivo* monophasic AP recordings), APD alternans appeared at CLs <300 ms (rates >200 bpm). The amplitude of APD alternans was ∼10 ms. These findings were reproduced by the human model (APD alternans of 11 ms at CL = 250 ms, Figure 14). Pacing at rates faster than 230 ms in the model caused 2 to 1 block (i.e. failed APs every other beat), because APD began to encroach upon the pacing cycle length, leading to enhanced refractoriness of Na^+^ current due to incomplete repolarization.

Since Koller measurements were performed in intact hearts, electrotonic coupling effects would have played a role. Therefore, simulations in a strand of 100 coupled endo cells were conducted to test whether alternans occurred in coupled tissue as well. Indeed, during CL = 280 ms steady state pacing, alternans developed in the multicellular fiber (results shown in Supplement Figure S10 in Text S1).

As in Livshitz et al.[77], beat to beat alternans in the Ca^2+^ subsystem were the cause of the APD alternans in the model. Longer APs coincided with larger Ca^2+^ transients. For steady state pacing at 250 ms pacing cycle length (shown in Figure 14A), we found that clamping the subspace Ca^2+^ concentration to either the odd or even beat waveforms eliminated alternans, but clamping of the voltage, myoplasmic Ca^2+^, I_CaL_, or I_NaCa_ did not eliminate alternans (odd or even beat clamp, not shown).

Cutler et al.[78] demonstrated that 30% SERCA upregulation eliminated alternans. Similarly, in our human model, a 20% increase in J_up_ magnitude eliminated alternans (shown in Supplement Figure S11 in Text S1). CaMK suppression also eliminated alternans in the model (Figure 14A and 14B, gray traces). At slower pacing rates, APD was minimally affected by CaMK suppression. However, the peak Ca^2+^ concentration was markedly reduced, especially at faster rates (Figure 14C).

### Currents Participating in Steady State APD Rate Dependence and APD Restitution

**Figure 15:**
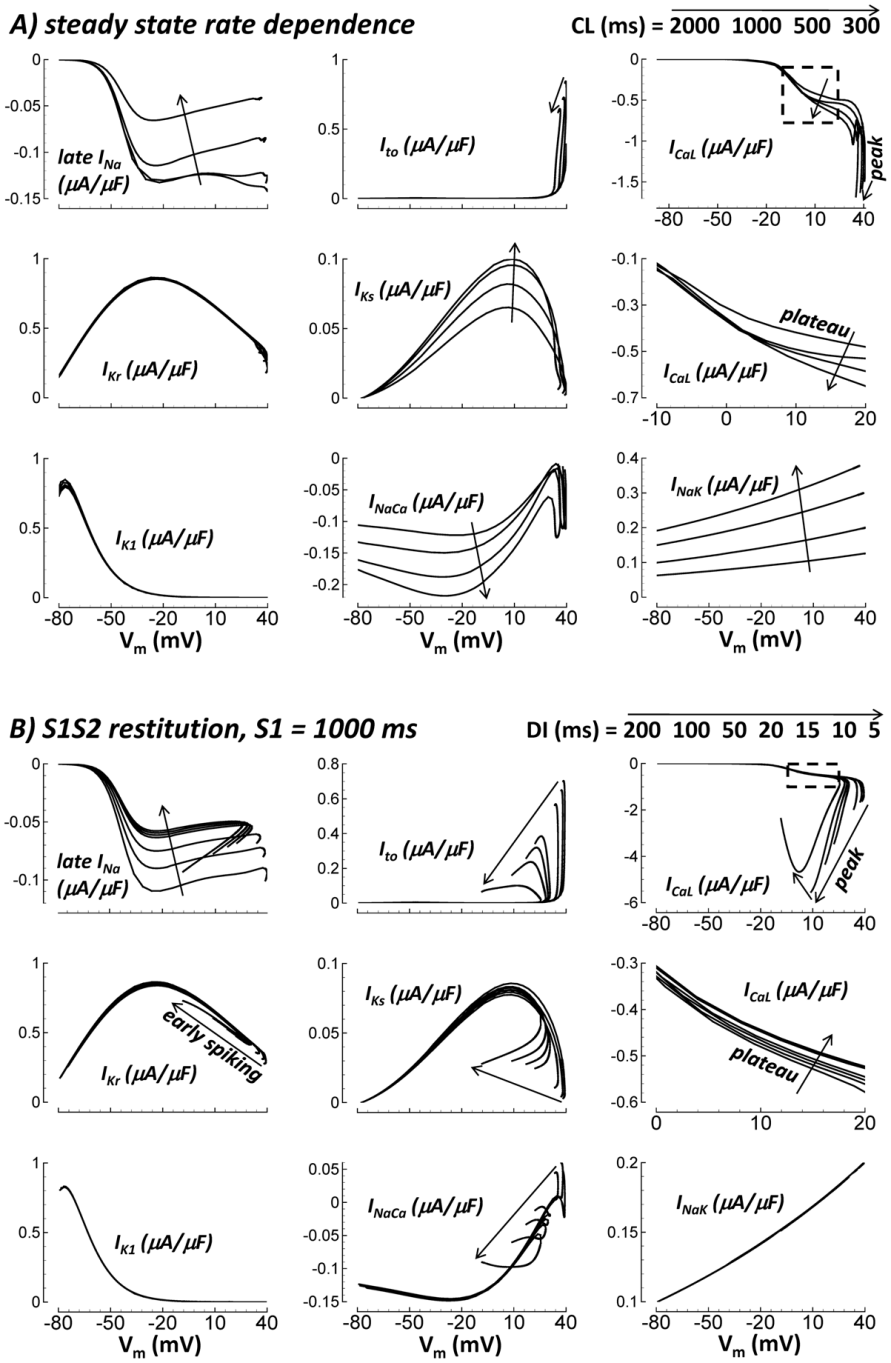
I–V curves during steady state rate dependent pacing at various CLs (panel A), and S1S2 restitution at various DIs (panel B). Arrows indicate decreasing CL or DI. From left to right, top to bottom, results for late I_Na_, I_to_, I_CaL_, I_Kr_, I_Ks_, zoom of plateau I_CaL_ (dashed box section), I_K1_, I_NaCa_, and I_NaK_ are shown.

In order to describe the mechanisms underlying steady state rate dependence and restitution of the APD in the model, it is instructive to first systematically determine which currents participate in these phenomena. In Figure 15, currents were plotted versus V_m_ during steady state and S1S2 restitution pacing for a variety of CLs and DIs, respectively. If I–V curves are CL or DI independent (i.e. curves overlap), then that current did not participate in steady state rate dependence or restitution, respectively. Conversely, if I–V curves depended greatly on CL or DI, then that current played at least some role in these phenomena.

As CL or DI decreased, fast I_Na_, responsible for the maximum AP upstroke velocity and maximum V_m_, was reduced (see Figure 9, and principles detailed in Luo and Rudy[79]). This is because shortened time at resting potential between beats prevents complete recovery from inactivation. Thus, at fast pacing rates, and short DIs, maximum V_m_ and upstroke velocity were reduced, explaining some of what follows.

During steady state pacing, I_Ks_ was strongly rate dependent (Figure 15A). The I–V curves were dramatically different at different pacing CLs. However, I_Ks_ was a relatively small contributor to the rate dependence of APD because I_Ks_ density in human ventricle is small under basal conditions (no β-adrenergic stimulation), and changes relative to slow rate values produced minimal additional outward current at fast rates.

Late I_Na_, I_CaL_, I_NaCa_ and I_NaK_ also showed CL dependent changes during steady state pacing (Figure 15A). I_NaK_ became more outward at fast rates. The changes in I_NaK_ were dramatic, and the current density was relatively large. Thus, I_NaK_ was an important contributor to APD shortening at fast pacing rates. Late I_Na_ became dramatically less inward at fast rates, making it a secondary contributor to APD shortening at fast rates. Changes in I_CaL_ and I_NaCa_ opposed APD shortening at fast rates; these currents became more inward at short CLs. I_NaCa_ increased to match the increased Ca^2+^ extrusion burden. Importantly, I_CaL_ increased despite reduced channel availability. I_CaL_ inactivation gates recovered less between beats as pacing rate increased (∼20% less at CL = 300 ms compared to CL = 2000 ms). The same mechanism caused reduced late I_Na_ at fast rates (availability at CL = 300 ms was ∼1/3 that at CL = 2000 ms). However, influences of increased CaMK facilitation combined with increased driving force (reduced maximum V_m_) actually caused I_CaL_ to become larger at fast rates.

If Na^+^ is clamped to small values associated with slow pacing ([Na^+^]_i_ and [Na^+^]_ss_ = 6.2 mM at CL = 2000 ms), preventing its accumulation at fast rates, I_NaK_ remains small and CL independent (this mechanism is described later in detail), causing plateau voltages to become relatively CL independent. Thus, with Na^+^ clamp, I_CaL_ changes with pacing rate are different than under control conditions. CL independent plateau voltages confer CL independence to the driving force for plateau I_CaL_. Na^+^ clamping reduced Ca^2+^ (via I_NaCa_) which reduced activated CaMK and thus I_CaL_ facilitation. An interesting consequence is that with Na^+^ clamp, I_CaL_ changes with CL help to cause APD shortening at fast rates, whereas in control (i.e. no Na^+^ clamp), I_CaL_ changes with CL oppose APD shortening.

During restitution, late I_Na_, I_to_, I_CaL_, I_Ks_ and I_NaCa_ showed DI dependent changes (Figure 15B). Dramatically less inward late I_Na_ at short DIs helped shorten the APD. The mechanism was reduced availability due to residual inactivation at the start of the S2 beat. I_CaL_ was reduced for the same reason. This was evident during the plateau. CaMK facilitation did not depend on DI because Ca^2+^ accumulation (necessary for CaMK activation) is slow, occurring only after long term pacing to steady state. Similarly, Na^+^ did not accumulate at short DIs, which kept I_NaK_ constant. Therefore, plateau potentials and I_CaL_ driving force during the plateau were relatively DI independent. Just as in the case of Na^+^ clamp, these properties combined to allow reduced availability of I_CaL_ at short DI to dominate the behavior. However, reduced maximum V_m_ increased the driving force during the time of peak I_CaL_, which caused peak current to generally increase at short DIs. At extreme DI of 5 ms, the slow AP upstroke (i.e. reduced dV_m_/dt) caused mild I_CaL_ inactivation coincident with activation, so the peak current was reduced compared to DI = 10 ms.

Changes in other currents (I_to_, I_Ks_ and I_NaCa_), though nonzero, were relatively minor due to timing. DI dependent changes that increased or reduced current during phase-1 of the AP had little effect on final repolarization time. The exception is I_Kr_. I_Kr_ is large enough that early spiking helped shorten APD at very short DIs (detailed simulations follow).

### Ionic Basis for APD Rate Dependence and Restitution

**Figure 16:**
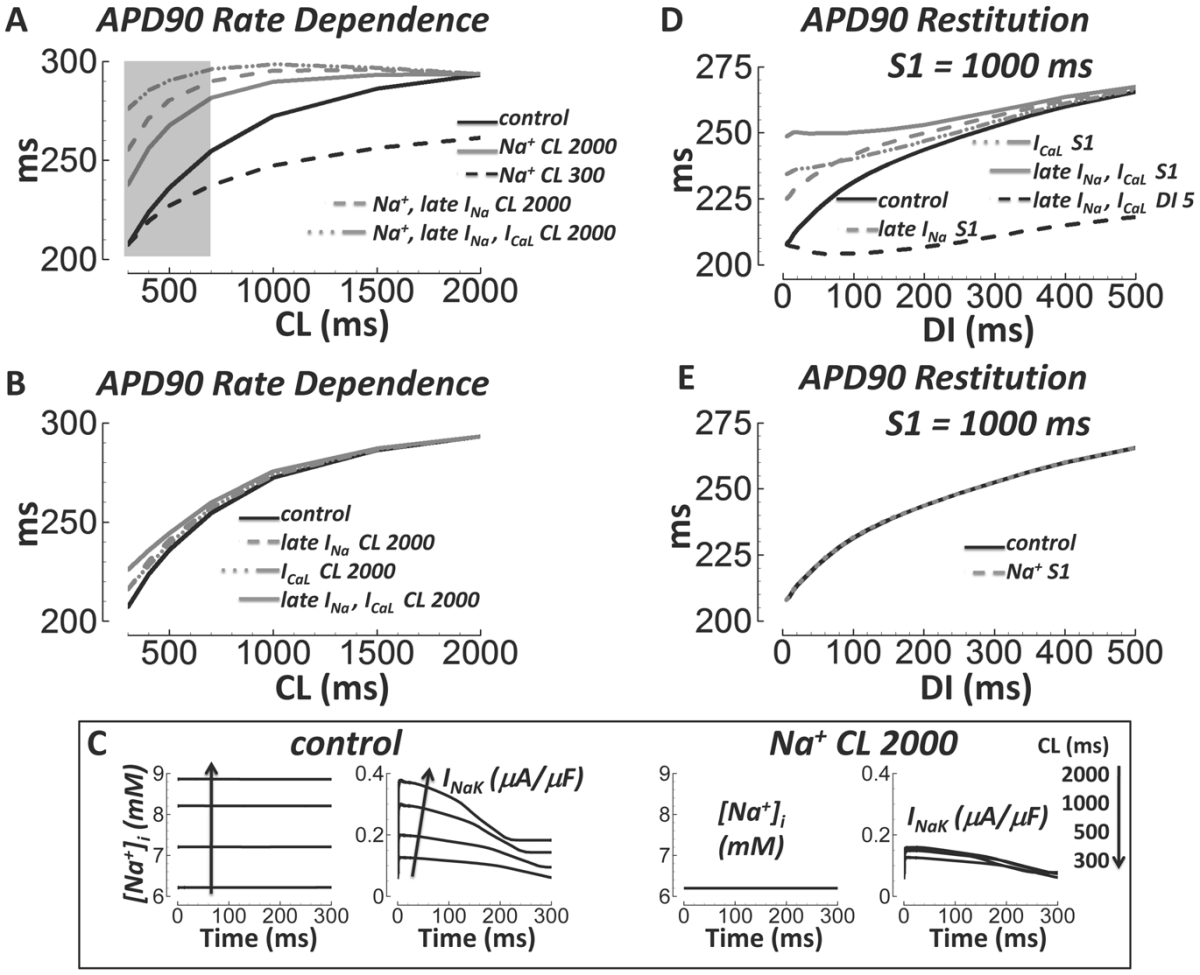
Major causes of steady state APD rate dependence and S1S2 APD restitution. A) APD rate dependence in control (solid black), and with [Na^+^]_i_ and [Na^+^]_ss_ clamped to slow rate (solid gray) or fast rate (dashed black) values. When late I_Na_ (dashed gray) or both late I_Na_ and I_CaL_ inactivation gates were reset to their slow rate values (dash-dot-dot gray) in addition to [Na^+^]_i_ and [Na^+^]_ss_ slow rate clamp, APD lost almost all rate dependence. Note that slow rate [Na^+^]_i_ and [Na^+^]_ss_ clamp alone left residual APD rate dependence, especially at fast rates (CL = 300 to 700 ms, shaded box). B) APD rate dependence (control, solid black) was largely unaffected by resetting inactivation gates for late I_Na_ (dashed gray), I_CaL_ (dash-dot-dot gray), or late I_Na_ and I_CaL_ (solid gray) to their slow rate values (no [Na^+^]_i_ and [Na^+^]_ss_ clamping to slow rate values). C) [Na^+^]_i_ and I_NaK_ increase with pacing rate under control conditions (left). When [Na^+^]_i_ and [Na^+^]_ss_ are clamped to slow rate values, I_NaK_ is small and rate independent (right). D) APD restitution in control (solid black), and when inactivation gates were reset to S1 values upon S2 delivery (late I_Na_ reset – dashed gray, I_CaL_ reset – dash-dot-dot gray, late I_Na_ and I_CaL_ reset – solid gray). Shown in dashed black is resetting late I_Na_ and I_CaL_ inactivation to the DI = 5 ms value. E) [Na^+^]_i_ and [Na^+^]_ss_ clamp to S1 values (dashed gray) did not affect APD restitution (control, solid black).

Steady state rate dependence of the APD was largely caused by accumulation of intracellular Na^+^ at fast rates. This is illustrated in Figure 16A. When [Na^+^]_i_ and [Na^+^]_ss_ were clamped to values from steady state pacing at CL = 2000 ms, APD lost much of its sensitivity to pacing rate and remained relatively long. Conversely, when the clamp was to [Na^+^]_i_ and [Na^+^]_ss_ from steady state pacing at CL = 300 ms, the APD remained relatively short at all rates. Pacing rate dependent [Na^+^]_i_ and [Na^+^]_ss_ changes are linked to the AP via I_NaK_, which responds to [Na^+^]_i_ levels. I_NaK_ increased with [Na^+^]_i_ at fast rate. However it did not increase, regardless of the pacing rate, when [Na^+^]_i_ and [Na^+^]_ss_ were kept low (Na^+^ at CL = 2000 ms; Figure 16C, right). Moreover, APD remained long at all CLs when I_NaK_ was clamped to its slow rate waveform (not shown).

Steady state APD rate dependence was not completely eliminated by Na^+^ clamp alone. That is, clamping [Na^+^]_i_ and [Na^+^]_ss_ to slow rate values did not cause APD curves to become absolutely flat with respect to CL, especially at fast pacing rates (Figure 16A, shaded box CL = 300 to 700 ms, solid gray line). This signifies that other mechanisms are involved. When in addition to clamping [Na^+^]_i_ and [Na^+^]_ss_ to their slow rate values, we also reset the inactivation gates for late I_Na_, and especially for both late I_Na_ and I_CaL_ to their CL = 2000 ms values at the start of each beat, the APD curve flattened further at fast rates (Figure 16A, dashed gray and dashed-dot-dot gray lines, respectively). Importantly, resetting these inactivation gates alone, without also clamping Na^+^, had little effect on APD rate dependence (Figure 16B).

As described previously, without Na^+^ clamp, fast pacing caused late I_Na_ reduction and I_CaL_ increase; the former helped while the latter opposed APD shortening. However, with Na^+^ clamp, both currents became less inward with fast pacing. Thus, resetting I_CaL_ inactivation gates to slow rate values had different effects with, versus without Na^+^ clamping. Na^+^ clamp prolonged the APD. The prolongation and changed I_CaL_ behavior after Na^+^ clamp rendered late I_Na_ and I_CaL_ gate resetting more potent effectors of further AP prolongation; especially at fast rates where residual inactivation between beats was substantial.

Rate dependent Na^+^ changes only occurred with the steady state pacing protocol due to slow ion accumulation after lengthy pacing regimes. For APD restitution, clamping [Na^+^]_i_ and [Na^+^]_ss_ to values from S1 pacing during the S2 beat did not affect APD (Figure 16E). However, restitution was dramatically affected by resetting inactivation gates for late I_Na_ and/or I_CaL_ to their S1 starting values at the start of the S2 beat (Figure 16D). APD remained long for all DIs. Conversely, when late I_Na_ and/or I_CaL_ inactivation gates were reset to S2 starting values for DI = 5 ms, APD remained short for all DIs. Again, resetting these inactivation gates to their slow rate values had only minor effects on steady state APD rate dependence (Figure 16B).

**Figure 17:**
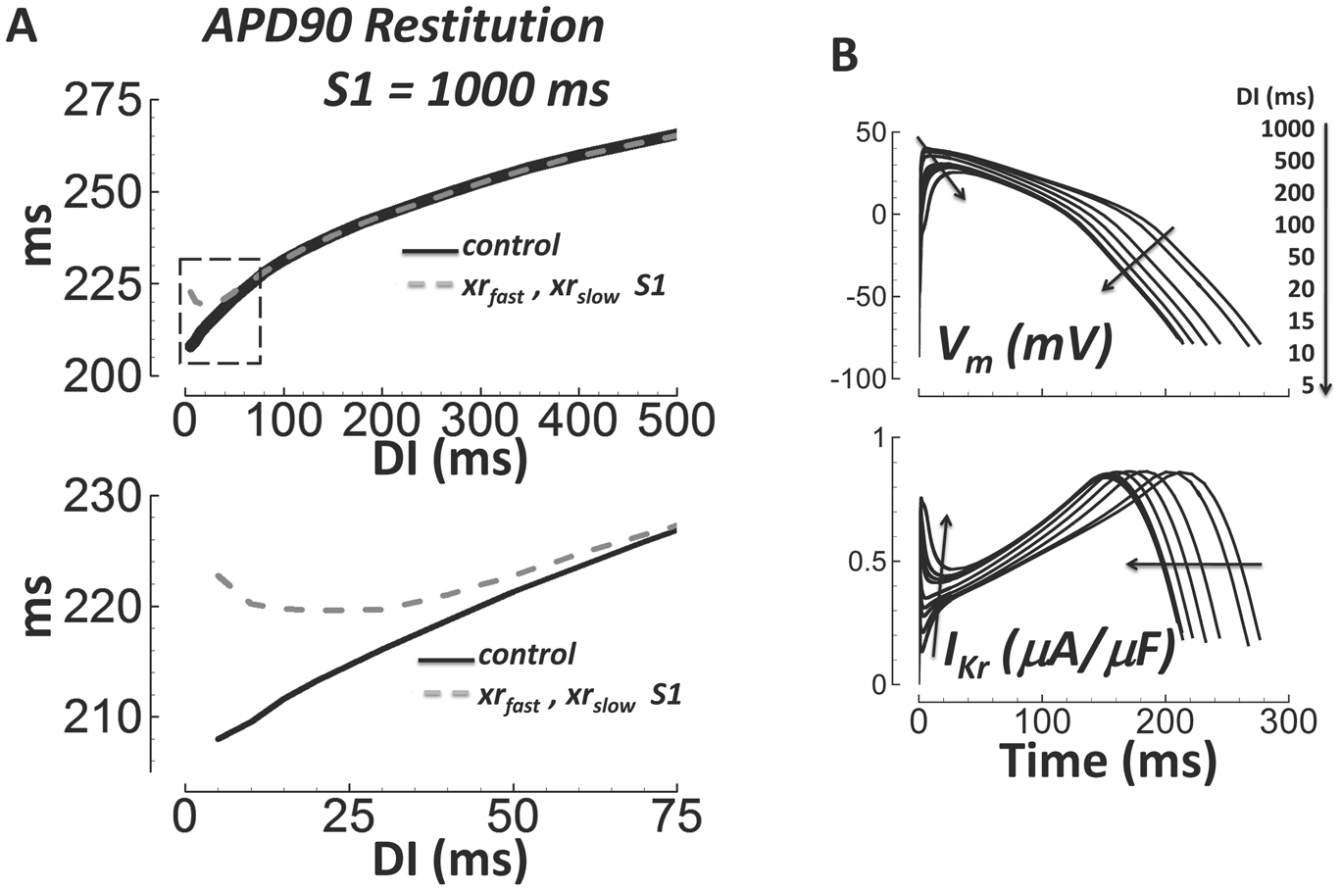
I_Kr_ deactivation is important for APD restitution at very short DIs. A) APD restitution in control (solid black), and when the fast and slow deactivation gates (xr_fast_ and xr_slow_) were reset to the DI = S1 = 1000 ms value at the start of the S2 beat (dashed gray). Bottom) Zoom in to more clearly show the consequence of deactivation resetting at short DIs (section outlined by dashed box above). B) Traces for the AP (top) and I_Kr_ (bottom) during the S2 beat at different DIs (indicated by arrows). Spiking in I_Kr_ occurred early during the AP at short DI. Spiking was caused by slow deactivation, increasing availability of I_Kr_.

At very short DIs, I_Kr_ played an important role in APD restitution. In Figure 17A, the fast and slow time dependent deactivation gates (xr_fast_ and xr_slow_, respectively) were reset to their value at DI = S1 = 1000 ms (dashed gray line, compare to control solid black line). Deactivation of I_Kr_ is slow (Figure 3B). For DI = S1, deactivation was complete between beats. At short DIs, it was incomplete at the start of the S2 beat, enhancing I_Kr_ availability (early I_Kr_ spiking, Figure 17B, bottom) and outward current that contributes to APD shortening. The enhanced availability only mattered at very short DIs, because at these DIs APD was short enough that increased outward current during phase-1 of the AP affected final repolarization time. Changes to currents during later AP phases 2 and 3 (during the plateau and early repolarization, e.g. late I_Na_ and I_CaL_), generally have greater impact on the APD. Early I_Kr_ spiking reduced maximum V_m_, which affected all other currents, including late I_Na_ and I_CaL_.

### Comparison with Other Human Ventricular AP Models

**Figure 18:**
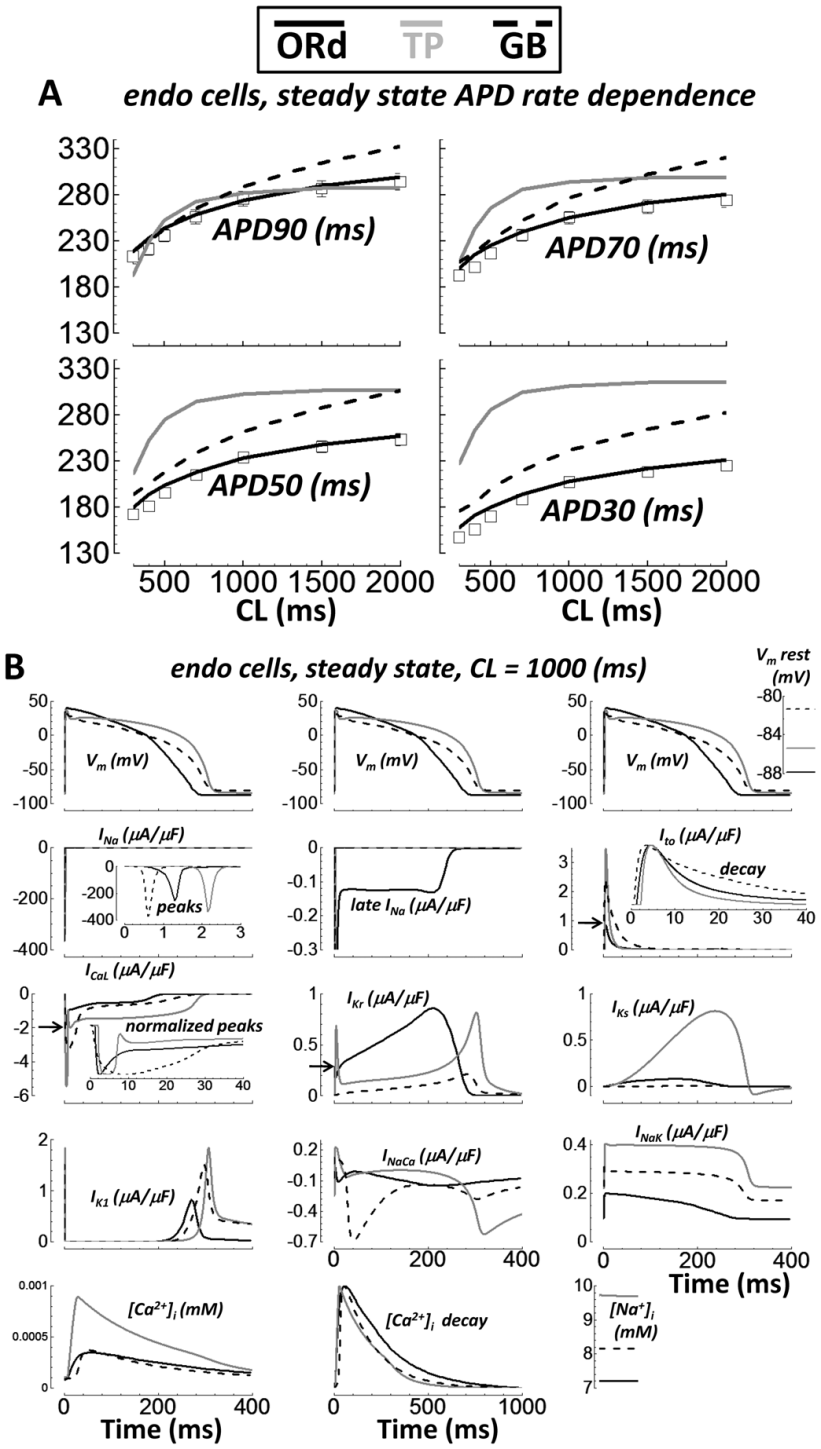
Comparison with other human ventricular AP models. Single endo cell simulations from ORd, TP, and GB models are solid black, gray, and dashed black lines, respectively. Experimental results (small tissue preparations) are white squares. A) APD rate dependence. Results for APD90, 70, 50 and 30 are shown top left, top right, bottom left, and bottom right, respectively. B) The AP, major currents, [Na^+^]_i_, and [Ca^2+^]_i_ at steady state for CL = 1000 ms. From left to right, top to bottom: AP (with V_m_ rest inset at far right), I_Na_ (inset shows peaks), late I_Na_ (not present in TP or GB models), I_to_ (inset shows decay rate), I_CaL_ (arrow shows ORd peak magnitude; inset shows normalized peaks, which are wide in TP and GB), I_Kr_ (arrow shows ORd early spike peak magnitude), I_Ks_, I_K1_, I_NaCa_, I_NaK_, [Ca^2+^]_i_, [Ca^2+^]_i_ decay rate, and [Na^+^]_i_.

Several important differences exist between the ORd model presented here and other human models (e.g. TP[9] and GB[10] models). Notably, model differences in the rate of repolarization and EAD formation were examined in direct comparison with experiments (Figures 7C, and Figure 11A, respectively). Readers wishing to simulate the human ventricular AP have a choice of models. To help further differentiate the models, additional comparisons are shown in Figure 18.

Undiseased human ventricular measurements of steady state rate dependence of APD90, 70, 50 and 30 were accurately reproduced by the ORd model (Figure 18A, same data as in Figure 7A). Rate dependence of APD90 is fairly accurate in the TP model. However, rate dependence of APD70, 50 and especially APD30 are not accurate. The GB model repolarization rate is more accurate, but divergence from the measurements is large for APD30. At fast pacing rates, GB model APD90 is accurate. Slow pacing APD90 is long compared with experiments (at CL = 2000 ms, APD90 is ∼40 ms longer than in experiments). In addition, APD rate dependence does not plateau at CL = 2000 ms.

In Figure 18B, the AP, major currents, and [Na^+^]_i_ and [Ca^2+^]_i_ were compared between models. Simulations were in single endo cells paced to steady state at CL = 1000 ms. Of note, the TP and GB models do not include late I_Na_. The width of the I_CaL_ peak and the morphology were model dependent. It was “cigar shaped” in the TP model. In the GB model, the I_CaL_ peak was broad and poorly defined. The ORd model I_CaL_ peak was sharp, as seen in undiseased human ventricle experiments (AP clamp, Figure 1D). I_Kr_ was relatively small in the GB model, but shared a similar morphology with the ORd model. The TP I_Kr_ morphology is characterized by an early spike and a wider late spike. The I_Ks_ density in the TP model was much larger than in the other models (∼10 fold larger). Density and morphology of I_NaCa_ was model dependent. I_NaCa_ was smallest in the ORd model (based on undiseased human measurements, Figure 2B). I_NaK_ was roughly 1.5 and 2 fold greater in GB and TP models, respectively, compared with ORd. The Ca^2+^ transient peak was much larger in the TP model than in the other models, which were similar to each other. The decay rate of [Ca^2+^]_i_ was somewhat slower in the ORd model (accurate to undiseased human measurements; Figure 12 panels C and D). Model [Na^+^]_i_ was 7.2, 8.2, and 9.7 mM in ORd, GB, and TP models, respectively.

## Discussion

Though the available undiseased human ventricle dataset has been missing essential elements, several human ventricle AP models have been developed and published. The Priebe and Beuckelmann model[35] lacks human specific data for reformulation of major currents, and so was based in large part on its guinea pig predecessor[80]. The TP model[81] and updated version[9] is easy to use, includes many reformulated currents, and simulates physiological restitution and alternans. However, both the TP and GB[10] models lack sufficient I_CaL_ data for validation, and cannot produce EADs. The GB model includes K^+^ current reformulations using undiseased human data for validation, but does not produce AP or Ca^2+^ transient alternans. EADs and alternans are both important mechanisms of arrhythmogenesis and should be reproduced in simulation studies of human arrhythmias. The Iyer et al. model[34] is based almost entirely on data from human channels expressed in non myocytes. Though the expressed channels are human, native myocyte ion channels in the ventricle are composed of a variety of protein isoform combinations, auxiliary subunits, cytoskeletal elements, and membrane lipid composition, all of which may influence channel behavior. Anchoring and other regulatory proteins present in native cells also define the local environment for I_CaL_ in particular[82], but are not present in expression systems.

Fink et al. modified the TP model[36] to include updated I_Kr_ and I_K1_ (with [K^+^]_o_ dependence) formulations, based on undiseased human ventricular measurements. The rate of AP repolarization in this modified scheme is more accurate compared with the original TP model. For these advantages, the model sacrifices runtime speed (Markov formulation is used for I_Kr_). Other core issues of the TP model carry over to this modified version (incorrect I_CaL_, non-physiologically large I_Ks_, and no EAD generation under appropriate conditions).

We believe that the new undiseased human data presented here are essential, and substantially increase human specific model accuracy. Due to extensive validation using these new data, our model reproduces all of the following important physiological behaviors: 1) CDI versus VDI inactivation of I_CaL_; 2) reformulated, detailed and accurate kinetics (using weighted time constants) for I_to_, I_NaCa_, I_K1_, I_Kr_, I_Ks_, fast I_Na_, and late I_Na_; 3) AP repolarization rate from 30% to 90% repolarization; 4) APD at all physiological pacing rates with/without block of major currents, 5) APD restitution with/without block of delayed rectifier currents; 6) transmural heterogeneity causing upright pseudo-ECG T-wave; 7) early afterdepolarizations (EADs); 8) effects of CaMK; and 9) AP and Ca^2+^ transient alternans.

### EADs and Repolarization Rate

One of the most important aspects of the model is its close correspondence to experimental measurements of not only APD90, but also to APD30, 50 and 70 at all physiologically relevant pacing rates and for S1S2 restitution. This large pool of data has previously been unavailable. Accurate repolarization rate (i.e. time between APD30 and 90) for the restitution protocol is crucial for simulating any phenomenon related to reentrant arrhythmia, where head-tail interactions determine refractoriness and vulnerability[83]. Use of new undiseased data for currents that are active during the plateau and phase-3 of the AP (I_CaL_, I_NaCa_, I_Kr_ and I_Ks_) contributed to the correct repolarization rate.

The rate of repolarization and its effects on I_CaL_ control EAD formation in this model, as in canonical EAD explanations [75], [84]. Failure of the TP and GB models to reproduce EADs may be due in part to their accelerated repolarization rates (Figure 7C). It may also be caused by inaccurate formulation of I_CaL_ inactivation, developed in absence of the essential undiseased human data presented here.

### Steady State APD Rate Dependence

Due to the small amplitude and rapid deactivation kinetics of I_Ks_ in the human ventricle in absence of β-adrenergic stimulation, it does not play a major role in determining APD, APD rate dependence, or APD restitution under basal conditions[85] (Figure 8). This is in contrast to guinea pig ventricle, where slower deactivation and larger amplitude I_Ks_ make it the most important current for steady state APD rate dependence (simulations[86] and experiments[87]). Phosphorylation by PKA in the case of β-adrenergic stimulation greatly enhances both the activation rate and amplitude of I_Ks_ [88]. With β-adrenergic stimulation, I_Ks_ plays an important role in steady state APD rate dependence[89]. Clearly, I_Ks_ is important under various circumstances – the AP repolarizes in human ventricle experiments even when I_Kr_ is blocked[85], and clinical long QT syndrome type 1 is caused by I_Ks_ loss of function[90]. Typically, isolated myocyte patch clamp experiments underestimate I_Ks_ due to enzymatic degradation[42]. In ORd, the role of I_Ks_ was validated using small tissue preparations, where selective I_Ks_ block prolonged APD, but only very modestly under basal conditions (no β-adrenergic stimulation, <15 milliseconds in experiments and simulations at CL = 1000 ms, Figure 8).

Block of I_Kr_ caused the most severe changes to the human AP (rate dependence and restitution, Figure 8). However, Supplement Figure S5 in Text S1, and Figure 15A show that I_Kr_ is rate independent, as in experiments[39] and therefore was not responsible for causing APD changes with pacing rate. Rather, our simulations identified rate dependent changes in I_NaK_ secondary to [Na^+^]_i_ accumulation as a primary cause of APD rate dependence (Figure 16A, 16C). This finding is not new. Simulations in dog ventricle[19], human atrium[91], and in the GB human ventricle[10] models all led to this conclusion. However, findings from the Iyer human model[34] differ, at least in part, regarding this mechanism. In the Iyer model, [Na^+^]_i_ affected APD rate dependence via I_NaCa_, which is primarily outward at fast rates. Rate dependence in the TP model[9] is less [Na^+^]_i_ dependent because, as Grandi discussed[10], I_Ks_ is exaggerated. Experiments by Pieske et al.[57] investigated [Na^+^]_i_ in heart failure, versus nonfailing human ventricular myocytes. Pieske experiments demonstrate that rate dependent [Na^+^]_i_ accumulation is an important phenomenon in health and disease. However, additional experiments are needed to determine whether and how [Na^+^]_i_ affects I_NaK_ and APD in human ventricle.

In addition to I_NaK_ and I_NaCa_ (both included in the ORd model), intracellular Na^+^ is also mediated by fluxes related to H^+^, CO_2_, and HCO_3_^-^ homeostasis. Exchangers and cotransporters move Na^+^ ions down the electrochemical gradient in order to offset the cost of H^+^, CO_2_, and HCO_3_^-^ pumping. Na^+^ rate dependent handling and consequently I_NaK_ should be affected by these processes, which were not explicitly included in the ORd model. In the absence of H^+^, CO_2_, and HCO_3_^-^ fluxes, it is possible that the role of I_NaK_ might have been over estimated. It is important to address this because I_NaK_ and its response to Na^+^ accumulation was a major cause of APD rate dependence in the model. Thus, we performed simulations where H^+^, CO_2_, and HCO_3_^-^ effects on Na^+^ were explicitly included, using Crampin and Smith equations[92] (Supplement Figure S12 in Text S1).

Quantitative details of Na^+^ handling, I_NaK_ and APD rate dependence were affected when we included H^+^, CO_2_, and HCO_3_^-^ handling processes. However, the qualitative outcomes were not affected. I_NaK_ increase with fast pacing, secondary to Na^+^ accumulation, was still the primary determinant of APD rate dependence during steady state pacing.

Removal of the effects of Na^+^ accumulation on steady state APD rate dependence by clamping [Na^+^]_i_ and [Na^+^]_ss_ did not completely eliminate APD rate dependence. Especially at fast rates (Figure 16A, shaded box CL = 300 to 700 ms, solid gray line), APD was not absolutely flat with respect to CL. APD rate dependence was largely unaffected by resetting inactivation gates for late I_Na_, and/or I_CaL_ to their slow rate values at the start of each beat (Figure 16B). Interestingly, if these gates were reset while also clamping Na^+^ to slow rate values, the APD-CL curve became almost completely flat, even at fast rates (Figure 16A, dashed gray and dashed-dot-dot gray lines, respectively). Thus, accumulation of Na^+^ and consequent effects on I_NaK_ is a major cause of APD rate dependence, however, not the only cause. Other currents also participate at fast pacing rates. Though the GB model[10] demonstrated the Na^+^/I_NaK_/APD rate dependence mechanism, it did not show the additional effects of late I_Na_ and I_CaL_. The GB model cannot show these multi-factorial causes of APD rate dependence because it does not include late Na^+^ current (Figure 18), and because I_CaL_ kinetics are inaccurate due to lack of experimental data.

Due to charge conservation, accumulation of [Na^+^]_i_ is associated with an equal reduction in [K^+^]_i_ and a volume converted [K^+^]_o_ increase in tissue clefts and interstitial spaces[93]. This can affect behavior by increasing I_K1_ (its [K^+^]_o_ sensitivity is included in this model), which depolarizes resting voltage and reduces excitability. However, our simulations represent experiments in an isolated myocyte in a large bath, where [K^+^]_o_ is constant. Even *in vivo*, [K^+^]_o_ is tightly controlled via regulation by the lymphatic system and kidneys.

### APD Restitution

We showed that in contrast to steady state rate dependence, [Na^+^]_i_ had no effect on APD restitution. Rather, restitution was primarily caused by the time course of recovery from inactivation of late I_Na_ and I_CaL_; processes which had little effect on steady state-rate dependence of APD (absent Na^+^ clamp). At very short DIs, slow deactivation of I_Kr_ caused increased availability and spiking, which helped shorten the APD. APD rate dependence was caused primarily by concentration changes, while APD restitution was caused by gating kinetics. Previous studies have not made this important distinction between steady state rate dependence and restitution mechanisms in human. The role of I_CaL_ and its inactivation kinetics in APD restitution reiterates the primacy of I_CaL_ in determining basic physiological behaviors, highlighting the importance of the new I_CaL_ experimental data, presented here, to model development and validation.

A role for late I_Na_ in restitution could not have been hypothesized using TP or GB models, which have no late I_Na_. The density of late I_Na_ was constrained in the ORd model by experiments from nonfailing human ventricular myocyte measurements by Maltsev et al.[51], where the late current was measured 200 ms after the start of the voltage clamp step (∼0.35 µA/µF I–V curve maximum). The maximum late I_Na_ during the free running AP model was much smaller (∼0.15 µA/µF, about half the I–V curve maximum) even at slow pacing rates, where late I_Na_ was largest. Late current is difficult to measure directly, and it is possible that the current density was overestimated due to selection bias. That is, late I_Na_ is small, and not all cells produced measureable late current (2 of 3 myocytes[51]). However, we consider the model density of late I_Na_ to be accurate based on model reproduction of experiments which consistently showed substantial APD90 shortening following application of 10 µM mexiletine in undiseased human myocardium (90% late I_Na_ block in simulations, Figure 8A).

### Ca^2+^ Cycling, CaMK and Alternans

Previously published human ventricle AP models have not incorporated the CaMK signaling pathway. Our human simulations show, as in dog simulations[31], [77], that CaMK plays an important role in determining frequency dependence of Ca^2+^ cycling (Figure 13). The model also shows that the integrated electrophysiological consequence of CaMK effects on target channels is minimal. That is, CaMK suppression had only minor effects on APD rate dependence and AP morphology. At very fast pacing (CLs <300 ms), the Ca^2+^ cycling consequences of CaMK phosphorylation were central to alternans formation. Suppression of CaMK eliminated alternans. CaMK related findings are in agreement with simulations using other models developed by our group[77], models from other groups[94], and experiments[95]. However, experiments showing the effects of pharmacological suppression of CaMK on rate dependent behaviors (e.g. by Wehrens et al.[96] with KN-93 in rabbit) should be performed in human ventricular myocytes to validate model predictions.

### Transmural Heterogeneity

The method used for implementation of the transmural cell types (M and epi cell), based on the thoroughly validated endo cell framework, was simplistic. That is, we considered that channel conductance was proportional to transmural gradients in mRNA or protein expression for alpha subunits of ion channels. Only in the case of I_to_ were functional current measurement data available[70]. Staying within error bars for mRNA or protein data[67], [68], [69], channel conductances were modulated so that the simulated transmural AP differences were consistent with experiments[50], [71].

The effect of transmural heterogeneity of accessory β-subunits was not considered in the transmural cell type definitions. However, in the case of I_Ks_, the KCNE1 β-subunit is transmurally heterogeneous. KCNE1 protein was about two times greater in M-cells compared to epi cells[69]. The presence of KCNE1 carries two important functional consequences 1) ∼5 fold slower activation and 2) ∼5 fold larger conductance[97]. Therefore, theoretically, twice as much KCNE1 in M-cells may increase the variable stoichiometry ratio of KCNE1 to alpha subunit KCNQ1[98], slowing activation and increasing conductance. We conducted simulations to evaluate the influence of KCNE1 heterogeneity on I_Ks_ and the AP (Supplement Figure S13 and related description in Text S1). Due to the small amplitude of human I_Ks_ in the absence of β-adrenergic stimulation, implementation of KCNE1 heterogeneity did not appreciably affect the AP (Supplement Figures S13 and S19 in Text S1, where transmural APDs are shown to be relatively insensitive to changes in I_Ks_ conductance). Interestingly, the simulated effects of KCNE1 on activation rate and conductance offset one another. That is, slowed activation and larger conductance in M-cells yielded I_Ks_ current that was remarkably close to the control case. Similar results were found for epi cell simulations: the effects of faster activation and reduced conductance were offsetting such that their combined effect was minimal.

### APD Accommodation

Steady state rate dependence of APD and APD restitution were the focus of the simulations and experiments in this study. However, the time course of APD response to abrupt changes in pacing rate has been shown in human by Franz et al.[99], and simulated in the TP model by Pueyo et al.[100] as a marker for arrhythmia risk. Simulations of APD accommodation in our model compare favorably to Franz experiments (same pacing protocols used in experiments were used in the simulations, Supplement Figure S14 in Text S1). Single exponential curves were fit to the time dependence of APD changes. For abrupt CL reduction from 750 to 480 ms, the time constant was 250 and 284 seconds in experiments and simulations, respectively. Time constants were 300 and 299 seconds in experiments and simulations, respectively, when CL was abruptly returned to 750 ms. When the CL reduction was more severe, from CL = 750 to 410 ms, the time constants were 252 and 165 seconds, in experiments and simulations, respectively. For return to CL = 750 ms, the time constants were 350 and 289 seconds, respectively. Pueyo used time to 90% accommodation to compare model with experiments demonstrating similar accuracy. Both simulation studies also show initial overshoot, or “notching”, as observed and described by Franz.

### Parameter Sensitivity

As in Romero et al.[101], we performed a sensitivity analysis to determine factors participating in important model outputs, including 1) steady state APD90 rate dependence (Supplement Figure S15 in Text S1), 2) S1S2 APD90 restitution (Supplement Figure S16 in Text S1), 3) rate dependence of maximum (systolic) [Ca^2+^]_i_ (Supplement Figure S17 in Text S1), 4) rate dependence of [Na^+^]_i_ (Supplement Figure S18 in Text S1), and 5) transmural cell type APD90 at steady state (Supplement Figure S19 in Text S1).

The findings from our analysis were similar to those shown by Romero et al. using the TP human AP model[101]. That is, in ORd and TP models, I_Kr_ and I_CaL_ affect APD90 while I_CaL_, I_NaCa_, and I_NaK_ affect peak [Ca^2+^]_i_. One important difference is the role for I_Ks_. A much larger role was played by I_Ks_ in the TP model (∼10 fold larger density than in other human models, Figure 18B). In the TP model, I_Ks_ is responsible for steady state rate dependence of the APD (shown by Grandi et al.[10]).

I_Kr_ conductance changes affect APD90 substantially in our model. This was expected, since I_Kr_ is the largest outward current (also in experiments, Figure 8, and in Romero's analysis using the TP model). Though I_Kr_ affects APD, it is not responsible for its rate dependence. Conductance changes in I_NaK_ did not substantially affect APD90 because I_NaK_ is a relatively small current. Yet, rate dependent changes in I_NaK_ (secondary to Na^+^ accumulation at fast rate) were the primary determinant of APD rate dependence. [Na^+^]_i_ at different pacing rates, and thus its relative changes with rate, was by far most sensitive to I_NaK_ conductance (Supplement Figure S18 in Text S1). This supports our strategy for setting I_NaK_ conductance to reproduce rate dependence of [Na^+^]_i_ in nonfailing human myocytes[57].

### Computational Tractability and Model Stability

To keep the ORd model computationally efficient and parameters well constrained, the Hodgkin-Huxley formalism was used in formulating current equations. This choice was made as a design principal with the thought that interested users can modularly replace any current or flux with more detailed Markov formulations of mutation or drug effects as desired (e.g.[53], [66]). Similarly, intracellular Ca^2+^ handling can be modified (e.g. more spatial detail, Markov ryanodine receptor implementation), or various signaling pathways and related effects on ion channels can be added (e.g.[31], [89], [102]). The basic ORd model has 41 state variables. In the absence of CaMK and its effects on target currents and fluxes, the number of state variables is 31.

Exclusion of Markov models increases parameter constraint. It also prevents the system of differential-algebraic equations from being overly stiff. This promotes model stability and computational tractability. Using the rapid integration technique described in Supplement Text S1 (Computational Methodology section), the model arrives at true and accurate steady state in under one minute of runtime (∼1000 beats are needed, depending on the CL, Visual C++ running on a desktop PC; more details in Supplement Text S1). ORd equations are all smoothly varying functions, free of singularities and “if” conditionals. Thus, the model can readily be implemented in any of a variety of automated numerical integrators, such as Matlab (The MathWorks Inc.), CellML (http://www.cellml.org/), CHASTE[103], or CARP (CardioSolv LLC.).

### Limitations

Direct measurement of I_NaK_ in the undiseased or nonfailing human ventricular myocyte is lacking. Therefore, I_NaK_ was validated by reproduction of important biophysical properties (Supplement Figure S7 in Text S1), and by reproduction of [Na^+^]_i_ rate dependence measured in nonfailing human ventricular myocytes (Pieske et al.[57], Figure 12A). However, independent and direct experimental measurement of I_NaK_ in undiseased or nonfailing human ventricular myocytes would provide additional support for the mechanistic conclusion that I_NaK_ changes secondary to Na^+^ accumulation at fast pacing rates is a major determinant of steady state APD rate dependence. This conclusion is consistent with several other modeling studies which proposed the same mechanism (dog ventricle[19], human atrium[91], and human ventricle[10]). The relationship between [Na^+^]_i_, I_NaK_ and steady state APD rate dependence was robust. It was not disrupted by including the effects of Na^+^/H^+^ and Na^+^/HCO_3_^-^ exchange fluxes on Na^+^ handling (Crampin and Smith equations[92], Supplement Figure S12 in Text S1). Na^+^ accumulation and I_NaK_ response were not the only cause of APD rate dependence in the ORd model. At fast pacing rates (CL = 300 to 700 ms), late I_Na_ and I_CaL_ were also involved (Figure 16A, and related discussion).

Measurements of undiseased human endo APs were performed in small tissue preparations (1–3 gram pieces). This was to avoid possible enzymatic degradation of K^+^ channel proteins[40], [42], affecting currents and the AP. However, electrical loading in tissue subtly affects behavior[19]. We performed simulations using a multicellular fiber model to include loading effects, which had only minor consequences (Figure S8).

APD was ∼275 ms in our human endo preparations at CL = 1000 ms, well matched by the model (273 ms). *In vivo* noninvasive electrocardiographic imaging of the activation-recovery interval, an indicator of the cellular epi APD, was ∼260 ms in healthy adults[104]. Human monophasic AP measurements are also in this range[58]. Measurements from Drouin et al. showed longer APDs (∼350 ms in endo cells on the cut transmural face at CL = 1000 ms). Having validated the endo model based on more than 100 of our own endo AP measurements, we thought it reasonable to use Drouin transmural APD ratios, rather than the uniformly longer APDs themselves, for validation of the transmural cell type models.

The presence of M cell APs in the nonfailing human heart was observed by Drouin et al.[50], and more recently by Glukhov et al.[71]. However, there is controversy regarding the M cell definition and its role in human. Our M cell model was based on data where the M cell was defined by its transmural location. The resulting simulated M cell AP corresponded with the “max cell” observed by Glukhov.

Recently, Sarkar and Sobie developed a method for quantitative analysis of parameter constraint and relationships between parameters and target outputs in AP models[105]. We did not apply this analysis during model development. However, the extensive validation of channel kinetics and the emergent response of the AP to a variety of dynamic pacing protocols, used in development and validation of the model, ensures sufficient parameter constraint. The parameter sensitivity tests we performed were instructive, though relatively limited (conductance changes only). Application of Sarkar and Sobie's analysis to our model is beyond the scope of this paper, but should provide worthwhile insights regarding inter-relatedness of processes in the human ventricle, in addition to formally testing parameter constraint.

## Materials and Methods

### Characteristics of Human Tissue

During the last 15 years, undiseased hearts were donated for research in compliance with the Declaration of Helsinki and were approved by the Scientific and Research Ethical Committee of the Medical Scientific Board of the Hungarian Ministry of Health (ETT-TUKEB), under ethical approval No 4991-0/2010-1018EKU (339/PI/010). Data from 140 hearts were used in this study. Of these, 78 were from male donors (56%). The average donor age was 41 years old with standard deviation of 12 years.

### Tissue Preparation

Tissue transport and ventricular endo preparations were performed as previously described[85]. Tissue was carefully pinned and placed in a modified Tyrodes superfusate (in mM: NaCl 115, KCl 4, CaCl_2_ 1.8, MgCl_2_ 1, NaHCO_3_ 20, and glucose 11, pH 7.35, 37°C), and point stimulation was via bipolar platinum electrodes. Drug solutions were made fresh on the day of use. Drugs included in this study were, in µM: E-4031 1, HMR-1556 1, nisoldipine 1, BaCl_2_ 100, ryanodine 5, mexiletine 10. Simulated application of these drugs was 70% I_Kr_ [59], and 90% I_Ks_ [60], I_CaL_ [61], I_K1_ [62], RyR[63], and late I_Na_ [64] block, respectively.

### Myocyte Isolation

Tissue transport and myocyte isolation for the undiseased donor hearts were as previously described[85]. Myocyte isolation commenced immediately upon arrival in the laboratory, using the perfusion disaggregation procedure, previously described[85].

### Electrophysiology

Data were obtained using conventional whole cell patch-clamp techniques. Micropipette fabrication and data acquisition were as described previously for undiseased donor heart[85]. Axopatch 200 amplifiers, Digidata 1200 converters, and pClamp software were used (Axon Instruments/Molecular Devices). Experiments were performed at 37°C.

The standard bath solution contained, in mM: NaCl 144, NaH_2_PO_4_ 0.33, KCl 4.0, CaCl_2_ 1.8, MgCl_2_ 0.53, Glucose 5.5, and HEPES 5.0 at pH of 7.4, and pipette solutions contained K-aspartate 100, KCl 25, K_2_ATP 5, MgCl_2_ 1, EGTA 10 and HEPES 5. The pH was adjusted to 7.2 by KOH (+15−20 mM K^+^).

For L-type Ca^2+^ current measurement, the bath solution contained in mM: tetraethylammonium chloride (TEA-Cl) 157, MgCl_2_ 0.5, HEPES 10, and 1 mM CaCl_2_, or BaCl_2_, or SrCl_2_ (pH 7.4 with CsOH). The pipette solution contained (in mM) CsCl 125, TEA-Cl 20, MgATP 5, creatine phosphate 3.6, EGTA 10, and HEPES 10 (pH 7.2 with CsOH).

For Na^+^/Ca^2+^ exchange current measurement, the bath solution contained, (in mM): NaCl 135, CsCl 10, CaCl2 1, MgCl_2_ 1, BaCl_2_ 0.2, NaH_2_PO_4_ 0.33, TEACl 10, HEPES 10, glucose 10 and (in µM) ouabain 20, nisoldipine 1, lidocaine 50, pH 7.4. The pipette solution contained (in mM): CsOH 140, aspartic acid 75, TEACl 20, MgATP 5, HEPES 10, NaCl 20, EGTA 20, CaCl2 10 (pH 7.2 with CsOH).

### Ca^2+^ Transient Florescence

Isolated myocytes from the undiseased donor hearts were used to measure the Ca^2+^ transient during point stimulation via bipolar platinum electrodes, indicated by Fura-2-AM, as was described previously[106]. Bath temperature was 37°C.

### Pacing Protocols

For both experiments and simulations, we determined APD at 30, 50, 70 and 90% of complete repolarization (APD30–90, in ms). The start of the AP was the time of maximum dV_m_/dt. The time of APDX occurred once membrane voltage was X% of the resting value. Resting voltage was measured immediately prior to each paced beat. For APD rate dependence, pacing was to steady state. For APD restitution (S1S2, or static restitution), S1 pacing was at cycle length (CL) = 1000 ms. The S2 beat was delivered at variable diastolic intervals (DIs), measured relative to APD90.

The dynamic restitution protocol was simulated as in experiments by Koller et al.[58]. Pacing was at a variety of rates (30 seconds at CLs from 230 to 1000 ms, no S2 beats). APD95 was plotted against DI (where DI = CL – APD95). Unlike static S1S2 restitution, the dynamic restitution protocol allows for more than one APD to be associated with a given DI. This is significant because bifurcation in the dynamic restitution curve is believed to be arrhythmogenic[107].

### Population Based CaMK effects

For all channels affected by CaMK, we created separate models for the fully CaMK phosphorylated channels, and the totally non phosphorylated channels. Then, based on the degree of CaMK activation (CaMK_active_), we determined the proportion of channels affected by CaMK. To calculate the CaMK affected current (or flux), we added the weighted sum of fully affected and totally unaffected channels, using the proportionality. The model employed for CaMK activity was validated previously[31], [77].

### Relative Weights in a Two Time Constant Scheme

When measurements called for a gating process to be represented by both a fast and a slow process, we included separate fast and slow gates. However, we did not simply multiply fast and slow gates to modulate conductance as others have done previously. To do so allows the fast process alone to completely control deactivation/inactivation, and the slow process alone to completely control activation/recovery. Rather, since measurements of bi-exponential behaviors provide the relative weight of fast/slow processes, we modeled the measurements accordingly, and used the weighted sum of fast and slow processes.

### Transmural Wedge Simulation

We computed the pseudo-ECG using a 1-dimensional model of the transmural wedge preparation[108], [109]. In brief, the spatially weighted sum of the voltage gradient was determined at a point 2 cm from the epi end of a heterogeneous multicellular fiber, along the fiber axis. Cells 1–60 were endo, 61–105 were M, and 106–165 were epi. The stimulus was delivered to cell 1. Cells 15 from both ends of the fiber were excluded from the gradient measurement due to confounding edge effects. Pacing was for 100 beats using steady state initial conditions from paced single cells.

### Equations, Computers, and Software

All model equations, hardware and software used, and rapid integration methods are provided in Supplement Text S1. Model code can be downloaded from the research section of our website: http://rudylab.wustl.edu.

## Supporting Information

Text S1 **Supplementary materials.**
(PDF)
