# Supplementary material for: Simulation of the Undiseased Human Cardiac Ventricular Action Potential: Model Formulation and Experimental Validation
Source: PLoS Comput Biol. 2011 May 26;7(5):e1002061. doi: 10.1371/journal.pcbi.1002061 (PMC3102752; doi:10.1371/journal.pcbi.1002061)
Supplement: Text S1 — Supplementary materials. (PDF) [file pcbi.1002061.s001.pdf]

SUPPLEMENTARY MATERIALS

DEFINITIONS AND ABBREVIATIONS ... 2

CURRENTS (μA/μF) ... 3
TIME DEPENDENT GATES ... 3
FLUXES (MILLIMOL/LITER/MS) ... 4
CALCIUM BUFFERS ... 4

ORD HUMAN MODEL BASIC PARAMETERS ... 5

STIMULUS ... 5
EXTERNAL CONCENTRATIONS ... 5
ORD MODEL INITIAL CONDITIONS ... 5
REVERSAL POTENTIALS ... 5
CELL GEOMETRY ... 6

ORD HUMAN MODEL CURRENTS ... 6

SODIUM CURRENT (I_{NA}) ... 6
TRANSIENT OUTWARD POTASSIUM CURRENT (I_{TO}) ... 8
L-TYPE CALCIUM CURRENT (I_{CAL}) ... 9
RAPID DELAYED RECTIFIER POTASSIUM CURRENT (I_{KR}) ... 11
SLOW DELAYED RECTIFIER POTASSIUM CURRENT (I_{KS}) ... 11
INWARD RECTIFIER POTASSIUM CURRENT (I_{K1}) ... 12
SODIUM/CALCIUM EXCHANGE CURRENT (I_{NACA}) ... 12
SODIUM/POTASSIUM ATPASE CURRENT (I_{NAK}) ... 14
BACKGROUND CURRENTS (I_{NAB}, I_{CAB}, I_{KB}) AND SARCOLEMMAL CALCIUM PUMP CURRENT (I_{PCA}) ... 15
VOLTAGE ... 16
CALCIUM/CALMODULIN-DEPENDENT PROTEIN KINASE (CAMK) ... 16

ORD HUMAN MODEL FLUXES ... 16

DIFFUSION FLUXES ($J_{DIFF,NA}$, $J_{DIFF,CA}$, $J_{DIFF,K}$) ... 16
SR CALCIUM RELEASE FLUX, VIA RYANODINE RECEPTOR (J_{REL}) ... 17
CALCIUM UPTAKE VIA SERCA PUMP (J_{UP}) ... 17
CALCIUM TRANSLOCATION FROM NSR TO JSR (J_{TR}) ... 18

ORD HUMAN MODEL CONCENTRATIONS AND BUFFERS ... 18

ORD HUMAN MODEL TRANSMURAL HETEROGENEITY ... 19

COMPUTATIONAL METHODOLOGY ... 19

HARDWARE AND SOFTWARE ... 19
RAPID INTEGRATION ... 20

SUPPLEMENTARY FIGURES ... 21

ADDITIONAL DETAILS FOR CURRENTS ... 21
APD RATE DEPENDENCE IN HOMOGENEOUS MULTICELLULAR FIBER ... 28
TRANSMURAL AP SIMULATIONS COMPARED WITH NONFAILING HUMAN OPTICAL MAPPING EXPERIMENTS ... 29

ALTERNANS SIMULATION IN COUPLED TISSUE 30
ALTERNANS WERE ELIMINATED BY SERCA2A UPREGULATION 31
EFFECTS OF H^+, CO_2 AND HCO_3^- ON Na^+ HANDLING, I_{NaK} AND APD RATE DEPENDENCE 32
EFFECT OF KCNE1 HETEROGENEITY ON TRANSMURAL I_{Ks} AND AP SIMULATIONS 33
APD ACCOMMODATION 34
PARAMETER SENSITIVITY ANALYSIS 35

REFERENCES 40

Definitions and Abbreviations

ORd model	O'Hara-Rudy dynamic human ventricular cell model
TP model	ten-Tusscher-Panfilov[1] human ventricular cell model
GB model	Grandi-Bers[2] human ventricular cell model
AP	action potential
APD	action potential duration (ms)
APDX	action potential duration to X% of resting membrane potential (ms)
CL	pacing cycle length (ms)
DI	diastolic interval, relative to APD90 (ms)
I-V Curve	current voltage relationship
CDI	Ca^{2+} dependent inactivation of L-type Ca^{2+} current
VDI	voltage dependent inactivation of L-type Ca^{2+} current
RyR	ryanodine receptor
V, or V_m	membrane voltage (mV)
R	gas constant, 8314 J/kmol/K
T	temperature, 310° K
F	Faraday constant, 96485 coul/mol
C_M	total membrane capacitance, 1 μF
E_S	reversal potential for ion S (mV)
R_{CG}	ratio between capacitive and geometric membrane areas (= 2)
A_{geo}	geometric area (cm^2)
A_{cap}	capacitive area (cm^2)
v_S	volume of compartment S (μL)
s_∞	steady state value of gate S
τ_s	time constant of gate S (ms)
$\tau_{s,fast}$, $\tau_{s,slow}$	fast/slow time constant of gate S (ms)
$A_{s,fast}$, $A_{s,slow}$	fraction of channels with gate S undergoing fast/slow process
z_s	valence of ion S
I_{stim}	stimulus current (μA/μF)
I_S	current through ion channel S (μA/μF)
$\overline{G_S}$	maximum conductance of ion channel S (mS/μF)
$K_{m,S}$	half-saturation concentration of molecule S (mM)
$\overline{I_S}$	maximum current carried through ion channel S (μA/μF)
P_S	permeability to ion S (cm/s)

$PR_{Q,S}$	permeability ratio of ion Q to ion S
γ_S	activity coefficient of ion S
J_S	ion flux S (mM/ms)
$[S]_Q$	concentration of ion S, in sub-cellular compartment Q (mM)
$\overline{[S]}$	maximum concentration of buffer S (mM)
myo	myoplasmic compartment (also abbreviated by small letter "i")
ss	subspace compartment (representing submembrane space near t-tubules)
SR	sarcoplasmic reticulum
jsr	junctional SR compartment
nsr	network SR compartment
Y	compartment Y (e.g. "i", "ss"), as in I_{NaCa} equations
CaMK	Ca^{2+}/calmodulin-dependent protein kinase II
$CaMK_{bound}$	fraction of CaMK binding sites bound to Ca^{2+}/calmodulin
$CaMK_{trap}$	fraction of autonomous CaMK binding sites with trapped calmodulin
$CaMK_{active}$	fraction of active CaMK binding sites
$CaMK_o$	fraction of active CaMK binding sites at equilibrium
α_{CaMK}, β_{CaMK}	(de)phosphorylation rates of CaMK (ms^{-1})
PLB	phospholamban
$\emptyset_{S,CaMK}$	fraction of channels of type S phosphorylated by CaMK

Currents (μA/μF)

I_{Na}	Na^+ current
I_{to}	transient outward K^+ current
I_{CaL}	Ca^{2+} current through the L-type Ca^{2+} channel
I_{CaNa}	Na^+ current through the L-type Ca^{2+} channel
I_{CaK}	K^+ current through the L-type Ca^{2+} channel
I_{Kr}	rapid delayed rectifier K^+ current
I_{Ks}	slow delayed rectifier K^+ current
I_{K1}	inward rectifier K^+ current
$I_{NaCa,i}$	myoplasmic component of Na^+/Ca^{2+} exchange current
$I_{NaCa,ss}$	subspace component of Na^+/Ca^{2+} exchange current
I_{NaCa}	total Na^+/Ca^{2+} exchange current
I_{NaK}	Na^+/K^+ ATPase current
I_{Nab}	Na^+ background current
I_{Cab}	Ca^{2+} background current
I_{Kb}	K^+ background current
I_{pCa}	sarcolemmal Ca^{2+} pump current

Time Dependent Gates

m	activation for fast I_{Na}
h_{fast}	fast development of inactivation for fast I_{Na}
h_{slow}	slow development of inactivation for fast I_{Na}
j	recovery from inactivation for fast I_{Na}
$h_{CaMK,fast}$	fast development of inactivation for CaMK phosphorylated fast I_{Na}

$h_{CaMK,slow}$	slow development of inactivation for CaMK phosphorylated fast I_{Na}
j_{CaMK}	recovery from inactivation for CaMK phosphorylated fast I_{Na}
m_L	activation for late I_{Na}
h_L	inactivation for late I_{Na}
$h_{L,CaMK}$	inactivation for CaMK phosphorylated late I_{Na}
a	activation for I_{to}
i_{fast}	fast inactivation for I_{to}
i_{slow}	slow inactivation for I_{to}
a_{CaMK}	activation for CaMK phosphorylated I_{to}
$i_{CaMK,fast}$	fast inactivation for CaMK phosphorylated I_{to}
$i_{CaMK,slow}$	slow inactivation for CaMK phosphorylated I_{to}
d	activation for I_{CaL}
f_{fast}	fast voltage dependent inactivation for I_{CaL}
f_{slow}	slow voltage dependent inactivation for I_{CaL}
$f_{Ca,fast}$	fast development of Ca^{2+} dependent inactivation for I_{CaL}
$f_{Ca,slow}$	slow development of Ca^{2+} dependent inactivation for I_{CaL}
j_{Ca}	recovery from Ca^{2+} dependent inactivation for I_{CaL}
n	fraction in Ca^{2+} dependent inactivation mode for I_{CaL}
$f_{CaMK,fast}$	fast development of Ca^{2+} dependent inactivation for CaMK phosphorylated I_{CaL}
$f_{Ca,CaMK,fast}$	slow development of Ca^{2+} dependent inactivation for CaMK phosphorylated I_{CaL}
$x_{r,fast}$	fast activation/deactivation for I_{Kr}
$x_{r,slow}$	slow activation/deactivation for I_{Kr}
x_{s1}	activation for I_{Ks}
x_{s2}	deactivation for I_{Ks}
x_{K1}	inactivation for I_{K1}

Fluxes (milliMol/Liter/ms)

$J_{diff,Na}$	diffusion of Na^+ from subspace to myoplasm
$J_{diff,K}$	diffusion of K^+ from subspace to myoplasm
$J_{diff,Ca}$	diffusion of Ca^{2+} from subspace to myoplasm
$J_{rel,NP}$	non-phosphorylated Ca^{2+} release, via ryanodine receptors, from jsr to myoplasm
$J_{rel,CaMK}$	CaMK phosphorylated Ca^{2+} release, via ryanodine receptors, from jsr to myoplasm
J_{rel}	total Ca^{2+} release, via ryanodine receptors, from jsr to myoplasm
$J_{up,NP}$	non-phosphorylated Ca^{2+} uptake, via SERCA pump, from myoplasm to nsr
$J_{up,CaMK}$	CaMK phosphorylated Ca^{2+} uptake, via SERCA pump, from myoplasm to nsr
J_{up}	total Ca^{2+} uptake, via SERCA pump, from myoplasm to nsr
J_{tr}	Ca^{2+} translocation from nsr to jsr

Calcium Buffers

CMDN	calmodulin, Ca^{2+} buffer in myoplasm
TRPN	troponin, Ca^{2+} buffer in myoplasm
BSR	anionic SR binding sites for Ca^{2+} in subspace
BSL	anionic sarcolemmal binding sites for Ca^{2+} buffer in subspace
CSQN	calsequestrin, Ca^{2+} buffer in JSR

β_Y buffer factor for compartment Y

ORd Human Model Basic Parameters

Stimulus

$$\text{amplitude} = -80.0\ \frac{\mu A}{\mu F}, \quad \text{duration} = 0.5\ \text{ms}$$

For charge conservation sake, stimulus has K^+ identity as described by Hund et al.[3].

External Concentrations

$[Na^+]_o = 140\ \text{mM}$
$[Ca^{2+}]_o = 1.8\ \text{mM}$
$[K^+]_o = 5.4\ \text{mM}$

ORd Model Initial Conditions

Single endocardial cell, at 1 Hz steady state, in diastole. There are 41 state variables.

$V = -87.84\ \text{mV}$
$[Na^+]_i = 7.23\ \text{mM}$
$[Na^+]_{ss} = 7.23\ \text{mM}$
$[K^+]_i = 143.79\ \text{mM}$
$[K^+]_{ss} = 143.79\ \text{mM}$
$[Ca^{2+}]_i = 8.54 \cdot 10^{-5}\ \text{mM}$
$[Ca^{2+}]_{ss} = 8.43 \cdot 10^{-5}\ \text{mM}$
$[Ca^{2+}]_{nsr} = 1.61\ \text{mM}$
$[Ca^{2+}]_{jsr} = 1.56\ \text{mM}$
$m = 0.0074621$
$h_{fast} = 0.692591$
$h_{slow} = 0.692574$
$j = 0.692477$
$h_{CaMK,slow} = 0.448501$
$j_{CaMK} = 0.692413$
$m_L = 0.000194015$
$h_L = 0.496116$
$h_{L,CaMK} = 0.265885$
$a = 0.00101185$
$i_{fast} = 0.999542$
$i_{slow} = 0.589579$
$a_{CaMK} = 0.000515567$
$i_{CaMK,fast} = 0.999542$
$i_{CaMK,slow} = 0.641861$
$d = 2.43015 \cdot 10^{-9}$
$f_{fast} = 1.0$
$f_{slow} = 0.910671$
$f_{Ca,fast} = 1.0$
$f_{Ca,slow} = 0.99982$
$j_{Ca} = 0.999977$
$n = 0.00267171$
$f_{CaMK,fast} = 1.0$
$f_{Ca,CaMK,fast} = 1.0$
$x_{r,fast} = 8.26608 \cdot 10^{-6}$
$x_{r,slow} = 0.453268$
$x_{s1} = 0.270492$
$x_{s2} = 0.0001963$
$x_{K1} = 0.996801$
$J_{rel,NP} = 2.53943 \cdot 10^{-5}$ mM/ms
$J_{rel,CaMK} = 3.17262 \cdot 10^{-7}$ mM/ms
$CaMK_{trap} = 0.0124065$

Reversal Potentials

$$E_{Na} = \frac{RT}{F} \cdot \ln\left(\frac{[Na^+]_o}{[Na^+]_i}\right)$$
$$E_K = \frac{RT}{F} \cdot \ln\left(\frac{[K^+]_o}{[K^+]_i}\right)$$
$$PR_{Na,K} = 0.01833, \quad E_{Ks} = \frac{RT}{F} \cdot \ln\left(\frac{[K^+]_o + PR_{Na,K} \cdot [Na^+]_o}{[K^+]_i + PR_{Na,K} \cdot [Na^+]_i}\right)$$

Cell Geometry

Cell geometry was approximated by a cylinder. Cell length (L) was about ten times longer than the radius (Forbes and Sperelakis)[4].

$L = 0.01$ cm, $\quad r = 0.0011$ cm

$v_{cell} = \pi \cdot r^2 \cdot L = 38 \cdot 10^{-6}\ \mu L$

$A_{geo} = 2\pi \cdot r^2 + 2\pi \cdot r \cdot L = 0.767 \cdot 10^{-4}\ cm^2$

$A_{cap} = R_{CG} \cdot A_{geo} = 2 \cdot A_{geo} = 1.534 \cdot 10^{-4}\ cm^2$

$v_{myo} = 0.68 \cdot v_{cell} = 25.84 \cdot 10^{-6}\ \mu L$

$v_{nsr} = 0.0552 \cdot v_{cell} = 2.098 \cdot 10^{-6}\ \mu L$

$v_{jsr} = 0.0048 \cdot v_{cell} = 0.182 \cdot 10^{-6}\ \mu L$

$v_{ss} = 0.02 \cdot v_{cell} = 0.76 \cdot 10^{-6}\ \mu L$

ORd Human Model Currents

Sodium Current (I_{Na})

$$m_\infty = \frac{1}{1 + \exp\left(\frac{-(V + 39.57)}{9.871}\right)}$$
$$\tau_m = \frac{1}{6.765 \cdot \exp\left(\frac{V + 11.64}{34.77}\right) + 8.552 \cdot \exp\left(\frac{-(V + 77.42)}{5.955}\right)}$$
$$\frac{dm}{dt} = \frac{m_\infty - m}{\tau_m}$$
$$h_\infty = \frac{1}{1 + \exp\left(\frac{V + 82.9}{6.086}\right)}$$
$$\tau_{h,fast} = \frac{1}{1.432 \cdot 10^{-5} \cdot \exp\left(\frac{-(V + 1.196)}{6.285}\right) + 6.149 \cdot \exp\left(\frac{V + 0.5096}{20.27}\right)}$$
$$\tau_{h,slow} = \frac{1}{0.009764 \cdot \exp\left(\frac{-(V + 17.95)}{28.05}\right) + 0.3343 \cdot \exp\left(\frac{V + 5.730}{56.66}\right)}$$
$$A_{h,fast} = 0.99, \quad A_{h,slow} = 0.01$$
$$\frac{dh_{fast}}{dt} = \frac{h_\infty - h_{fast}}{\tau_{h,fast}}$$

$$\frac{dh_{slow}}{dt} = \frac{h_\infty - h_{slow}}{\tau_{h,slow}}$$

$$h = A_{h,fast} \cdot h_{fast} + A_{h,slow} \cdot h_{slow}$$

$$j_\infty = h_\infty$$

$$\tau_j = 2.038 + \frac{1}{0.02136 \cdot \exp\left(\frac{-(V + 100.6)}{8.281}\right) + 0.3052 \cdot \exp\left(\frac{V + 0.9941}{38.45}\right)}$$

$$\frac{dj}{dt} = \frac{j_\infty - j}{\tau_j}$$

$$h_{CaMK,\infty} = \frac{1}{1 + \exp\left(\frac{V + 89.1}{6.086}\right)}$$

$$\tau_{h,CaMK,slow} = 3.0 \cdot \tau_{h,slow}$$

$$A_{h,CaMK,fast} = A_{h,fast}, \qquad A_{h,CaMK,slow} = A_{h,slow}$$

$$h_{CaMK,fast} = h_{fast}$$

$$\frac{dh_{CaMK,slow}}{dt} = \frac{h_{CaMK,\infty} - h_{CaMK,slow}}{\tau_{h,CaMK,slow}}$$

$$h_{CaMK} = A_{h,CaMK,fast} \cdot h_{CaMK,fast} + A_{h,CaMK,slow} \cdot h_{CaMK,slow}$$

$$j_{CaMK,\infty} = j_\infty$$

$$\tau_{j,CaMK} = 1.46 \cdot \tau_j$$

$$\frac{dj_{CaMK}}{dt} = \frac{j_{CaMK,\infty} - j_{CaMK}}{\tau_{j,CaMK}}$$

$$K_{m,CaMK} = 0.15, \quad \emptyset_{INa,CaMK} = \frac{1}{1 + \frac{K_{m,CaMK}}{CaMK_{active}}}$$

$$\overline{G_{Na,fast}} = 75\ mS/\mu F$$

$$I_{Na,fast} = \overline{G_{Na,fast}} \cdot (V - E_{Na}) \cdot m^3 \cdot \left(\left(1 - \emptyset_{INa,CaMK}\right) \cdot h \cdot j + \emptyset_{INa,CaMK} \cdot h_{CaMK} \cdot j_{CaMK}\right)$$

$$m_{L,\infty} = \frac{1}{1 + \exp\left(\frac{-(V + 42.85)}{5.264}\right)}$$

$$\tau_{m,L} = \tau_m$$

$$\frac{dm_L}{dt} = \frac{m_{L,\infty} - m_L}{\tau_{m,L}}$$

$$h_{L,\infty} = \frac{1}{1 + \exp\left(\frac{V + 87.61}{7.488}\right)}$$

$$\tau_{h,L} = 200\ ms$$

$$\frac{dh_L}{dt} = \frac{h_{L,\infty} - h_L}{\tau_{h,L}}$$

$$h_{L,CaMK,\infty} = \frac{1}{1 + \exp\left(\frac{V + 93.81}{7.488}\right)}$$

$$\tau_{h,LCaMK} = 3 \cdot \tau_{h,L}$$

$$\frac{dh_{L,CaMK}}{dt} = \frac{h_{L,CaMK,\infty} - h_{L,CaMK}}{\tau_{h,L,CaMK}}$$

$$K_{m,CaMK} = 0.15, \quad \emptyset_{INaL,CaMK} = \frac{1}{1 + \frac{K_{m,CaMK}}{CaMK_{active}}}$$

$$\overline{G_{Na,late}} = 0.0075\ mS/\mu F$$

$$I_{Na,late} = \overline{G_{Na,late}} \cdot (V - E_{Na}) \cdot m_L \cdot \left(\left(1 - \emptyset_{INaL,CaMK}\right) \cdot h_L + \emptyset_{INaL,CaMK} \cdot h_{L,CaMK}\right)$$

$$I_{Na} = I_{Na,fast} + I_{Na,late}$$

Transient Outward Potassium Current (I_{to})

$$a_\infty = \frac{1}{1 + \exp\left(\frac{-(V - 14.34)}{14.82}\right)}$$

$$\tau_a = \frac{1.0515}{\frac{1}{1.2089 \cdot \left(1 + \exp\left(\frac{-(V - 18.41)}{29.38}\right)\right)} + \frac{3.5}{1 + \exp\left(\frac{V + 100}{29.38}\right)}}$$

$$\frac{da}{dt} = \frac{a_\infty - a}{\tau_a}$$

$$i_\infty = \frac{1}{1 + \exp\left(\frac{V + 43.94}{5.711}\right)}$$

$$\tau_{i,fast} = 4.562 + \frac{1}{0.3933 \cdot \exp\left(\frac{-(V + 100)}{100}\right) + 0.08004 \cdot \exp\left(\frac{V + 50}{16.59}\right)}$$

$$\tau_{i,slow} = 23.62 + \frac{1}{0.001416 \cdot \exp\left(\frac{-(V + 96.52)}{59.05}\right) + 1.7808 \cdot 10^{-8} \cdot \exp\left(\frac{V + 114.1}{8.079}\right)}$$

$$A_{i,fast} = \frac{1}{1 + \exp\left(\frac{V - 213.6}{151.2}\right)}, \qquad A_{i,slow} = 1 - A_{i,fast}$$

$$\frac{di_{fast}}{dt} = \frac{i_\infty - i_{fast}}{\tau_{i,fast}}$$

$$\frac{di_{slow}}{dt} = \frac{i_\infty - i_{slow}}{\tau_{i,slow}}$$

$$i = A_{i,fast} \cdot i_{fast} + A_{i,slow} \cdot i_{slow}$$

$$a_{CaMK,\infty} = \frac{1}{1 + \exp\left(\frac{-(V - 24.34)}{14.82}\right)}$$

$$\tau_{a,CaMK} = \tau_a$$

$$\frac{da_{CaMK}}{dt} = \frac{a_{CaMK,\infty} - a_{CaMK}}{\tau_{a,CaMK}}$$

$$i_{CaMK,\infty} = i_\infty$$

$$\delta_{CaMK,develop} = 1.354 + \frac{10^{-4}}{\exp\left(\frac{V - 167.4}{15.89}\right) + \exp\left(\frac{-(V - 12.23)}{0.2154}\right)}$$

$$\delta_{CaMK,recover} = 1 - \frac{0.5}{1 + \exp\left(\frac{V + 70}{20}\right)}$$

$$\tau_{i,CaMK,fast} = \tau_{i,fast} \cdot \delta_{CaMK,develop} \cdot \delta_{CaMK,recover}$$
$$\tau_{i,CaMK,slow} = \tau_{i,slow} \cdot \delta_{CaMK,develop} \cdot \delta_{CaMK,recover}$$
$$A_{i,CaMK,fast} = A_{i,fast}, \qquad A_{i,CaMK,slow} = A_{i,slow}$$
$$\frac{di_{CaMK,fast}}{dt} = \frac{i_{CaMK,\infty} - i_{CaMK,fast}}{\tau_{i,CaMK,fast}}$$
$$\frac{di_{CaMK,slow}}{dt} = \frac{i_{CaMK,\infty} - i_{CaMK,slow}}{\tau_{i,CaMKslow}}$$
$$i_{CaMK} = A_{i,CaMK,fast} \cdot i_{CaMK,fast} + A_{i,CaMK,slow} \cdot i_{CaMK,slow}$$
$$K_{m,CaMK} = 0.15, \quad \emptyset_{Ito,CaMK} = \frac{1}{1 + \frac{K_{m,CaMK}}{CaMK_{active}}}$$
$$\overline{G_{to}} = 0.02 \text{ mS}/\mu\text{F}$$
$$I_{to} = \overline{G_{to}} \cdot (V - E_K) \cdot \left(\left(1 - \emptyset_{Ito,CaMK}\right) \cdot a \cdot i + \emptyset_{Ito,CaMK} \cdot a_{CaMK} \cdot i_{CaMK}\right)$$

L-type Calcium Current (I_{CaL})

$$d_\infty = \frac{1}{1 + \exp\left(\frac{-(V + 3.940)}{4.230}\right)}$$
$$\tau_d = 0.6 + \frac{1}{\exp\left(-0.05 \cdot (V + 6.0)\right) + \exp\left(0.09 \cdot (V + 14.0)\right)}$$
$$\frac{dd}{dt} = \frac{d_\infty - d}{\tau_d}$$
$$f_\infty = \frac{1}{1 + \exp\left(\frac{V + 19.58}{3.696}\right)}$$
$$\tau_{f,fast} = 7.0 + \frac{1}{0.0045 \cdot \exp\left(\frac{-(V + 20.0)}{10.0}\right) + 0.0045 \cdot \exp\left(\frac{V + 20.0}{10.0}\right)}$$
$$\tau_{f,slow} = 1000 + \frac{1}{0.000035 \cdot \exp\left(-\frac{V + 5.0}{4.0}\right) + 0.000035 \cdot \exp\left(\frac{V + 5.0}{6.0}\right)}$$
$$A_{f,fast} = 0.6, \quad A_{f,slow} = 1 - A_{f,fast}$$
$$\frac{df_{fast}}{dt} = \frac{f_\infty - f_{fast}}{\tau_{f,fast}}$$
$$\frac{df_{slow}}{dt} = \frac{f_\infty - f_{slow}}{\tau_{f,slow}}$$
$$f = A_{f,fast} \cdot f_{fast} + A_{f,slow} \cdot f_{slow}$$
$$f_{Ca,\infty} = f_\infty$$
$$\tau_{f,Ca,fast} = 7.0 + \frac{1}{0.04 \cdot \exp\left(-\frac{V - 4.0}{7.0}\right) + 0.04 \cdot \exp\left(\frac{V - 4.0}{7.0}\right)}$$
$$\tau_{f,Ca,slow} = 100 + \frac{1}{0.00012 \cdot \exp\left(\frac{-V}{3.0}\right) + 0.00012 \cdot \exp\left(\frac{V}{7.0}\right)}$$
$$A_{f,Ca,fast} = 0.3 + \frac{0.6}{1.0 + \exp\left(\frac{V - 10.0}{10.0}\right)}, \quad A_{f,Ca,slow} = 1 - A_{f,Ca,fast}$$

$$\frac{df_{Ca,fast}}{dt} = \frac{f_{Ca,\infty} - f_{Ca,fast}}{\tau_{f,Ca,fast}}$$
$$\frac{df_{Ca,slow}}{dt} = \frac{f_{Ca,\infty} - f_{Ca,slow}}{\tau_{f,Ca,slow}}$$
$$f_{Ca} = A_{f,Ca,fast} \cdot f_{Ca,fast} + A_{f,Ca,slow} \cdot f_{Ca,slow}$$
$$j_{Ca,\infty} = f_{Ca,\infty}$$
$$\tau_{j,Ca} = 75.0$$
$$\frac{dj_{Ca}}{dt} = \frac{j_{Ca,\infty} - j_{Ca}}{\tau_{j,Ca}}$$
$$f_{CaMK,\infty} = f_{\infty}$$
$$\tau_{f,CaMK,fast} = 2.5 \cdot \tau_{f,fast}$$
$$A_{f,CaMK,fast} = A_{f,fast}, \quad A_{f,CaMK,slow} = A_{f,slow}$$
$$\frac{df_{CaMK,fast}}{dt} = \frac{f_{CaMK,\infty} - f_{CaMK,fast}}{\tau_{f,CaMK,fast}}$$
$$f_{CaMK,slow} = f_{slow}$$
$$f_{CaMK} = A_{f,CaMK,fast} \cdot f_{CaMK,fast} + A_{f,CaMK,slow} \cdot f_{CaMK,slow}$$
$$f_{Ca,CaMK,\infty} = f_{\infty}$$
$$\tau_{f,Ca,CaMK,fast} = 2.5 \cdot \tau_{f,Ca,fast}$$
$$A_{f,Ca,CaMK,fast} = A_{f,Ca,fast}, \quad A_{f,Ca,CaMK,slow} = A_{f,Ca,slow}$$
$$\frac{df_{Ca,CaMK,fast}}{dt} = \frac{f_{Ca,CaMK,\infty} - f_{Ca,CaMK,fast}}{\tau_{f,Ca,CaMK,fast}}$$
$$f_{Ca,CaMK,slow} = f_{Ca,slow}$$
$$f_{Ca,CaMK} = A_{f,Ca,CaMK,fast} \cdot f_{Ca,CaMK,fast} + A_{f,CaMK,slow} \cdot f_{CaMK,slow}$$
$$K_{m,n} = 0.002, \quad k_{+2,n} = 1000.0, \quad k_{-2,n} = j_{Ca} \cdot 1.0$$
$$\alpha_n = \frac{1.0}{\frac{k_{+2,n}}{k_{-2,n}} + \left(1 + \frac{K_{m,n}}{[Ca^{2+}]_{ss}}\right)^{4.0}}$$
$$\frac{dn}{dt} = \alpha_n \cdot k_{+2,n} - n \cdot k_{-2,n}$$
$$P_{Ca} = 0.0001 \frac{cm}{s}$$
$$\gamma_{Cai} = 1.0, \quad \gamma_{Cao} = 0.341, \quad z_{Ca} = 2$$
$$\Psi_{Ca} = {z_{Ca}}^2 \cdot \frac{VF^2}{RT} \cdot \frac{\gamma_{Cai} \cdot [Ca^{2+}]_{ss} \cdot \exp\left(\frac{z_{Ca}VF}{RT}\right) - \gamma_{Cao} \cdot [Ca^{2+}]_o}{\exp\left(\frac{z_{Ca}VF}{RT}\right) - 1.0}$$
$$\overline{I_{CaL}} = P_{Ca} \cdot \Psi_{Ca}$$
$$P_{CaNa} = 0.00125 \cdot P_{Ca}, \quad \gamma_{Nai} = 0.75, \quad \gamma_{Nao} = 0.75, \quad z_{Na} = 1$$
$$\Psi_{CaNa} = {z_{Na}}^2 \cdot \frac{VF^2}{RT} \cdot \frac{\gamma_{Nai} \cdot [Na^+]_{ss} \cdot \exp\left(\frac{z_{Na}VF}{RT}\right) - \gamma_{Nao} \cdot [Na^+]_o}{\exp\left(\frac{z_{Na}VF}{RT}\right) - 1.0}$$
$$\overline{I_{CaNa}} = P_{CaNa} \cdot \Psi_{CaNa}$$
$$P_{CaK} = 3.574 \cdot 10^{-4} \cdot P_{Ca,}, \quad \gamma_{Ki} = 0.75, \quad \gamma_{Ko} = 0.75, \quad z_K = 1$$
$$\Psi_{CaK} = {z_K}^2 \cdot \frac{VF^2}{RT} \cdot \frac{\gamma_{Ki} \cdot [K^+]_{ss} \cdot \exp\left(\frac{z_K VF}{RT}\right) - \gamma_{Ko} \cdot [K^+]_o}{\exp\left(\frac{z_K VF}{RT}\right) - 1.0}$$
$$\overline{I_{CaK}} = P_{CaK} \cdot \Psi_{CaK}$$

$$\overline{P_{Ca,CaMK}} = 1.1 \cdot P_{Ca}$$
$$\overline{I_{CaL,CaMK}} = P_{Ca,CaMK} \cdot \Psi_{Ca}$$
$$\overline{P_{CaNa,CaMK}} = 0.00125 \cdot P_{Ca,CaMK}$$
$$\overline{I_{CaNa,CaMK}} = P_{CaNa,CaMK} \cdot \Psi_{CaNa}$$
$$\overline{P_{CaK,CaMK}} = 3.574 \cdot 10^{-4} \cdot P_{Ca,CaMK}$$
$$\overline{I_{CaK,CaMK}} = P_{CaK,CaMK} \cdot \Psi_{CaK}$$
$$K_{m,CaMK} = 0.15, \quad \emptyset_{ICaL,CaMK} = \frac{1}{1 + \frac{K_{m,CaMK}}{CaMK_{active}}}$$
$$I_{CaL} = \overline{I_{CaL}} \cdot d \cdot \left(1 - \emptyset_{ICaL,CaMK}\right) \cdot \left(f \cdot (1-n) + f_{Ca} \cdot n \cdot j_{Ca}\right) + \overline{I_{CaL,CaMK}} \cdot d \cdot \emptyset_{ICaL,CaMK} \cdot \left(f_{CaMK} \cdot (1-n) + f_{Ca,CaMK} \cdot n \cdot j_{Ca}\right)$$
$$I_{CaNa} = \overline{I_{CaNa}} \cdot d \cdot \left(1 - \emptyset_{ICaL,CaMK}\right) \cdot \left(f \cdot (1-n) + f_{Ca} \cdot n \cdot j_{Ca}\right) + \overline{I_{CaNa,CaMK}} \cdot d \cdot \emptyset_{ICaL,CaMK} \cdot \left(f_{CaMK} \cdot (1-n) + f_{Ca,CaMK} \cdot n \cdot j_{Ca}\right)$$
$$I_{CaK} = \overline{I_{CaK}} \cdot d \cdot \left(1 - \emptyset_{ICaL,CaMK}\right) \cdot \left(f \cdot (1-n) + f_{Ca} \cdot n \cdot j_{Ca}\right) + \overline{I_{CaK,CaMK}} \cdot d \cdot \emptyset_{ICaL,CaMK} \cdot \left(f_{CaMK} \cdot (1-n) + f_{Ca,CaMK} \cdot n \cdot j_{Ca}\right)$$

Rapid Delayed Rectifier Potassium Current (I_{Kr})

$$x_{r,\infty} = \frac{1}{1 + \exp\left(\frac{-(V + 8.337)}{6.789}\right)}$$
$$\tau_{xr,fast} = 12.98 + \frac{1}{0.3652 \cdot \exp\left(\frac{V - 31.66}{3.869}\right) + 4.123 \cdot 10^{-5} \cdot \exp\left(\frac{-(V - 47.78)}{20.38}\right)}$$
$$\tau_{xr,slow} = 1.865 + \frac{1}{0.06629 \cdot \exp\left(\frac{V - 34.70}{7.355}\right) + 1.128 \cdot 10^{-5} \cdot \exp\left(\frac{-(V - 29.74)}{25.94}\right)}$$
$$A_{xr,fast} = \frac{1}{1 + \exp\left(\frac{V + 54.81}{38.21}\right)}, \qquad A_{x,r,slow} = 1 - A_{xr,fast}$$
$$\frac{dx_{r,fast}}{dt} = \frac{x_{r,\infty} - x_{r,fast}}{\tau_{x,r,fast}}$$
$$\frac{dx_{r,slow}}{dt} = \frac{x_{r,\infty} - x_{r,slow}}{\tau_{x,r,slow}}$$
$$x_r = A_{x,r,fast} \cdot x_{r,fast} + A_{x,r,slow} \cdot x_{r,slow}$$
$$R_{Kr} = \frac{1}{\left(1 + \exp\left(\frac{V + 55}{75}\right)\right) \cdot \left(1 + \exp\left(\frac{V - 10}{30}\right)\right)}$$
$$\overline{G_{Kr}} = 0.046 \text{ mS}/\mu\text{F}$$
$$I_{Kr} = \overline{G_{Kr}} \cdot \sqrt{\frac{[K^+]_o}{5.4}} \cdot x_r \cdot R_{Kr} \cdot (V - E_K)$$

Slow Delayed Rectifier Potassium Current (I_{Ks})

$$x_{s1,\infty} = \frac{1}{1+\exp\left(\frac{-(V+11.60)}{8.932}\right)}$$

$$\tau_{x,s1} = 817.3 + \frac{1}{2.326\cdot 10^{-4}\cdot\exp\left(\frac{V+48.28}{17.80}\right) + 0.001292\cdot\exp\left(\frac{-(V+210.0)}{230.0}\right)}$$

$$\frac{dx_{s1}}{dt} = \frac{x_{s1,\infty} - x_{s1}}{\tau_{x,s1}}$$

$$x_{s2,\infty} = x_{s1,\infty}$$

$$\tau_{x,s2} = \frac{1}{0.01\cdot\exp\left(\frac{V-50}{20}\right) + 0.0193\cdot\exp\left(\frac{-(V+66.54)}{31}\right)}$$

$$\frac{dx_{s2}}{dt} = \frac{x_{s2,\infty} - x_{s2}}{\tau_{x,s2}}$$

$$\overline{G_{Ks}} = 0.0034\ \mathrm{mS/\mu F}$$

$$I_{Ks} = \overline{G_{Ks}}\cdot\left(1+\frac{0.6}{1+\left(\frac{3.8\cdot 10^{-5}}{[Ca^{2+}]_i}\right)^{1.4}}\right)\cdot x_{s1}\cdot x_{s2}\cdot(V - E_{Ks})$$

Inward Rectifier Potassium Current (I_{K1})

$$x_{K1,\infty} = \frac{1}{1+\exp\left(-\frac{V+2.5538\cdot[K^+]_o+144.59}{1.5692\cdot[K^+]_o+3.8115}\right)}$$

$$\tau_{x,K1} = \frac{122.2}{\exp\left(\frac{-(V+127.2)}{20.36}\right) + \exp\left(\frac{V+236.8}{69.33}\right)}$$

$$\frac{dx_{K1}}{dt} = \frac{x_{K1,\infty} - x_{K1}}{\tau_{x,K1}}$$

$$R_{K1} = \frac{1}{1+\exp\left(\frac{V+105.8-2.6\cdot[K^+]_o}{9.493}\right)}$$

$$\overline{G_{K1}} = 0.1908\ \mathrm{mS/\mu F}$$

$$I_{K1} = \overline{G_{K1}}\cdot\sqrt{[K^+]_o}\cdot x_{K1}\cdot R_{K1}\cdot(V - E_K)$$

Sodium/Calcium Exchange Current (I_{NaCa})

For, $Y \in \{i, ss\}$

$$k_{Na1} = 15\ \mathrm{mM},\quad k_{Na2} = 5\ \mathrm{mM},\quad k_{Na3} = 88.12\ \mathrm{mM},\quad k_{asymm} = 12.5$$

$$\omega_{Na} = 6\cdot 10^4\ \mathrm{Hz},\quad \omega_{Ca} = 6\cdot 10^4\ \mathrm{Hz},\quad \omega_{NaCa} = 5\cdot 10^3\ \mathrm{Hz}$$

$$k_{Ca,on} = 1.5\cdot 10^6\frac{\mathrm{mM}}{\mathrm{ms}},\quad k_{Ca,off} = 5\cdot 10^3\ \mathrm{Hz}$$

$$q_{Na} = 0.5224,\quad q_{Ca} = 0.1670$$

$$h_{Ca} = \exp\left(\frac{q_{Ca}VF}{RT}\right),\quad h_{Na} = \exp\left(\frac{q_{Na}VF}{RT}\right)$$

$$h_1 = 1 + \frac{[Na^+]_Y}{k_{Na3}}(1 + h_{Na})$$
$$h_2 = \frac{[Na^+]_Y \cdot h_{Na}}{k_{Na3} \cdot h_1}$$
$$h_3 = \frac{1}{h_1}$$
$$h_4 = 1 + \frac{[Na^+]_Y}{k_{Na1}}\left(1 + \frac{[Na^+]_Y}{k_{Na2}}\right)$$
$$h_5 = \frac{{[Na^+]_Y}^2}{h_4 \cdot k_{Na1} \cdot k_{Na2}}$$
$$h_6 = \frac{1}{h_4}$$
$$h_7 = 1 + \frac{[Na^+]_o}{k_{Na3}}\left(1 + \frac{1}{h_{Na}}\right)$$
$$h_8 = \frac{[Na^+]_o}{k_{Na3} \cdot h_{Na} \cdot h_7}$$
$$h_9 = \frac{1}{h_7}$$
$$h_{10} = k_{asymm} + 1 + \frac{[Na^+]_o}{k_{Na1}}\left(1 + \frac{[Na^+]_o}{k_{Na2}}\right)$$
$$h_{11} = \frac{{[Na^+]_o}^2}{h_{10} \cdot k_{Na1} \cdot k_{Na2}}$$
$$h_{12} = \frac{1}{h_{10}}$$
$$k_1 = h_{12} \cdot [Ca^{2+}]_o \cdot k_{Ca,on}$$
$$k_2 = k_{Ca,off}$$
$$k_3' = h_9 \cdot \omega_{Ca}$$
$$k_3'' = h_8 \cdot \omega_{NaCa}$$
$$k_3 = k_3' + k_3''$$
$$k_4' = \frac{h_3 \cdot \omega_{Ca}}{h_{Ca}}$$
$$k_4'' = h_2 \cdot \omega_{NaCa}$$
$$k_4 = k_4' + k_4''$$
$$k_5 = k_{Ca,off}$$
$$k_6 = h_6 \cdot [Ca^{2+}]_Y \cdot k_{Ca,on}$$
$$k_7 = h_5 \cdot h_2 \cdot \omega_{Na}$$
$$k_8 = h_8 \cdot h_{11} \cdot \omega_{Na}$$
$$x_1 = k_2 \cdot k_4 \cdot (k_7 + k_6) + k_5 \cdot k_7 \cdot (k_2 + k_3)$$
$$x_2 = k_1 \cdot k_7 \cdot (k_4 + k_5) + k_4 \cdot k_6 \cdot (k_1 + k_8)$$
$$x_3 = k_1 \cdot k_3 \cdot (k_7 + k_6) + k_8 \cdot k_6 \cdot (k_2 + k_3)$$
$$x_4 = k_2 \cdot k_8 \cdot (k_4 + k_5) + k_3 \cdot k_5 \cdot (k_1 + k_8)$$
$$E_1 = \frac{x_1}{x_1 + x_2 + x_3 + x_4}$$
$$E_2 = \frac{x_2}{x_1 + x_2 + x_3 + x_4}$$
$$E_3 = \frac{x_3}{x_1 + x_2 + x_3 + x_4}$$

$$E_4 = \frac{x_4}{x_1 + x_2 + x_3 + x_4}$$
$$K_{mCaAct} = 150 \cdot 10^{-6}\ \mathrm{mM}$$
$$\mathrm{allo}_Y = \frac{1}{1 + \left(\frac{K_{mCaAct}}{[Ca^{2+}]_Y}\right)^2}$$
$$J_{NaCa,Na,Y} = 3 \cdot (E_4 \cdot k_7 - E_1 \cdot k_8) + E_3 \cdot k_4'' - E_2 \cdot k_3''$$
$$J_{NaCa,Ca,Y} = E_2 \cdot k_2 - E_1 \cdot k_1$$
$$\underline{z_{Na} = 1}, \quad z_{Ca} = 2$$
$$\overline{G_{NaCa}} = 0.0008\ \mu A/\mu F$$
$$I_{NaCa,i} = \overline{G_{NaCa}} \cdot 0.8 \cdot \mathrm{allo}_i \cdot \left(z_{Na} \cdot J_{NaCa,Na,i} + z_{Ca} \cdot J_{NaCa,Ca,i}\right)$$
$$I_{NaCa,ss} = \overline{G_{NaCa}} \cdot 0.2 \cdot \mathrm{allo}_{ss} \cdot \left(z_{Na} \cdot J_{NaCa,Na,ss} + z_{Ca} \cdot J_{NaCa,Ca,ss}\right)$$
$$I_{NaCa} = I_{NaCa,i} + I_{NaCa,ss}$$

Sodium/Potassium ATPase Current (I_{NaK})

$$k_1^+ = 949.5\ \mathrm{Hz}, \quad k_1^- = 182.4\ \mathrm{mM}^{-1}, \quad k_2^+ = 687.2\ \mathrm{Hz}, \quad k_2^- = 39.4\ \mathrm{Hz}$$
$$k_3^+ = 1899\ \mathrm{Hz}, \quad k_3^- = 79300\ \mathrm{Hz} \cdot \mathrm{mM}^{-2}, \quad k_4^+ = 639.0\ \mathrm{Hz}, \quad k_4^- = 40\ \mathrm{Hz}$$
$$K_{Nai}^o = 9.073\ \mathrm{mM}, \quad K_{Nao}^o = 27.78\ \mathrm{mM}$$
$$\Delta = -0.1550$$
$$K_{Nai} = K_{Nai}^o \cdot \exp\left(\frac{\Delta \cdot V \cdot F}{3 \cdot R \cdot T}\right), \quad K_{Nao} = K_{Nao}^o \cdot \exp\left(\frac{(1-\Delta) \cdot V \cdot F}{3 \cdot R \cdot T}\right)$$
$$K_{Ki} = 0.5\ \mathrm{mM}, \quad K_{Ko} = 0.3582\ \mathrm{mM}$$
$$[MgADP] = 0.05, \quad [MgATP] = 9.8$$
$$K_{MgATP} = 1.698 \cdot 10^{-7}\ \mathrm{mM}$$
$$[H^+] = 10^{-7}\ \mathrm{mM}$$
$$[\Sigma P] = 4.2\ \mathrm{mM}$$
$$K_{H,P} = 1.698 \cdot 10^{-7}\ \mathrm{mM}, \quad K_{Na,P} = 224\ \mathrm{mM}, \quad K_{K,P} = 292\ \mathrm{mM}$$
$$[P] = [\Sigma P] \Big/ \left(1 + \frac{[H^+]}{K_{H,P}} + \frac{[Na^+]_i}{K_{Na,P}} + \frac{[K^+]_i}{K_{K,P}}\right)$$
$$\alpha_1 = \frac{k_1^+ \left(\frac{[Na^+]_i}{K_{Nai}}\right)^3}{\left(1 + \frac{[Na^+]_i}{K_{Nai}}\right)^3 + \left(1 + \frac{[K^+]_i}{K_{Ki}}\right)^2 - 1}$$
$$\beta_1 = k_1^- \cdot [MgADP]$$
$$\alpha_2 = k_2^+$$
$$\beta_2 = \frac{k_2^- \left(\frac{[Na^+]_o}{K_{Nao}}\right)^3}{\left(1 + \frac{[Na^+]_o}{K_{Nao}}\right)^3 + \left(1 + \frac{[K^+]_o}{K_{Ko}}\right)^2 - 1}$$
$$\alpha_3 = \frac{k_3^+ \left(\frac{[K^+]_o}{K_{Ko}}\right)^2}{\left(1 + \frac{[Na^+]_o}{K_{Nao}}\right)^3 + \left(1 + \frac{[K^+]_o}{K_{Ko}}\right)^2 - 1}$$

$$\beta_3 = \frac{k_3^- \cdot [P] \cdot [H^+]}{1 + \frac{[MgATP]}{K_{MgATP}}}$$

$$\alpha_4 = \frac{k_4^+ \cdot \frac{[MgATP]}{K_{MgATP}}}{1 + \frac{[MgATP]}{K_{MgATP}}}$$

$$\beta_4 = \frac{k_4^- \left(\frac{[K^+]_i}{K_{Ki}}\right)^2}{\left(1 + \frac{[Na^+]_i}{K_{Nai}}\right)^3 + \left(1 + \frac{[K^+]_i}{K_{Ki}}\right)^2 - 1}$$

$$x_1 = \alpha_4 \cdot \alpha_1 \cdot \alpha_2 + \beta_2 \cdot \beta_4 \cdot \beta_3 + \alpha_2 \cdot \beta_4 \cdot \beta_3 + \beta_3 \cdot \alpha_1 \cdot \alpha_2$$

$$x_2 = \beta_2 \cdot \beta_1 \cdot \beta_4 + \alpha_1 \cdot \alpha_2 \cdot \alpha_3 + \alpha_3 \cdot \beta_1 \cdot \beta_4 + \alpha_2 \cdot \alpha_3 \cdot \beta_4$$

$$x_3 = \alpha_2 \cdot \alpha_3 \cdot \alpha_4 + \beta_3 \cdot \beta_2 \cdot \beta_1 + \beta_2 \cdot \beta_1 \cdot \alpha_4 + \alpha_3 \cdot \alpha_4 \cdot \beta_1$$

$$x_4 = \beta_4 \cdot \beta_3 \cdot \beta_2 + \alpha_3 \cdot \alpha_4 \cdot \alpha_1 + \beta_2 \cdot \alpha_4 \cdot \alpha_1 + \beta_3 \cdot \beta_2 \cdot \alpha_1$$

$$E_1 = \frac{x_1}{x_1 + x_2 + x_3 + x_4}$$

$$E_2 = \frac{x_2}{x_1 + x_2 + x_3 + x_4}$$

$$E_3 = \frac{x_3}{x_1 + x_2 + x_3 + x_4}$$

$$E_4 = \frac{x_4}{x_1 + x_2 + x_3 + x_4}$$

$$z_{Na} = 1, \quad z_K = 1$$

$$J_{NaK,Na} = 3 \cdot (E_1 \cdot \alpha_3 - E_2 \cdot \beta_3)$$

$$J_{NaK,K} = 2 \cdot (E_4 \cdot \beta_1 - E_3 \cdot \alpha_1)$$

$$I_{NaK} = 30 \cdot \left(z_{Na} \cdot J_{NaK,Na} + z_K \cdot J_{NaK,K}\right)$$

Background Currents (I_{Nab}, I_{Cab}, I_{Kb}) and Sarcolemmal Calcium Pump Current (I_{pCa})

The formulations for I_{Nab}, I_{Cab}, I_{Kb}, and I_{pCa} were taken from the Hund-Decker-Rudy model[5, 6]. I_{Kb} represents small amplitude, rapidly activating K^+ current observed in the ventricle (I_{Kp}-like[7] or I_{Kur}-like[8] current). The amplitudes of these currents were reduced compared to values used by Decker et al[5]. These choices were made consistent with the following: 1) so that resting $[Na^+]_i$ would be similar to values shown in nonfailing human ventricle at 37 °C by Pieske et al.[9] at very slow pacing rates (0.25 Hz), 2) so that the resting $[Ca^{2+}]_i$ would be similar to values shown in nonfailing human ventricle at 37° C by Schmidt et al.[10], and 3) so that the generally lower major current conductances used to match human data in construction of this model would be properly balanced.

$$P_{Nab} = 3.75 \cdot 10^{-10} \text{ cm/s}, \quad z_{Na} = 1$$

$$I_{Nab} = P_{Nab} \cdot {z_{Na}}^2 \cdot \frac{VF^2}{RT} \cdot \frac{[Na^+]_i \cdot \exp\left(\frac{z_{Na}VF}{RT}\right) - [Na^+]_o}{\exp\left(\frac{z_{Na}VF}{RT}\right) - 1.0}$$

$$P_{Cab} = 2.5 \cdot 10^{-8} \text{ cm/s}, \quad \gamma_{Cai} = 1.0, \quad \gamma_{Cao} = 0.341, \quad z_{Ca} = 2$$

$$I_{Cab} = P_{Cab} \cdot {z_{Ca}}^2 \cdot \frac{VF^2}{RT} \cdot \frac{\gamma_{Cai} \cdot [Ca^{2+}]_i \cdot \exp\left(\frac{z_{Ca}VF}{RT}\right) - \gamma_{Cao} \cdot [Ca^{2+}]_o}{\exp\left(\frac{z_{Ca}VF}{RT}\right) - 1.0}$$

$$x_{Kb} = \frac{1}{1 + \exp\left(\frac{-(V - 14.48)}{18.34}\right)}$$
$$\overline{G_{Kb}} = 0.003\ \mathrm{mS}/\mu\mathrm{F}$$
$$I_{Kb} = \overline{G_{Kb}} \cdot x_{Kb} \cdot (V - E_K)$$
$$\overline{G_{pCa}} = 0.0005\ \mathrm{mS}/\mu\mathrm{F}$$
$$I_{pCa} = \overline{G_{pCa}} \cdot \frac{[Ca^{2+}]_i}{0.0005 + [Ca^{2+}]_i}$$

Voltage

$$C_m = 1.0\ \mu\mathrm{F}$$
$$\frac{dV_m}{dt} = -\frac{1}{C_m} \cdot (I_{Na} + I_{to} + I_{CaL} + I_{CaNa} + I_{CaK} + I_{Kr} + I_{Ks} + I_{K1} + I_{NaCa} + I_{NaK} + I_{Nab} + I_{Cab} + I_{Kb} + I_{pCa} + I_{stim})$$

Calcium/Calmodulin-Dependent Protein Kinase (CaMK)

The CaMK model is equivalent to that used in the Hund-Decker-Rudy dog model[5, 6]. We assumed that CaMK kinetics are similar in human and dog, in the absence of human ventricle specific measurements.

$$\alpha_{CaMK} = 0.05\ \mathrm{ms}^{-1}, \quad \beta_{CaMK} = 0.00068\ \mathrm{ms}^{-1}$$
$$CaMK_0 = 0.05, \quad K_{mCaM} = 0.0015\ \mathrm{mM}$$
$$CaMK_{bound} = CaMK_0 \cdot \frac{1 - CaMK_{trap}}{1 + \frac{K_{mCaM}}{[Ca^{2+}]_{ss}}}$$
$$CaMK_{active} = CaMK_{bound} + CaMK_{trap}$$
$$\frac{dCaMK_{trap}}{dt} = \alpha_{CaMK} \cdot CaMK_{bound} \cdot \left(CaMK_{bound} + CaMK_{trap}\right) - \beta_{CaMK} \cdot CaMK_{trap}$$

ORd Human Model Fluxes

Diffusion Fluxes ($J_{diff,Na}$, $J_{diff,Ca}$, $J_{diff,K}$)

$$\tau_{diff,Na} = \tau_{diff,K} = 2.0\ \mathrm{ms}, \quad \tau_{diff,Ca} = 0.2\ \mathrm{ms}$$
$$J_{diff,Na} = \frac{[Na^+]_{ss} - [Na^+]_i}{\tau_{diff,Na}}$$
$$J_{diff,Ca} = \frac{[Ca^{2+}]_{ss} - [Ca^{2+}]_i}{\tau_{diff,Ca}}$$
$$J_{diff,K} = \frac{[K^+]_{ss} - [K^+]_i}{\tau_{diff,K}}$$

The time constant for Na^+ and K^+ diffusion fluxes are larger than the time constant for Ca^{2+} diffusion flux. Physiologically, this amounts to reduced diffusivity for Na^+ and K^+ as they exit the subspace.

SR Calcium Release Flux, via Ryanodine Receptor (J_{rel})

Ca^{2+} release channels (ryanodine receptors, RyRs, formulation similar to that in Livshitz et al.[11]) have been split into two separate populations in this model according to CaMK phosphorylation state, based on observations in dog ventricle[12]. There is a non-phosphorylated release ($J_{rel,NP}$) and a CaMK phosphorylated release ($J_{rel,CaMK}$). When RyR channels are phosphorylated by CaMK, release amplitude is 1.25 times larger, and the decay time constant is 1.25 times longer. The proportion of the RyR population that behaves in the phosphorylated state is regulated by active CaMK.

$$\beta_\tau = 4.75\text{ ms}$$

$$\alpha_{rel} = 0.5 \cdot \beta_\tau$$

$$J_{rel,NP,\infty} = \frac{\alpha_{rel} \cdot (-I_{CaL})}{1 + \left(\frac{1.5}{[Ca^{2+}]_{jsr}}\right)^8}$$

$$\tau_{rel,NP} = \frac{\beta_\tau}{1 + \left(\frac{0.0123}{[Ca^{2+}]_{jsr}}\right)}, \tau_{rel,NP} \geq 0.001$$

$$\frac{dJ_{rel,NP}}{dt} = \frac{J_{rel,NP,\infty} - J_{rel,NP}}{\tau_{rel,NP}}$$

$$\beta_{\tau,CaMK} = 1.25 \cdot \beta_\tau$$

$$\alpha_{rel,CaMK} = 0.5 \cdot \beta_{\tau,CaMK}$$

$$J_{rel,CaMK,\infty} = \frac{\alpha_{rel,CaMK} \cdot (-I_{CaL})}{1 + \left(\frac{1.5}{[Ca^{2+}]_{jsr}}\right)^8}$$

$$\tau_{rel,CaMK} = \frac{\beta_{\tau,CaMK}}{1 + \left(\frac{0.0123}{[Ca^{2+}]_{jsr}}\right)}, \tau_{rel,CaMK} \geq 0.001$$

$$\frac{dJ_{rel,CaMK}}{dt} = \frac{J_{rel,CaMK,\infty} - J_{rel,CaMK}}{\tau_{rel,CaMK}}$$

$$K_{m,CaMK} = 0.15, \quad \emptyset_{rel,CaMK} = \frac{1}{1 + \frac{K_{m,CaMK}}{CaMK_{active}}}$$

$$J_{rel} = \left(1 - \emptyset_{rel,CaMK}\right) \cdot J_{rel,NP} + \emptyset_{rel,CaMK} \cdot J_{rel,CaMK}$$

Calcium Uptake via SERCA Pump (J_{up})

Ca^{2+} uptake channels (SERCA pumps) are phosphorylated by CaMK[13, 14]. Here, we used two separate Ca^{2+} uptake populations: those not-phosphorylated ($J_{up,NP}$) and those phosphorylated by CaMK ($J_{up,CaMK}$). Ca^{2+} leakage from the NSR was identical to the formulation used in the Hund-Decker-Rudy model. However, leak magnitude was reduced by ~10%.

$$J_{up,NP} = \frac{0.004375 \cdot [Ca^{2+}]_i}{0.00092 + [Ca^{2+}]_i}$$

$$\overline{\Delta K_{m,PLB}} = 0.00017\text{ mM}$$

$$\overline{\Delta J_{up,CaMK}} = 1.75$$

$$J_{up,CaMK} = \left(1 + \overline{\Delta J_{up,CaMK}}\right) \cdot \frac{0.004375 \cdot [Ca^{2+}]_i}{0.00092 - \overline{\Delta K_{m,PLB}} + [Ca^{2+}]_i}$$

$$K_{m,CaMK} = 0.15, \quad \emptyset_{up,CaMK} = \frac{1}{1 + \frac{K_{m,CaMK}}{CaMK_{active}}}$$

$$J_{leak} = \frac{0.0039375 \cdot [Ca^{2+}]_{nsr}}{15.0}$$

$$J_{up} = \left(1 - \emptyset_{up,CaMK}\right) \cdot J_{up,NP} + \emptyset_{up,CaMK} \cdot J_{up,CaMK} - J_{leak}$$

Calcium Translocation from NSR to JSR (J_{tr})

Sobie et al.[15] showed that Ca^{2+} spark recovery required 91 ms. This measurement informed our choice of 100 ms for translocation time constant (τ_{tr}).

$$\tau_{tr} = 100\ \text{ms}$$

$$J_{tr} = \frac{[Ca^{2+}]_{nsr} - [Ca^{2+}]_{jsr}}{\tau_{tr}}$$

ORd Human Model Concentrations and Buffers

In the absence of human ventricle specific measurements, we take Ca^{2+} buffering equations and kinetics from the Hund-Decker-Rudy model.

$$\overline{[CMDN]} = 0.05\ \text{mM}, \qquad K_{m,CMDN} = 0.00238\ \text{mM}$$

$$\overline{[TRPN]} = 0.07\ \text{mM}, \qquad K_{m,TRPN} = 0.0005\ \text{mM}$$

$$\overline{[BSR]} = 0.047\ \text{mM}, \qquad K_{m,BSR} = 0.00087\ \text{mM}$$

$$\overline{[BSL]} = 1.124\ \text{mM}, \qquad K_{m,BSL} = 0.0087\ \text{mM}$$

$$\overline{[CSQN]} = 10.0\ \text{mM}, \qquad K_{m,CSQN} = 0.8\ \text{mM}$$

$$\frac{d[Na^+]_i}{dt} = -\left(I_{Na} + I_{NaL} + 3 \cdot I_{NaCa,i} + 3 \cdot I_{NaK} + I_{Nab}\right) \cdot \frac{A_{cap}}{F \cdot v_{myo}} + J_{diff,Na} \cdot \frac{v_{ss}}{v_{myo}}$$

$$\frac{d[Na^+]_{ss}}{dt} = -\left(I_{CaNa} + 3 \cdot I_{NaCa,ss}\right) \cdot \frac{A_{cap}}{F \cdot v_{ss}} - J_{diff,Na}$$

$$\frac{d[K^+]_i}{dt} = -(I_{to} + I_{Kr} + I_{Ks} + I_{K1} + I_{Kur} + I_{stim} - 2 \cdot I_{NaK}) \cdot \frac{A_{cap}}{F \cdot v_{myo}} + J_{diff,K} \cdot \frac{v_{ss}}{v_{myo}}$$

$$\frac{d[K^+]_{ss}}{dt} = -I_{CaK} \cdot \frac{A_{cap}}{F \cdot v_{ss}} - J_{diff,K}$$

$$\beta_{Cai} = \frac{1}{1 + \frac{\overline{[CMDN]} \cdot K_{m,CMDN}}{\left(K_{m,CMDN} + [Ca^{2+}]_i\right)^2} + \frac{\overline{[TRPN]} \cdot K_{m,TRPN}}{\left(K_{m,TRPN} + [Ca^{2+}]_i\right)^2}}$$

$$\frac{d[Ca^{2+}]_i}{dt} = \beta_{Cai} \cdot \left(-\left(I_{pCa} + I_{Cab} - 2 \cdot I_{NaCa,i}\right) \cdot \frac{A_{cap}}{2 \cdot F \cdot v_{myo}} - J_{up} \cdot \frac{v_{nsr}}{v_{myo}} + J_{diff,Ca} \cdot \frac{v_{ss}}{v_{myo}}\right)$$

$$\beta_{Cass} = \frac{1}{1 + \frac{\overline{[BSR]} \cdot K_{m,BSR}}{\left(K_{m,BSR} + [Ca^{2+}]_{ss}\right)^2} + \frac{\overline{[BSL]} \cdot K_{m,BSL}}{\left(K_{m,BSL} + [Ca^{2+}]_{ss}\right)^2}}$$

$$\frac{d[Ca^{2+}]_{ss}}{dt} = \beta_{Cass} \cdot \left(-\left(I_{CaL} - 2 \cdot I_{NaCa,ss}\right) \cdot \frac{A_{cap}}{2 \cdot F \cdot v_{ss}} + J_{rel} \cdot \frac{v_{jsr}}{v_{ss}} - J_{diff,Ca}\right)$$

$$\frac{d[Ca^{2+}]_{nsr}}{dt} = J_{up} - J_{tr} \cdot \frac{v_{jsr}}{v_{nsr}}$$

$$\beta_{Cajsr} = \frac{1}{1 + \frac{\overline{[CSQN]} \cdot K_{m,CSQN}}{\left(K_{m,CSQN} + [Ca^{2+}]_{jsr}\right)^2}}$$

$$\frac{d[Ca^{2+}]_{jsr}}{dt} = \beta_{Cajsr} \cdot (J_{tr} - J_{rel})$$

ORd Human Model Transmural Heterogeneity

	epi/endo	M/endo
G_{NaL}	0.6	1
G_{to}	4.0	4.0
P_{Ca}, P_{CaNa}, P_{CaK}	1.2	2.5
G_{Kr}	1.3	0.8
G_{Ks}	1.4	1
G_{K1}	1.2	1.3
$G_{NaCa,i}$, $G_{NaCa,ss}$	1.1	1.4
G_{NaK}	0.9	0.7
G_{Kb}	0.6	1
$J_{rel,NP,\infty}$, $J_{rel,CaMK,\infty}$	1	1.7
$J_{up,NP}$, $J_{up,CaMK}$	1.3	1
$\overline{[CMDN]}$	1.3	1

Scaling Factors for Model Implementation of Transmural Heterogeneity

Formulation changes to account for epi I_{to} differences are:

$$\delta_{epi} = 1.0 - \frac{0.95}{1.0 + \exp\left(\frac{V + 70.0}{5.0}\right)}$$

$$\tau_{i,epi,fast} = \tau_{i,fast} \cdot \delta_{epi}$$

$$\tau_{i,epi,slow} = \tau_{i,slow} \cdot \delta_{epi}$$

Computational Methodology

Hardware and Software

For simulation of the basic human model, we used custom code developed and run using Microsoft Visual C++ 2008 Express Edition on a Windows Vista Dell desktop PC, with an Intel Core 2 Quad processor. Integration was performed as described below (Rapid Integration). We also used custom C++ code run on an array of Dell cluster nodes with 64-bit Intel Xeon processors, running Linux and Sun Microsystems Grid Engine. Execution scripts were written in Python. A fixed time step of 0.01 ms was applied, and the Rush-Larsen Method[16] was used. All simulations were paced to true steady state[17], unless otherwise noted.

Validation and fitting of individual model components (i.e. time constants, steady state curves) was performed using custom code written in Matlab 2009a running on a Windows Vista Dell desktop PC, with an Intel Core 2 Quad processor. Automated parameter estimation used a sum of least squares objective function, minimized by Matlab functions "fmincon", "ga", and "lsqcurvefit" (interior reflexive Newton's Method for "fmincon" and "lsqcurvefit", genetic algorithm for "ga"). See Matlab documentation for details and references. We used the parallel implementation of "fmincon" and "ga" by opening a matlabpool (size 4). Manual parameter estimation was also used, where minimization was by simple guess and check.

Rapid Integration

The Rush-Larsen Method[16], applied by Victorri et al.[18], relies on the assumption that during sufficiently small time intervals, a system of differential algebraic equations becomes effectively uncoupled. One can then readily solve uncoupled differential equations one-by-one to obtain expressions for time evolution of state variables.

Here, identification of sufficiently small time intervals (dt) was determined by comparison to gold standard simulations with fixed dt = 0.005 ms. We match the gold standard when we apply the following rules:

1) dt = 0.005 ms from the start of the stimulus until 25 ms thereafter
2) maximum allowed dt = 1.0 ms
3) dt was adjusted dynamically with changes in membrane voltage, as described in LR1[19]:
    a. $\text{if } \Delta V \leq 0.2\text{ mV}, dt = 0.8 \cdot \frac{dV}{dt}$
    b. $\text{if } \Delta V \geq 0.8\text{ mV}, dt = 0.2 \cdot \frac{dV}{dt}$
        i. while $\Delta V \geq 0.8$ mV, dt is reduced tenfold until the condition, $\Delta V < 0.8$ mV, is met (minimum dt = 0.005 ms)

Equations for updating gates (e.g. generic gate, s)

$$s = s_\infty - (s_\infty - s) \cdot \exp\left(\frac{-dt}{\tau_s}\right)$$

Equations for updating the n gate, $J_{rel,NP}$, and $J_{rel,CaMK}$

$$n = \alpha_n \cdot \frac{k_{+2,n}}{k_{-2,n}} - \left(\alpha_n \cdot \frac{k_{+2,n}}{k_{-2,n}} - n\right) \cdot \exp(-k_{-2,n} \cdot dt)$$

$$J_{rel,NP} = J_{rel,NP,\infty} - (J_{rel,NP,\infty} - J_{rel,NP}) \cdot \exp\left(\frac{-dt}{\tau_{rel,NP}}\right)$$

$$J_{rel,CaMK} = J_{rel,CaMK,\infty} - (J_{rel,CaMK,\infty} - J_{rel,CaMK}) \cdot \exp\left(\frac{-dt}{\tau_{rel,CaMK}}\right)$$

The Forward Euler Method was applied to update membrane voltage, concentrations, and $CaMK_{trap}$ at each time step.

Using the above method, it took less than one minute of runtime to pace the model to true and accurate steady state at 1 Hz (Microsoft Visual C++ 2008 Express Edition on a Windows Vista Dell desktop PC, with a 2.83 GHz Intel Core 2 Quad processor).

Additional Details for Currents

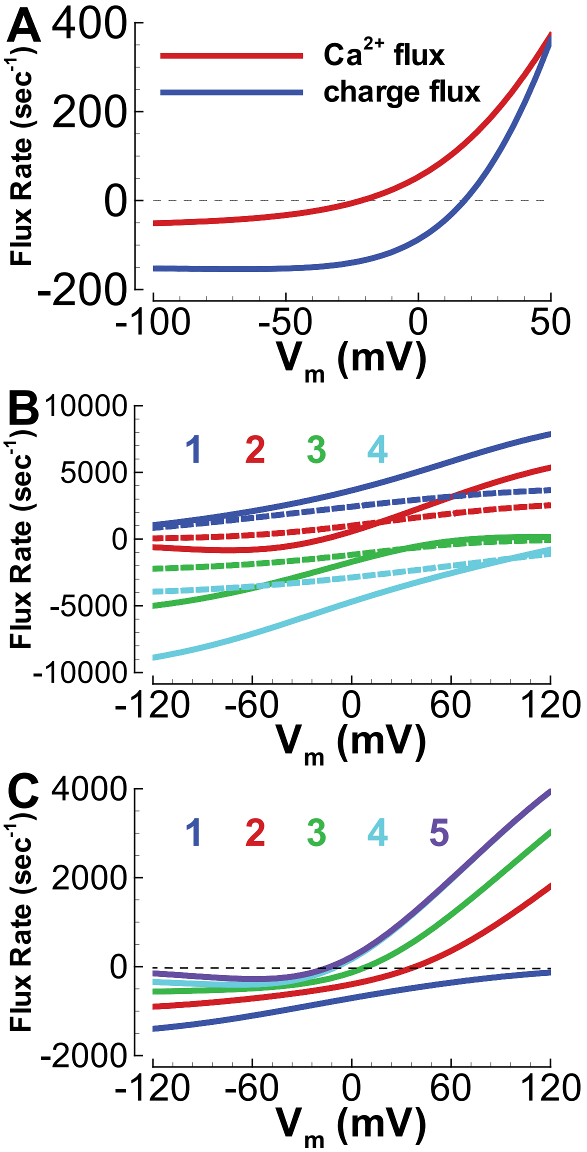

Figure S1. Human I_{NaCa} model faithfully reproduces Kang and Hilgemann[20] observations. Compare Figures 3C, 4A, 4C from Kang and Hilgemann[20] with the simulations from our human I_{NaCa} model, presented here (same protocols were used). A) Charge flux reversal potential is more depolarized than Ca^{2+} flux, a feature of this model which includes the observed Na^+ leak mode. B) Charge flux (solid lines) and Ca^{2+} flux (dashed lines) versus voltage, for a variety of substrates (in mM, condition 1: $[Na^+]_o$=0 , $[Na^+]_i$=40, $[Ca^{2+}]_o$=4, $[Ca^{2+}]_i$=0; condition 2: $[Na^+]_o$=120, $[Na^+]_i$=40, $[Ca^{2+}]_o$=4, $[Ca^{2+}]_i$=0; condition 3: $[Na^+]_o$=120, $[Na^+]_i$=40, $[Ca^{2+}]_o$=0, $[Ca^{2+}]_i$=0.1; condition 4: $[Na^+]_o$=120, $[Na^+]_i$=4, $[Ca^{2+}]_o$=0, $[Ca^{2+}]_i$=0.1). C) Reversal potential is sensitive to ionic substrate (in mM, $[Na^+]_o$=100, $[Ca^{2+}]_o$=1.2, $[Ca^{2+}]_i$=0.0005, condition 1: $[Na^+]_i$=0; condition 2: $[Na^+]_i$=5; condition 3: $[Na^+]_i$=10; condition 4: $[Na^+]_i$=20; condition 5: $[Na^+]_i$=20, $[Ca^{2+}]_i$=0).

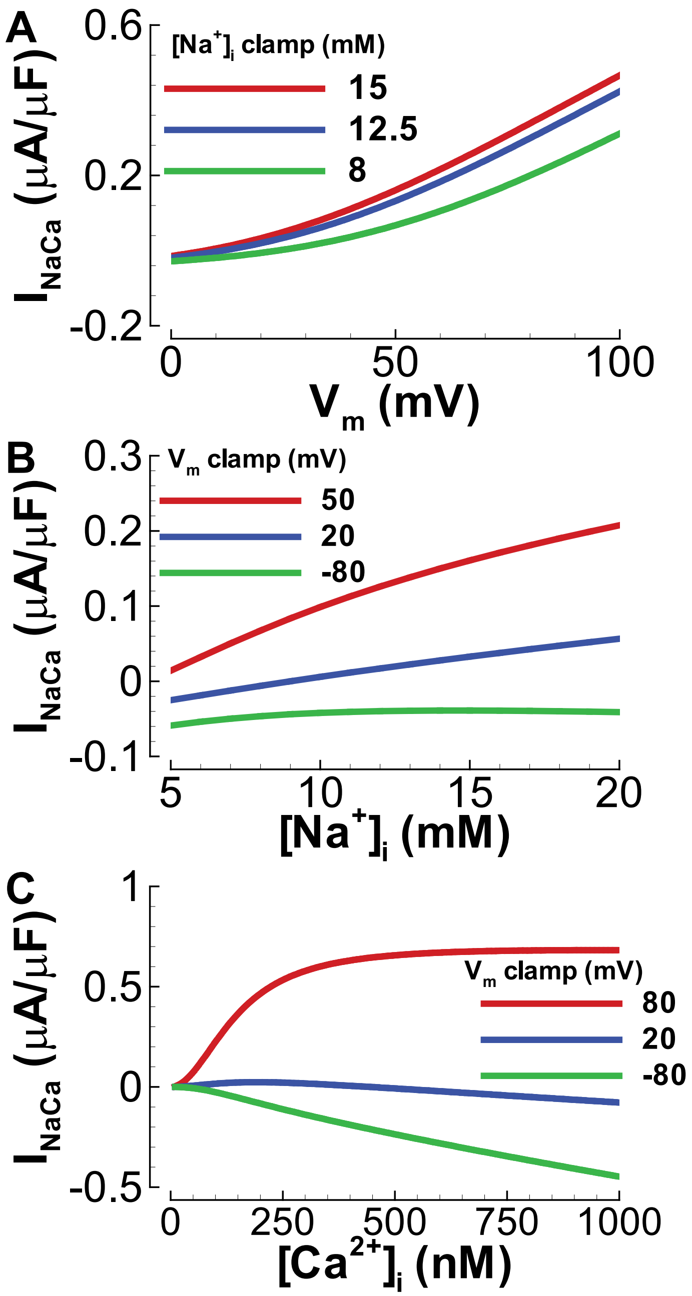

Figure S2. Human I_{NaCa} model faithfully reproduces observations of Weber et al.[21]. Compare Weber et al.[21], their Figure 2, with simulations from our human I_{NaCa} model, shown here (same protocols were used). A) Voltage dependence of I_{NaCa} under different intracellular Na^+ clamp conditions. B) Intracellular Na^+ dependence of I_{NaCa} under different voltage clamp conditions. C) Intracellular Ca^{2+} dependence of I_{NaCa} under different voltage clamp conditions. The model incorporates Weber's "allosteric activation", seen at depolarized voltages.

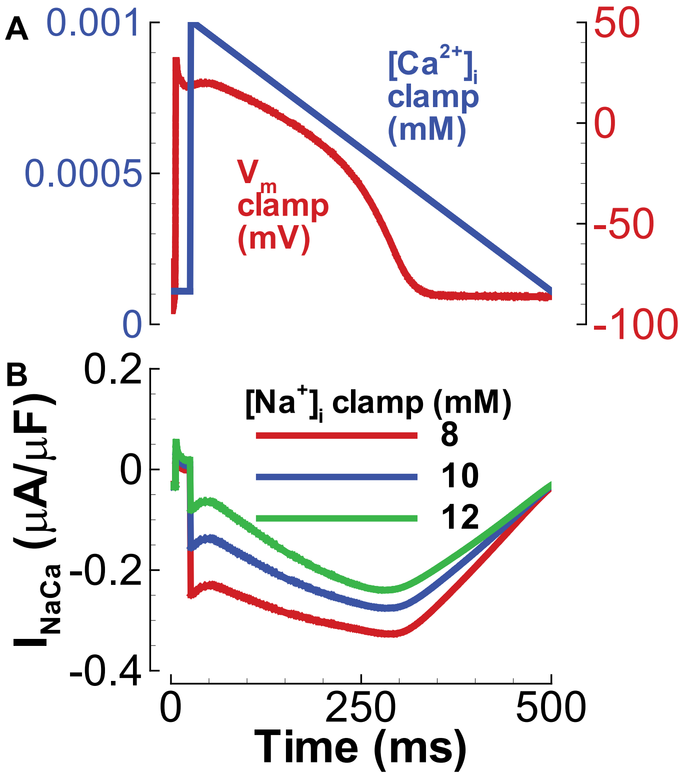

Figure S3. Human I_{NaCa} model faithfully reproduces Weber et al.[21] intracellular Na^+ dependence under AP and Ca^{2+} clamp conditions. Compare Weber et al.[21] Figure 6A with simulations from our I_{NaCa} model, shown here (same protocols were used). A) Clamped Ca^{2+} transient (similar to Weber et al.[21], increasing instantaneously from 0.01 to 0.1 μM, and decaying over 500 ms), and action potential waveform measured in undiseased human ventricular myocytes (see Methods section of main text for details). B) Due to depolarization, exchange current was outward, briefly, until $[Ca^{2+}]_i$ rose. When $[Na^+]_i$ was relatively low, maximal inward exchange current increased, as Weber showed.

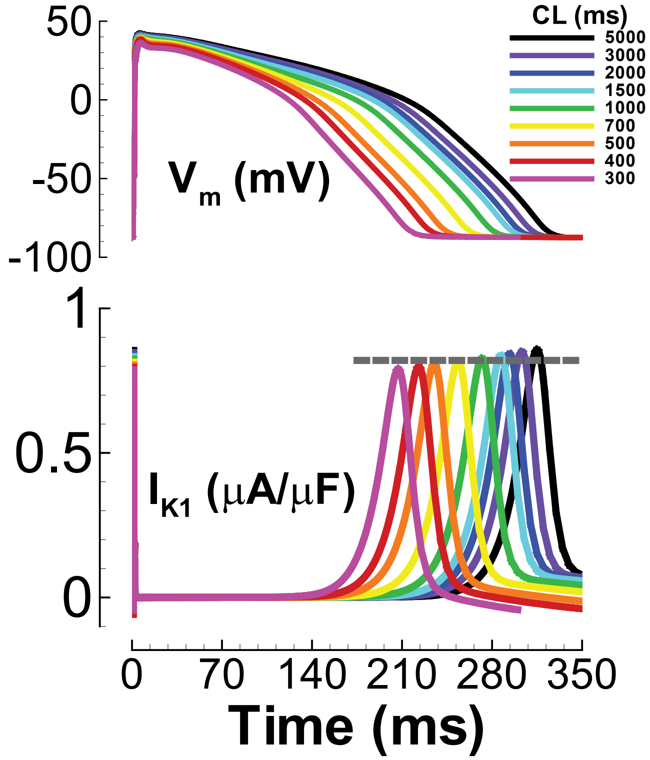

Figure S4. I_{K1} shows voltage dependence, but not rate dependence. Top panel: Simulated action potentials, paced at different cycle lengths. Bottom panel: I_{K1} in the model, at the different pacing rates. Note that the peak current reached was largely rate independent, as was shown by Jost et al.[22] *in undiseased human ventricle experiments.*

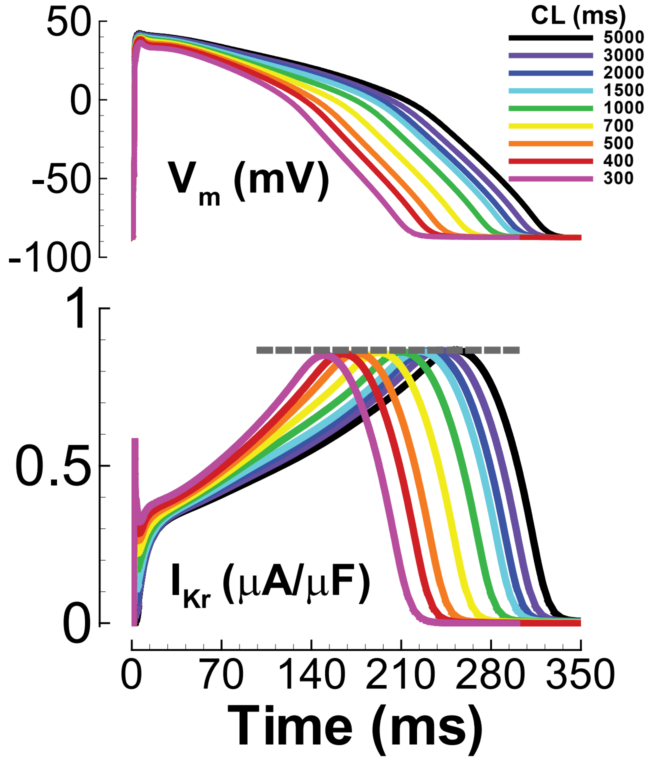

Figure S5. I_{Kr} shows voltage dependence, but not rate dependence. Top panel: Simulated action potentials, paced at different cycle lengths. Bottom panel: I_{Kr} in the model, at the different pacing rates. Note that the peak current reached was rate independent, as was shown by Jost et al.[22] *in undiseased human ventricle experiments.*

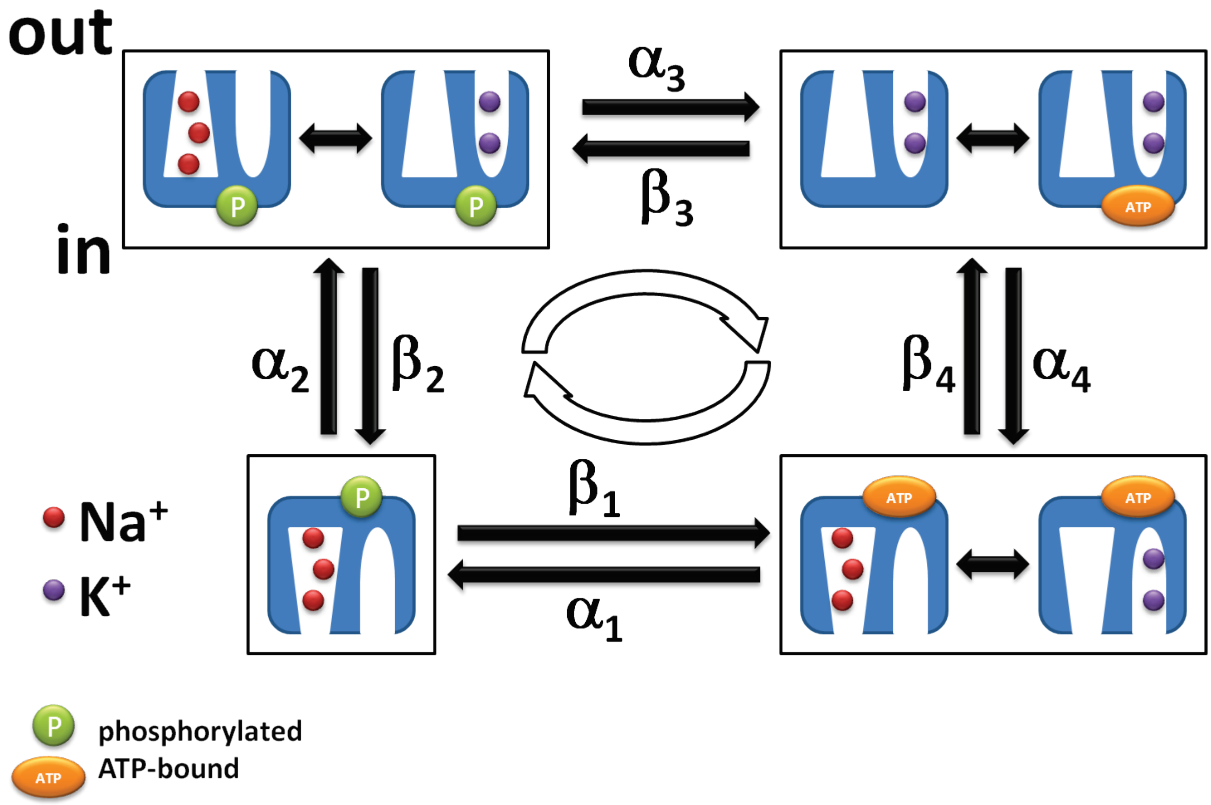

Figure S6. Schematic diagram of the human I_{NaK} model, modified from Smith and Crampin[23]*. There are four distinct enzymatic states, with lumped substates where non-rate limiting transitions were assumed to be in rapid equilibrium. Forward pump function is clockwise cycling. From this diagram, we formulated equations for the current using the King-Altmann method*[24]*.*

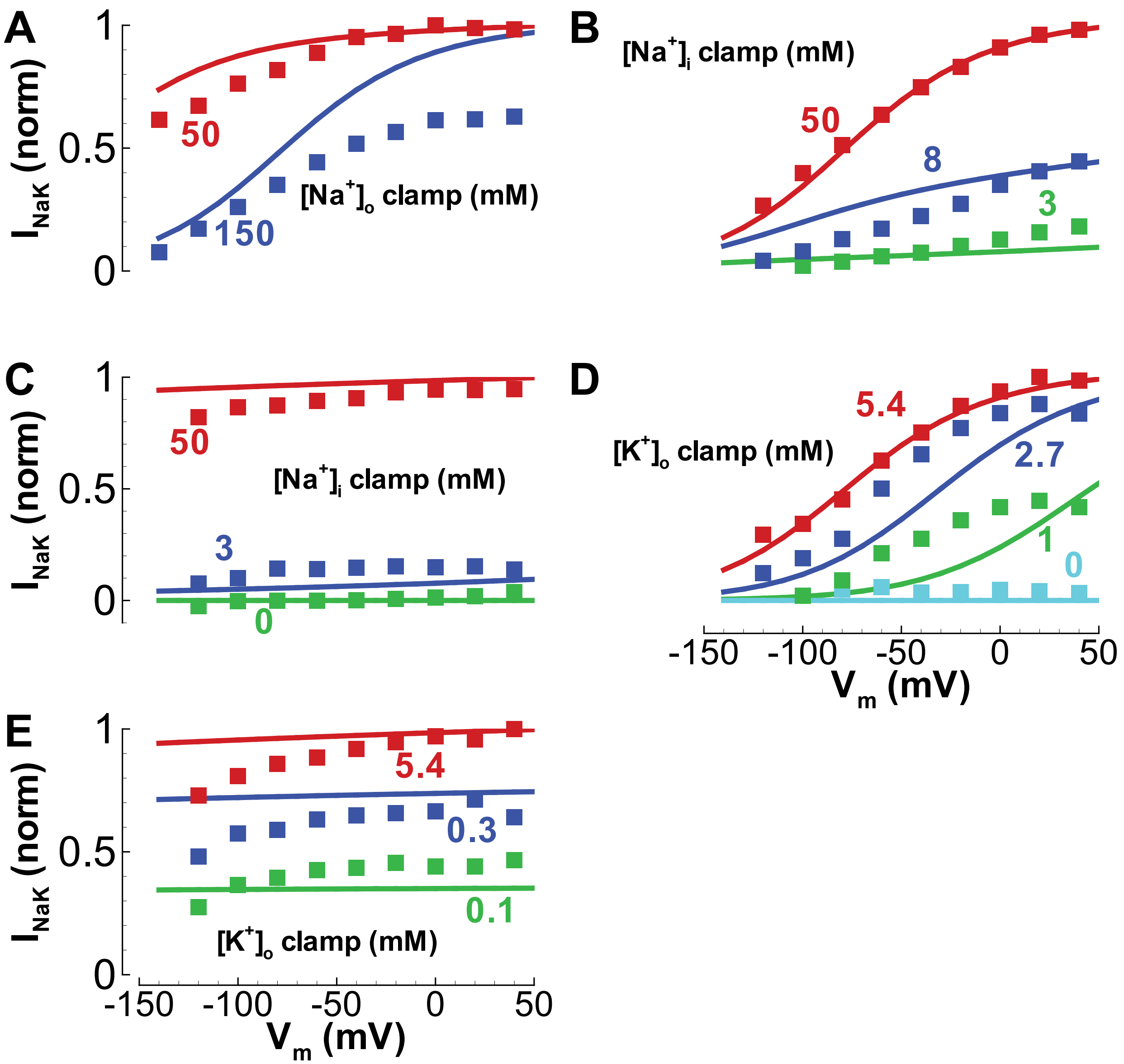

Figure S7. Human I_{NaK} model faithfully reproduces major observations of Nakao and Gadsby[25]. Data (squares) are from Nakao and Gadsby[25], their Figures 2A, 4C, 7A, 9B, 10B. I_{NaK} model simulations are solid lines (same protocols used). The model is not a perfect match to these data, measured in guinea pig ventricle, but the basic voltage and concentration dependencies are duplicated, demonstrating that the model is dynamically and mechanistically correct. A) Voltage dependence of I_{NaK} under different extracellular Na^+ clamp conditions. B) Voltage dependence of I_{NaK} under different intracellular Na^+ clamp conditions, and $[Na^+]_o$=150 mM. C) Voltage dependence of I_{NaK} under different intracellular Na^+ clamp conditions, and $[Na^+]_o$=0 mM. D) Voltage dependence of I_{NaK} under different extracellular K^+ clamp conditions, and $[Na^+]_o$=150 mM. E) Voltage dependence of I_{NaK} under different extracellular K^+ clamp conditions, and $[Na^+]_o$=0 mM.

We used microelectrode action potentials, measured in the undiseased human ventricle at 37 °C, for model validation. Since these data were measured in a multicellular preparation, the experimental protocol was simulated in a 1-dimensional multicellular fiber[5]. In Decker et al.[5], the subtle but complex differences in APD adaptation between single cell and a multicellular fiber were investigated. We used the conduction equations of Decker et al. (-200 μA/μF, 1 ms stimulus delivered to fiber end, zero flux boundary conditions), and measured the model APD at the 50th cell in a 100-cell homogeneous endocardial strand. Fiber results were similar to single cell results, as in Decker et al, and match the experimental data. Conduction velocity was 45 cm/sec at 1 Hz pacing, consistent with available (canine) experiments[26].

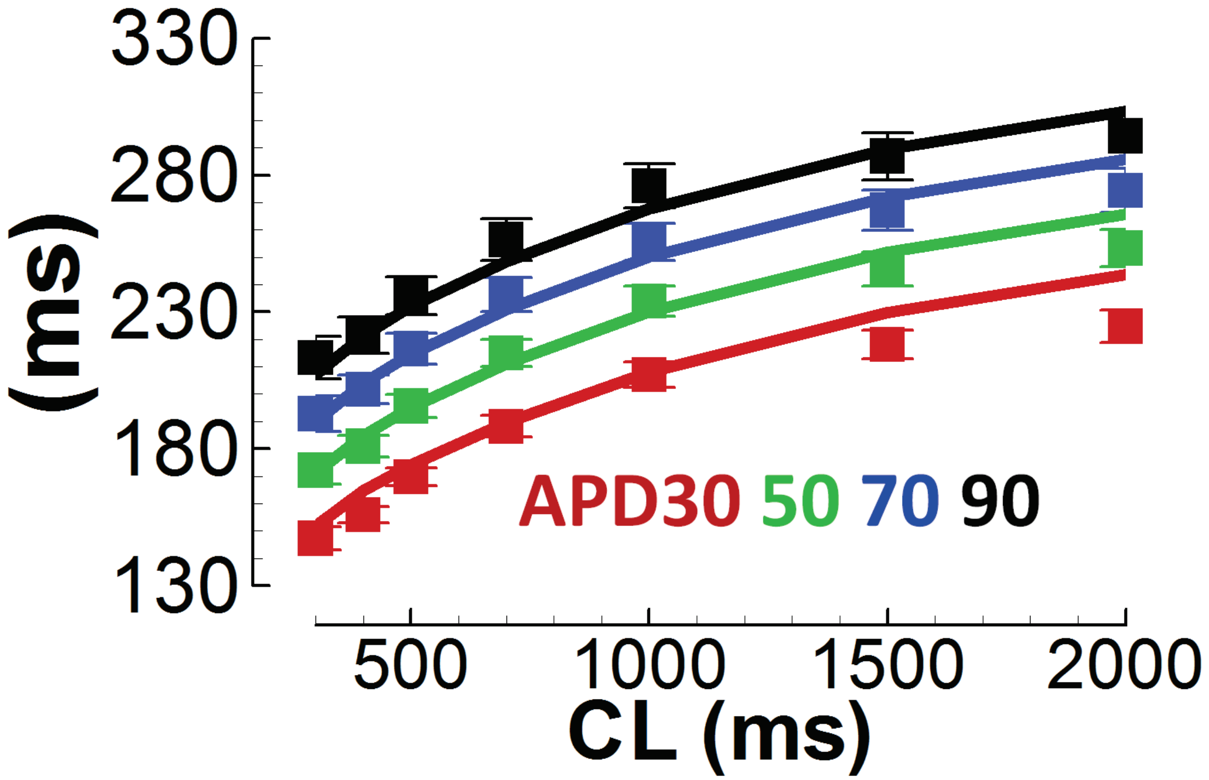

Figure S8. Multicellular strand simulations compared to measurements. APD30, APD50, APD70, and APD90 shown in red, green, blue, and black, respectively. Squares are experimentally measured human endocardial action potentials, at 37°C, N=140. Solid lines are simulation results from the 50th cell in a 100-cell strand (zero flux boundary).

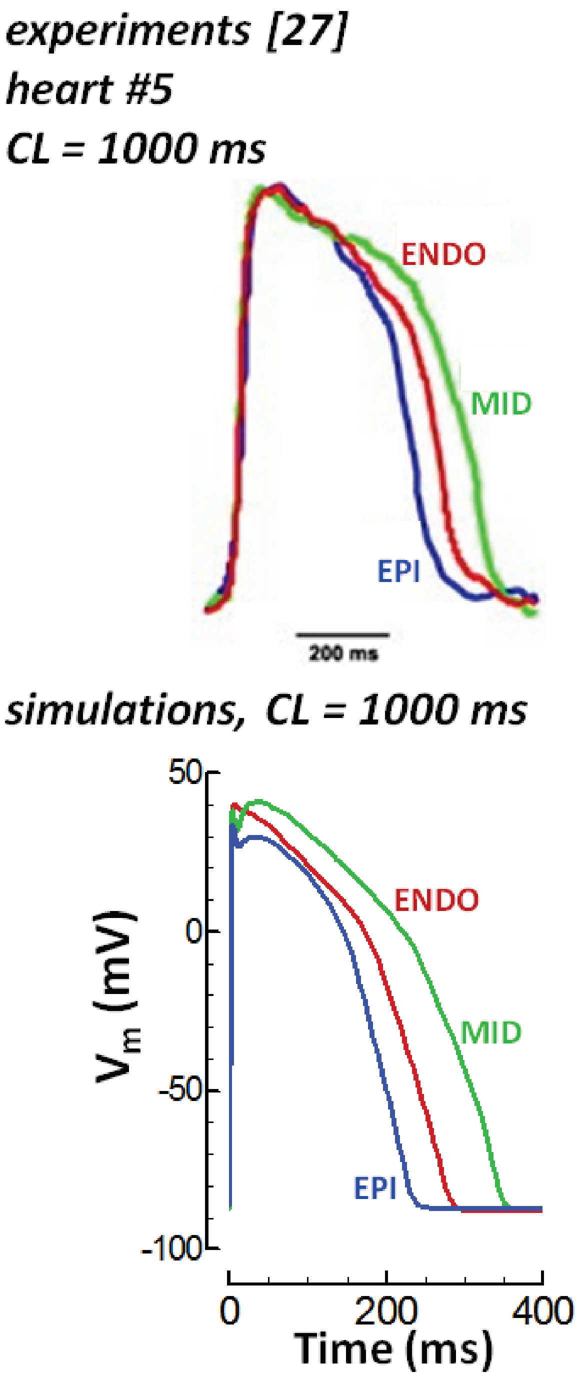

Figure S9. Experiments (top) are from an undiseased human heart (heart #5, male, age 20, death from Tylenol overdose), measured by Glukhov et al.[27], reproduced with permission. Simulations are below. Cell types are color coded and labeled. CL = 1000 ms.

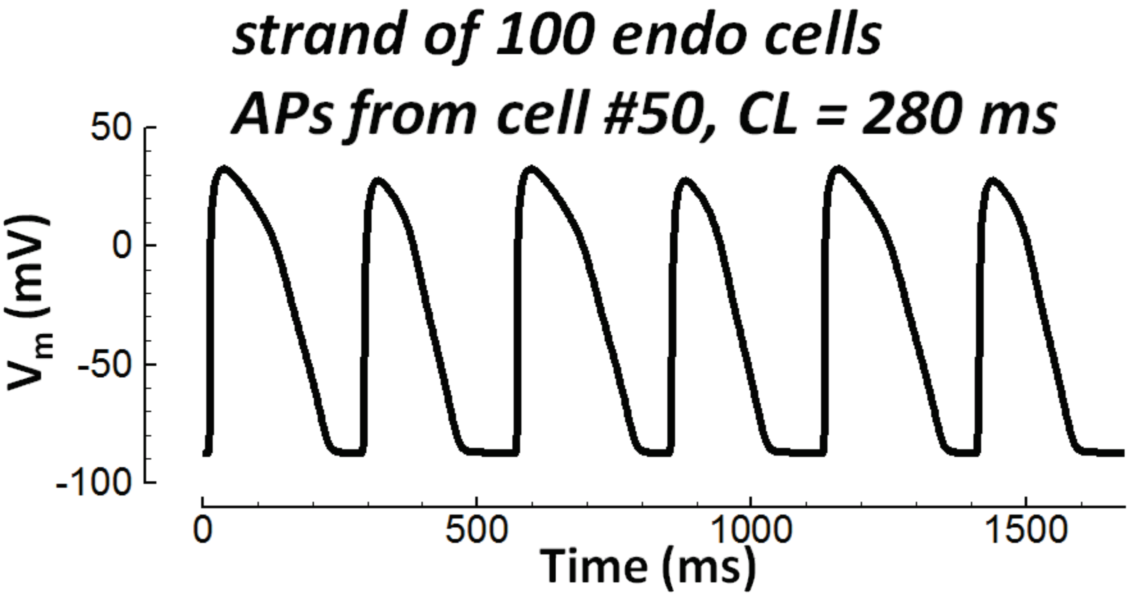

Figure S10. A strand composed of 100 endo cells was paced at CL = 280 ms until steady state was reached. Beat to beat APD alternans were evident at the central cell (#50, isolated from edge effects).

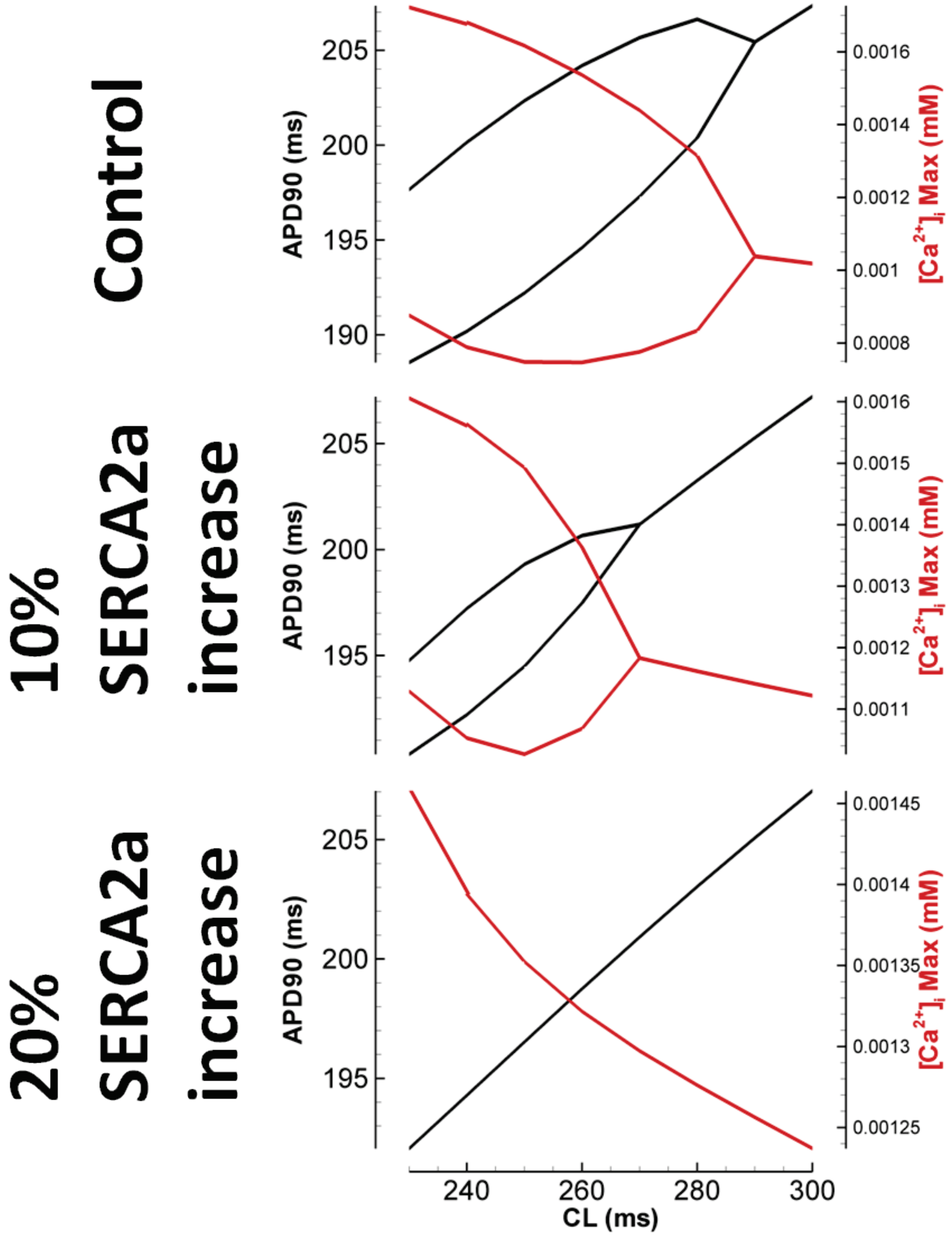

Figure S11. Alternans were eliminated by upregulation of SERCA2a (J_{up} in the model), as in experiments by Cutler et al.[28]. APD90 is on the left axes in black. Peak intracellular Ca^{2+} concentration ($[Ca^{2+}]_i$ Max) is on the right axes in red. From top to bottom, J_{up} was increased from control by 10, and 20 %. For 10% increase in J_{up}, the alternans bifurcation shifted to faster rates. For 20% increase, the bifurcation was eliminated.

We kept Cl^- concentration constant, since the ORd model does not systematically include Cl^- handling (20 mM intracellular and 100 mM extracellular, as in Decker et al.[5]). Otherwise, all Crampin and Smith equations were included exactly as described[29]. The I_{NaK} formulation we used, based on Smith and Crampin[23], includes pH dependence. The simulations below allow I_{NaK} to respond dynamically to pH.

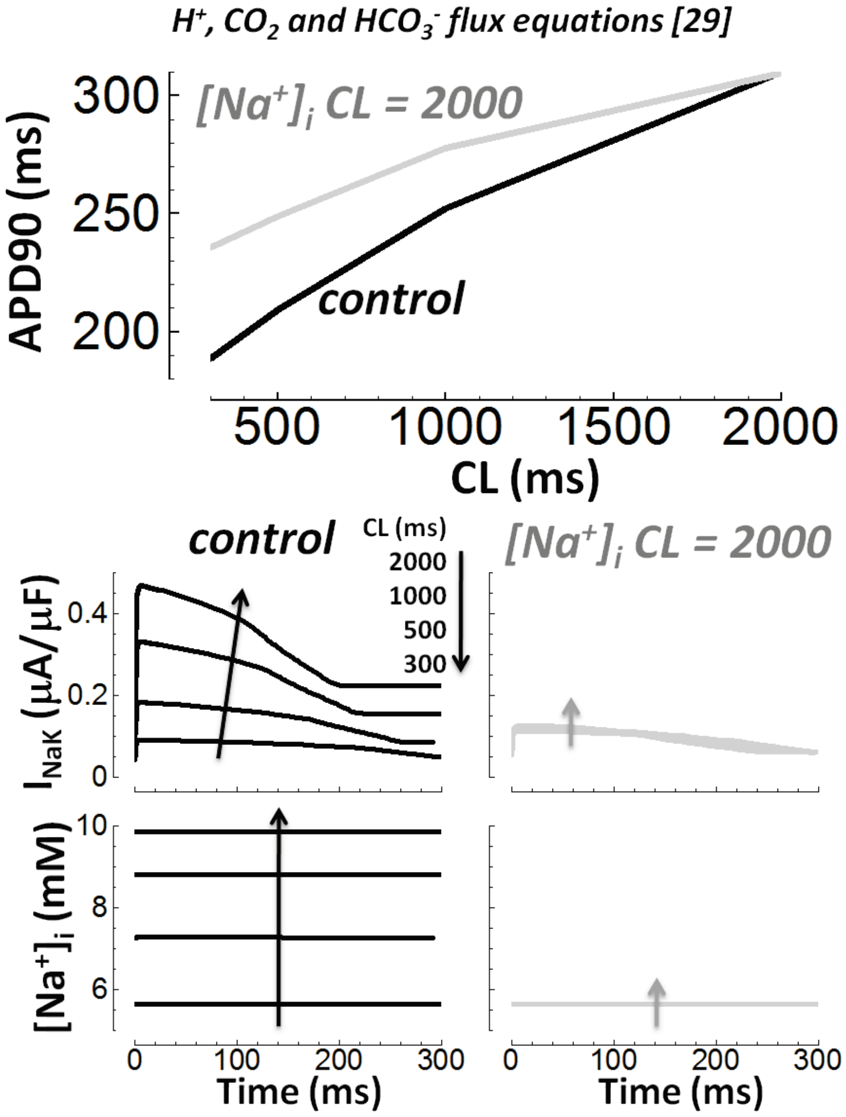

Figure S12. H^+, CO_2 and HCO_3^- fluxes did not change the relationship between Na^+ accumulation, I_{NaK} and APD rate dependence. Top) APD90 rate dependence with incorporation of the Crampin and Smith equations[29] (black line). When intracellular Na^+ concentration ($[Na^+]_i$) was artificially kept low, at the CL = 2000 ms value, the ability of the APD to shorten at fast rates was substantially reduced (gray line). Bottom) Left) As pacing rate increased (indicated by arrows) I_{NaK} increased due to $[Na^+]_i$ accumulation. Right) However, when $[Na^+]_i$ was clamped at the low CL = 2000 ms value, I_{NaK} was rate independent. This hampered APD shortening at fast pacing rates.

Protein forming the β-subunit of I_{Ks}, KCNE1 was measured to be transmurally heterogeneous in the undiseased human ventricle[30]. Western blots showed about two-fold greater intensity for KCNE1 in M-cells compared to epi cells. Considering that KCNE1:KCNQ1 stoichiometry is variable[31], and that the presence of KCNE1 slows I_{Ks} activation by about five fold and increases I_{Ks} conductance by about five fold, we simulated the effect of KCNE1 transmural heterogeneity on I_{Ks} and the AP. Thus, for heterogeneous KCNE1 simulations in M-cells, I_{Ks} activation was five times slower and conductance was five times greater than in the control M-cell. For epi cells, activation was five times faster, and conductance was five times smaller than in the control epi cell. These conditions are exaggerated (five fold changes compared KNCE1 overabundance to total KCNE1 absence[32]), showing possible KCNE1 transmural heterogeneity effects in the extreme. As shown, even for the extreme case there was little effect on I_{Ks} or especially on the AP.

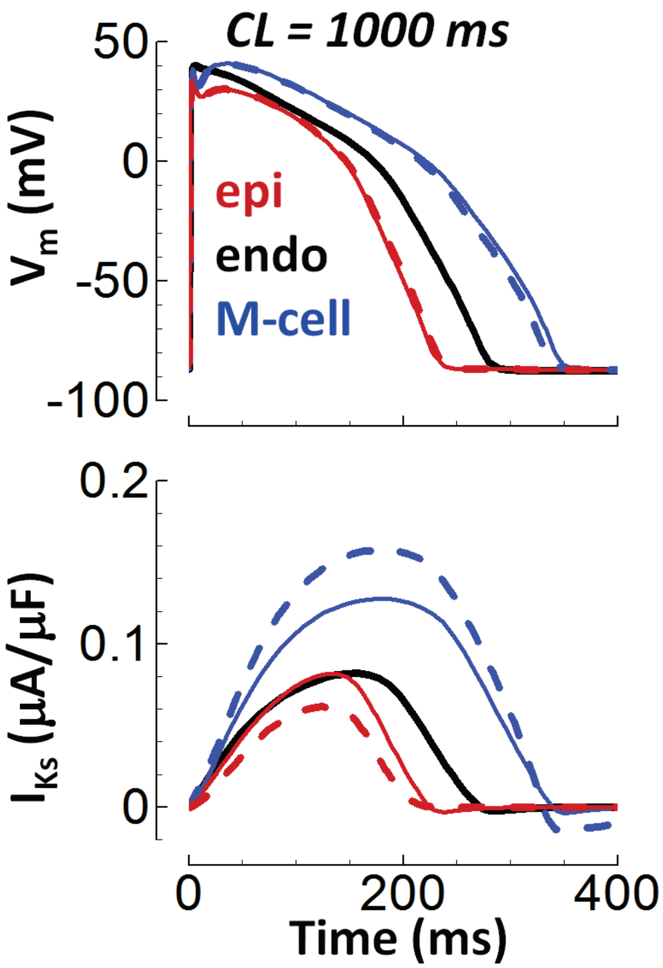

Figure S13. Transmural heterogeneity of KCNE1 β-subunit had minimal effect on transmural heterogeneity of I_{Ks} and the AP. Results for control conditions are solid lines (black is endo, blue is M-cell, red is epi). Dashed lines show the effect of the KCNE1 heterogeneity. Top) AP. Bottom) I_{Ks}.

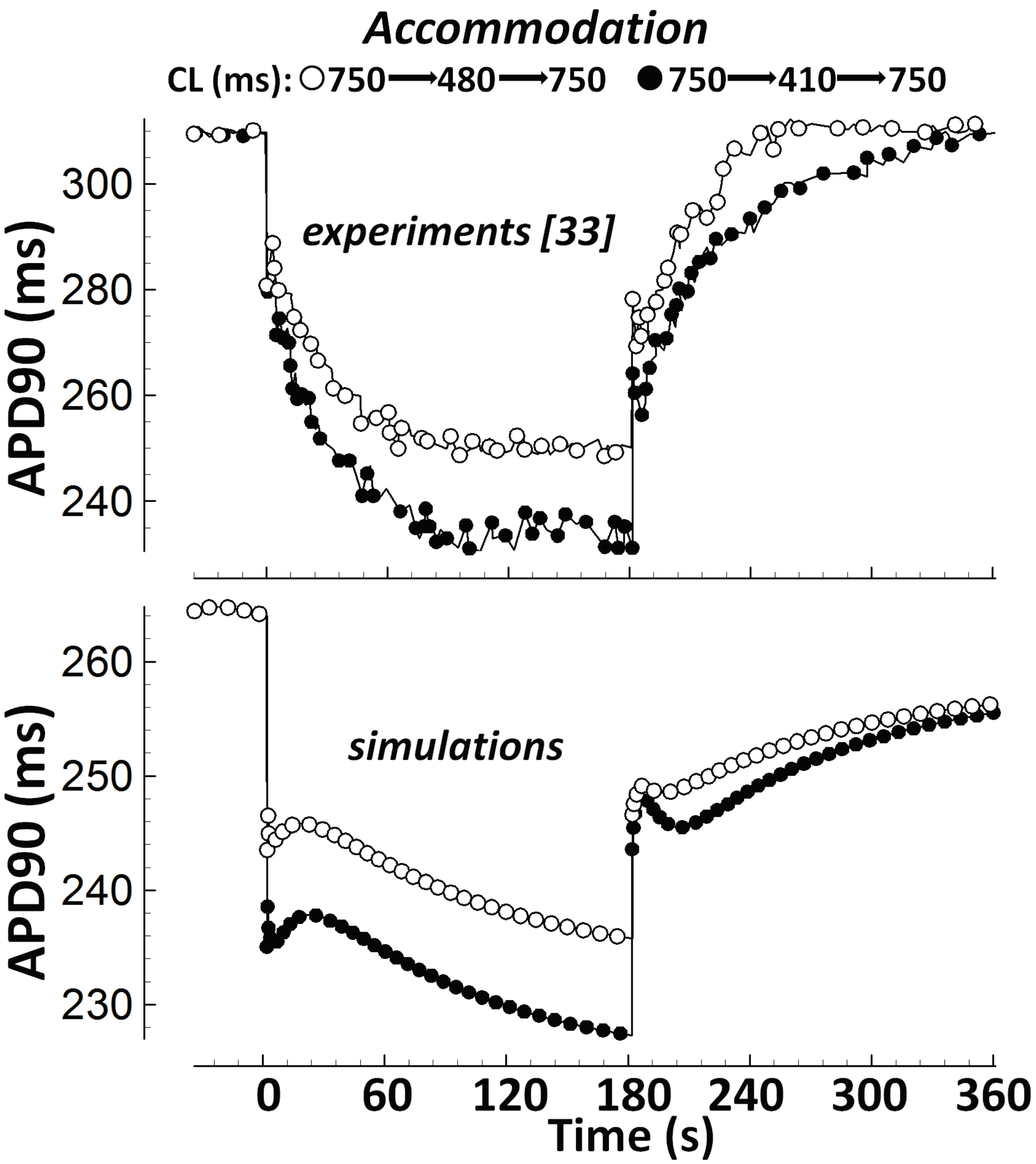

Figure S14. APD90 Accommodation. At time = 0 seconds, pacing CL was abruptly reduced. At time = 180 seconds, the pacing CL was abruptly increased to its original value. CL change from 750 to 480 ms is shown with white circles. Black circles show CL change from 750 to 410 ms. Experiments (top) are from in vivo nonfailing human hearts, measured by Franz et al.[33]. Simulations are below.

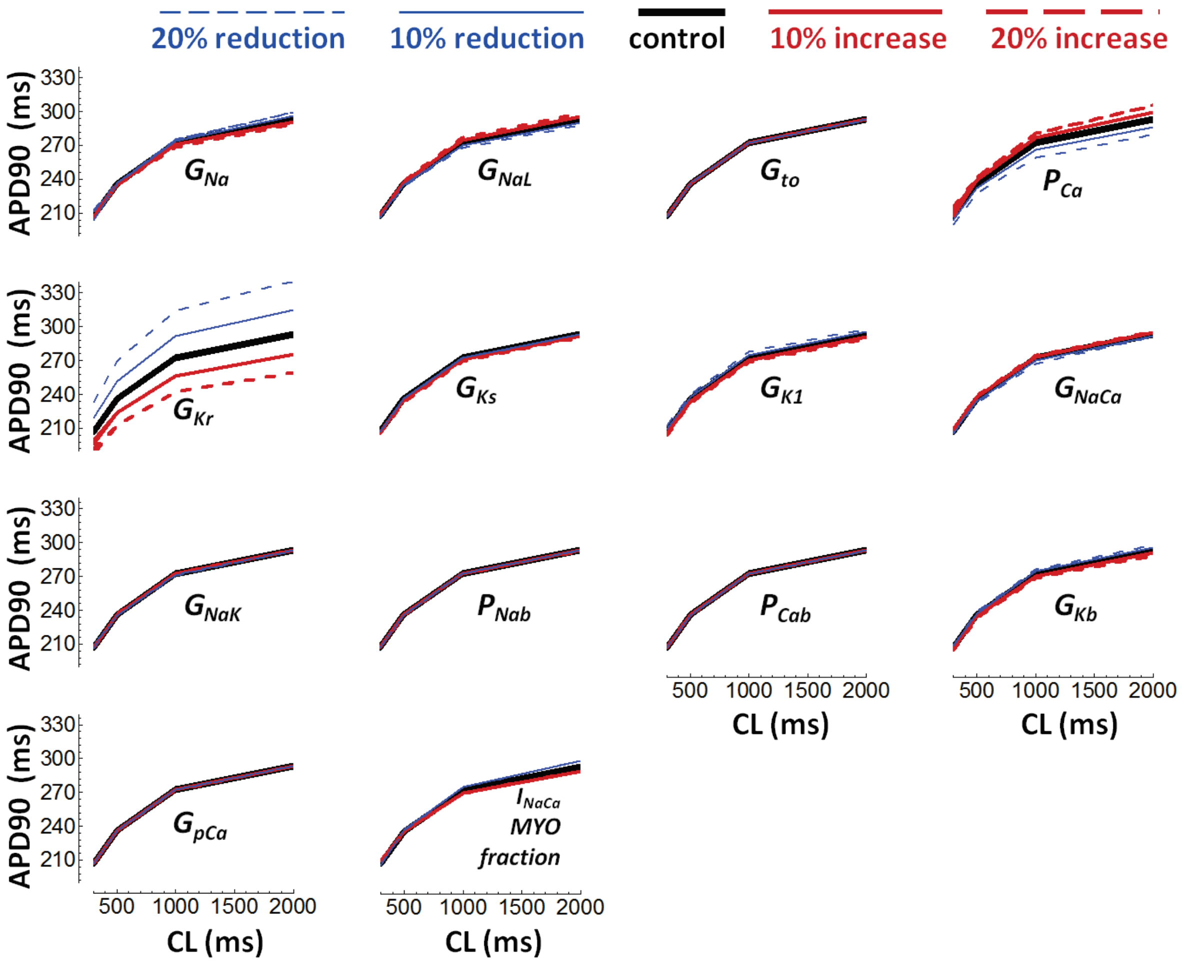

Figure S15. Sensitivity of steady state APD90 rate dependence to variations in current conductances and to the fraction of I_{NaCa} in the myoplasm (80% in the control case). The control case is shown with the thick black line. Parameter reductions are in blue (20% dashed blue, 10% solid blue). Parameter increases are shown in red (10% solid red, 20% dashed red).

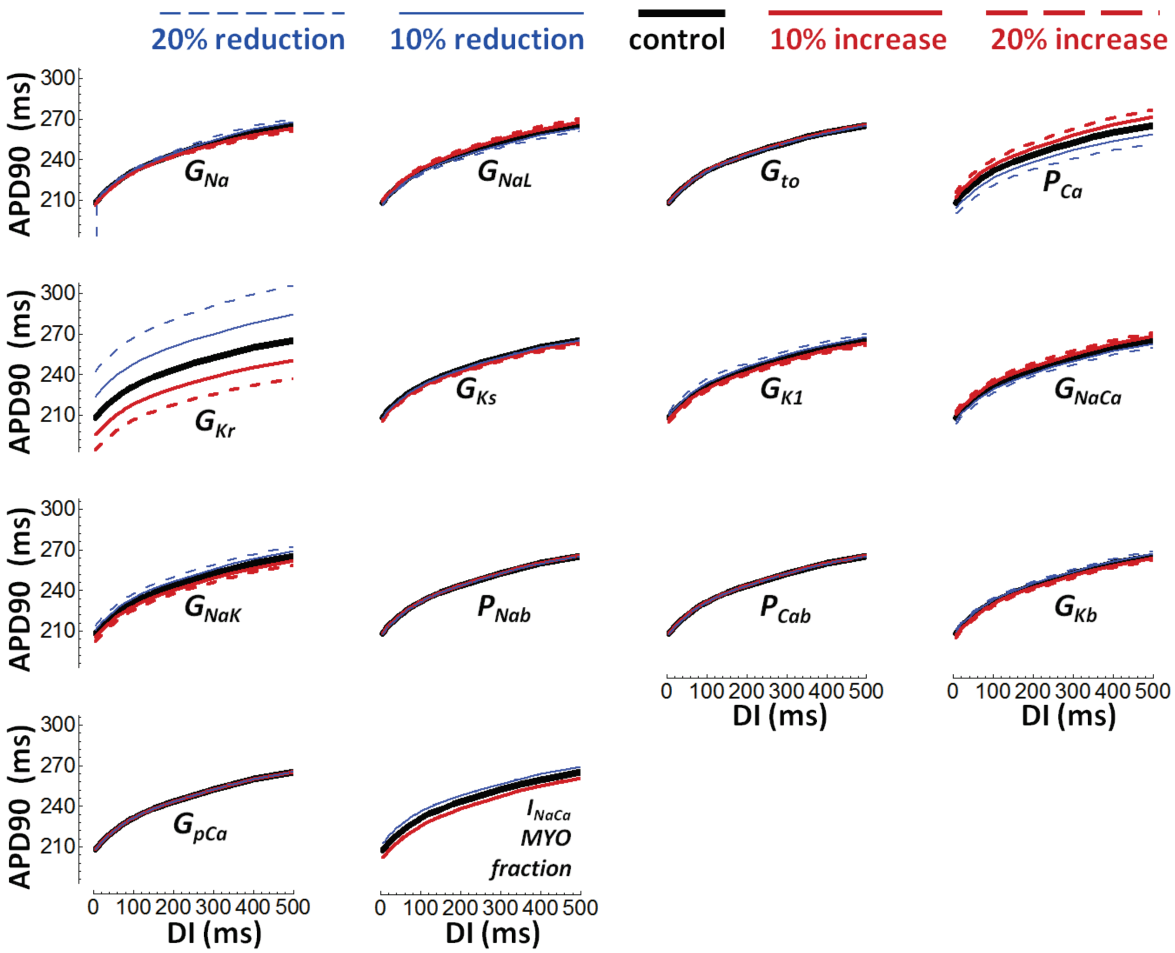

Figure S16. Sensitivity of S1S2 restitution of APD90 to variations in current conductances and to the fraction of I_{NaCa} in the myoplasm (80% in the control case). The control case is shown with the thick black line. Parameter reductions are in blue (20% dashed blue, 10% solid blue). Parameter increases are shown in red (10% solid red, 20% dashed red).

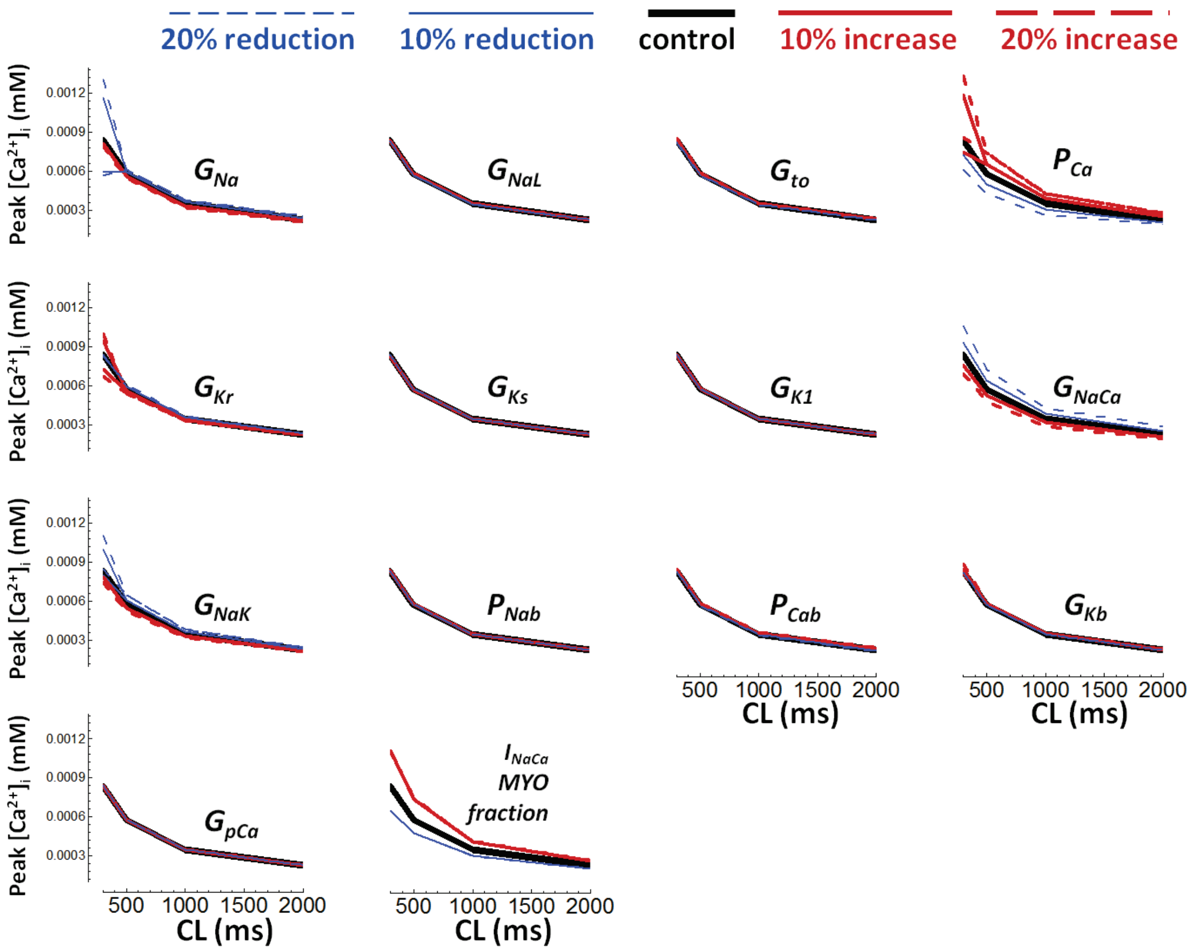

Figure S17. Sensitivity of rate dependence of maximum (systolic) intracellular Ca^{2+} concentration (peak $[Ca^{2+}]_i$) to variations in current conductances and to the fraction of I_{NaCa} in the myoplasm (80% in the control case). The control case is shown with the thick black line. Parameter reductions are in blue (20% dashed blue, 10% solid blue). Parameter increases are shown in red (10% solid red, 20% dashed red).

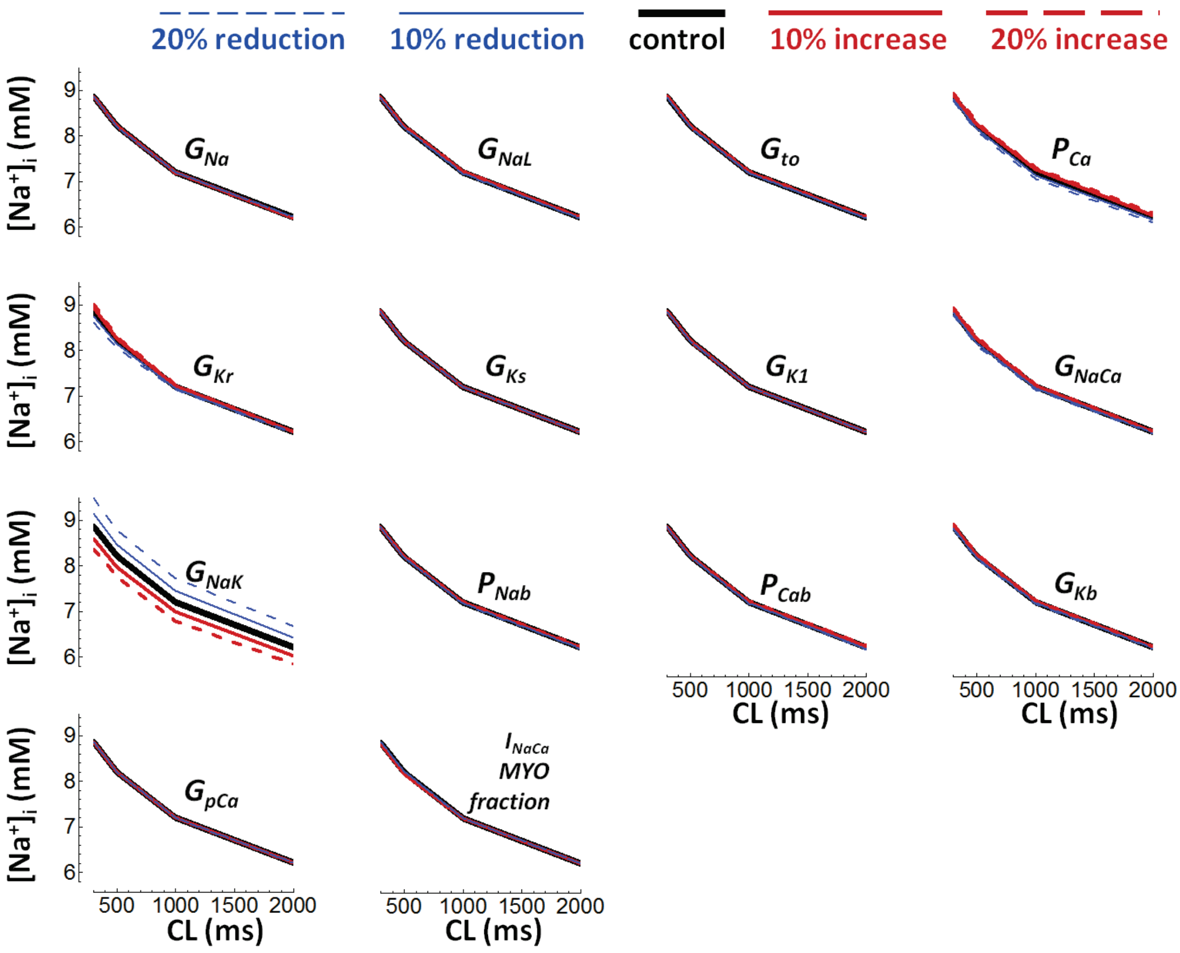

Figure S18. Sensitivity of rate dependence of intracellular Na^+ concentration ($[Na^+]_i$) to variations in current conductances and to the fraction of I_{NaCa} in the myoplasm (80% in the control case). The control case is shown with the thick black line. Parameter reductions are in blue (20% dashed blue, 10% solid blue). Parameter increases are shown in red (10% solid red, 20% dashed red).

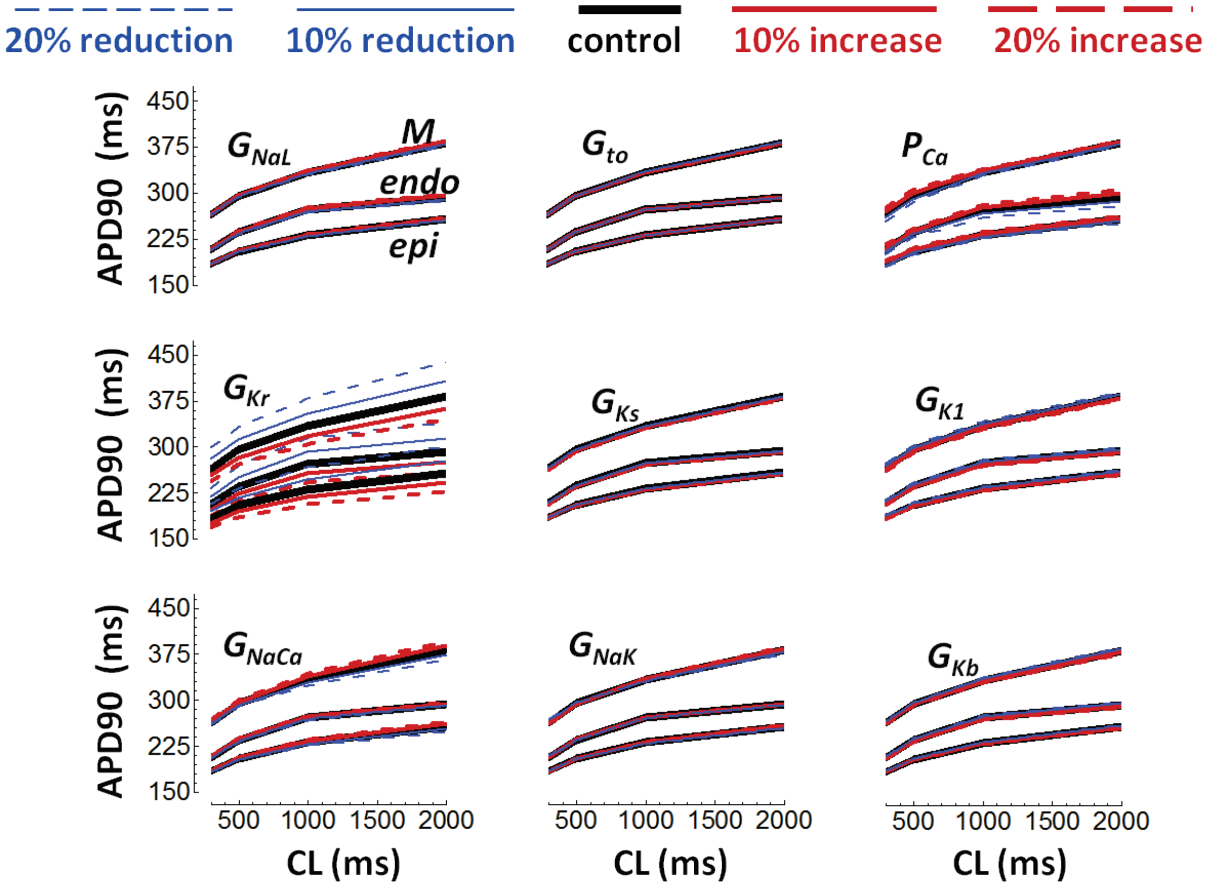

Figure S19. Sensitivity of steady state rate dependence of APD90 in the different transmural cell types to changes in current conductances. The control case is shown with the thick black line. Parameter reductions are in blue (20% dashed blue, 10% solid blue). Parameter increases are shown in red (10% solid red, 20% dashed red).

References

1. Ten Tusscher KH, Panfilov AV. Alternans and spiral breakup in a human ventricular tissue model. *Am J Physiol Heart Circ Physiol.* 2006;291(3):H1088-1100.
2. Grandi E, Pasqualini FS, Bers DM. A novel computational model of the human ventricular action potential and Ca transient. *J Mol Cell Cardiol.* 2010;48(1):112-121.
3. Hund TJ, Kucera JP, Otani NF, Rudy Y. Ionic charge conservation and long-term steady state in the Luo-Rudy dynamic cell model. *Biophys J.* 2001;81(6):3324-3331.
4. Forbes M, Sperelakis N. Ultrastructure of Mammalian Cardiac Muscle. In: Sperelakis N, ed. *Physiology and Pathophysiology of the Heart. 2nd edition*. Boston, MA: Kluwer Academic; 1989:3-41.
5. Decker KF, Heijman J, Silva JR, Hund TJ, Rudy Y. Properties and ionic mechanisms of action potential adaptation, restitution, and accommodation in canine epicardium. *Am J Physiol Heart Circ Physiol.* 2009;296(4):H1017-1026.
6. Hund TJ, Rudy Y. Rate dependence and regulation of action potential and calcium transient in a canine cardiac ventricular cell model. *Circulation.* 2004;110(20):3168-3174.
7. Yue DT, Marban E. A novel cardiac potassium channel that is active and conductive at depolarized potentials. *Pflugers Arch.* 1988;413(2):127-133.
8. Sridhar A, da Cunha DN, Lacombe VA, Zhou Q, Fox JJ, Hamlin RL, Carnes CA. The plateau outward current in canine ventricle, sensitive to 4-aminopyridine, is a constitutive contributor to ventricular repolarization. *Br J Pharmacol.* 2007;152(6):870-879.
9. Pieske B, Maier LS, Piacentino V, 3rd, Weisser J, Hasenfuss G, Houser S. Rate dependence of [Na+]i and contractility in nonfailing and failing human myocardium. *Circulation.* 2002;106(4):447-453.
10. Schmidt U, Hajjar RJ, Helm PA, Kim CS, Doye AA, Gwathmey JK. Contribution of abnormal sarcoplasmic reticulum ATPase activity to systolic and diastolic dysfunction in human heart failure. *J Mol Cell Cardiol.* 1998;30(10):1929-1937.
11. Livshitz LM, Rudy Y. Regulation of Ca2+ and electrical alternans in cardiac myocytes: role of CAMKII and repolarizing currents. *Am J Physiol Heart Circ Physiol.* 2007;292(6):H2854-2866.
12. Witcher DR, Kovacs RJ, Schulman H, Cefali DC, Jones LR. Unique phosphorylation site on the cardiac ryanodine receptor regulates calcium channel activity. *J Biol Chem.* 1991;266(17):11144-11152.
13. Hawkins C, Xu A, Narayanan N. Sarcoplasmic reticulum calcium pump in cardiac and slow twitch skeletal muscle but not fast twitch skeletal muscle undergoes phosphorylation by endogenous and exogenous Ca2+/calmodulin-dependent protein kinase. Characterization of optimal conditions for calcium pump phosphorylation. *J Biol Chem.* 1994;269(49):31198-31206.
14. Toyofuku T, Curotto Kurzydlowski K, Narayanan N, MacLennan DH. Identification of Ser38 as the site in cardiac sarcoplasmic reticulum Ca(2+)-ATPase that is phosphorylated by Ca2+/calmodulin-dependent protein kinase. *J Biol Chem.* 1994;269(42):26492-26496.
15. Sobie EA, Song LS, Lederer WJ. Local recovery of Ca2+ release in rat ventricular myocytes. *J Physiol.* 2005;565(Pt 2):441-447.
16. Rush S, Larsen H. A practical algorithm for solving dynamic membrane equations. *IEEE Trans Biomed Eng.* 1978;25(4):389-392.
17. Livshitz L, Rudy Y. Uniqueness and stability of action potential models during rest, pacing, and conduction using problem-solving environment. *Biophys J.* 2009;97(5):1265-1276.

18. Victorri B, Vinet A, Roberge FA, Drouhard JP. Numerical integration in the reconstruction of cardiac action potentials using Hodgkin-Huxley-type models. *Comput Biomed Res.* 1985;18(1):10-23.
19. Luo CH, Rudy Y. A model of the ventricular cardiac action potential. Depolarization, repolarization, and their interaction. *Circ Res.* 1991;68(6):1501-1526.
20. Kang TM, Hilgemann DW. Multiple transport modes of the cardiac Na+/Ca2+ exchanger. *Nature.* 2004;427(6974):544-548.
21. Weber CR, Piacentino V, 3rd, Houser SR, Bers DM. Dynamic regulation of sodium/calcium exchange function in human heart failure. *Circulation.* 2003;108(18):2224-2229.
22. Jost N, Acsai K, Horvath B, Banyasz T, Baczko I, Bitay M, Bogats G, Nanasi PP. Contribution of I Kr and I K1 to ventricular repolarization in canine and human myocytes: is there any influence of action potential duration? *Basic Res Cardiol.* 2009;104(1):33-41.
23. Smith NP, Crampin EJ. Development of models of active ion transport for whole-cell modelling: cardiac sodium-potassium pump as a case study. *Prog Biophys Mol Biol.* 2004;85(2-3):387-405.
24. King EL, Altman C. A Schematic Method of Deriving the Rate Laws for Enzyme-Catalyzed Reactions. *J. Phys. Chem.* 1956;60:1375-1378.
25. Nakao M, Gadsby DC. [Na] and [K] dependence of the Na/K pump current-voltage relationship in guinea pig ventricular myocytes. *J Gen Physiol.* 1989;94(3):539-565.
26. Spach MS, Heidlage JF, Dolber PC, Barr RC. Electrophysiological effects of remodeling cardiac gap junctions and cell size: experimental and model studies of normal cardiac growth. *Circ Res.* 2000;86(3):302-311.
27. Glukhov AV, Fedorov VV, Lou Q, Ravikumar VK, Kalish PW, Schuessler RB, Moazami N, Efimov IR. Transmural dispersion of repolarization in failing and nonfailing human ventricle. *Circ Res.* 2010;106(5):981-991.
28. Cutler MJ, Wan X, Laurita KR, Hajjar RJ, Rosenbaum DS. Targeted SERCA2a gene expression identifies molecular mechanism and therapeutic target for arrhythmogenic cardiac alternans. *Circ Arrhythm Electrophysiol.* 2009;2(6):686-694.
29. Crampin EJ, Smith NP. A dynamic model of excitation-contraction coupling during acidosis in cardiac ventricular myocytes. *Biophys J.* 2006;90(9):3074-3090.
30. Szabo G, Szentandrassy N, Biro T, Toth BI, Czifra G, Magyar J, Banyasz T, Varro A, Kovacs L, Nanasi PP. Asymmetrical distribution of ion channels in canine and human left-ventricular wall: epicardium versus midmyocardium. *Pflugers Arch.* 2005;450(5):307-316.
31. Nakajo K, Ulbrich MH, Kubo Y, Isacoff EY. Stoichiometry of the KCNQ1 - KCNE1 ion channel complex. *Proc Natl Acad Sci U S A.* 2010;107(44):18862-18867.
32. Sanguinetti MC, Curran ME, Zou A, Shen J, Spector PS, Atkinson DL, Keating MT. Coassembly of K(V)LQT1 and minK (IsK) proteins to form cardiac I(Ks) potassium channel. *Nature.* 1996;384(6604):80-83.
33. Franz MR, Swerdlow CD, Liem LB, Schaefer J. Cycle length dependence of human action potential duration in vivo. Effects of single extrastimuli, sudden sustained rate acceleration and deceleration, and different steady-state frequencies. *J Clin Invest.* 1988;82(3):972-979.
